# Supplementary material for: Molecular mechanism for rapid autoxidation in α-pinene ozonolysis
Source: Nat Commun. 2021 Feb 9;12:878. doi: 10.1038/s41467-021-21172-w (PMC7873275; doi:10.1038/s41467-021-21172-w)
Supplement: Supplementary file 1 — Supplementary Information [file 41467_2021_21172_MOESM1_ESM.pdf]

Supplementary Information

Molecular mechanism for rapid autoxidation in α-pinene ozonolysis

Siddharth Iyer[1,*,+], Matti P. Rissanen[1,*,+], Rashid Valiev[2,3], Shawon Barua[1], Jordan E. Krechmer[4], Joel Thornton[5], Mikael Ehn[6], and Theo Kurtén[2,*]

[1]Aerosol Physics Laboratory, Tampere University, FI-33101 Tampere, Finland
[2]Department of Chemistry, University of Helsinki, P.O. Box 55, FI-00014 Helsinki, Finland
[3]Tomsk State University, 36 Lenin Avenue, Tomsk 634050, Russia
[4]Aerodyne Research, Inc., Billerica, MA 01821, USA
[5]Department of Atmospheric Science, University of Washington Seattle, Washington 98195, USA
[6]Institute of Atmospheric and Earth System Research (INAR/Physics), University of Helsinki, P.O. Box 64, FI-00014 Helsinki, Finland
*Correspondence to: Siddharth Iyer (siddharth.parameswaraniyer@tuni.fi), Matti P. Rissanen (matti.rissanen@tuni.fi), and Theo Kurtén (theo.kurten@helsinki.fi).
[+]Equally contributing authors

Supplementary Methods

All studied molecules were initially built using the Spartan '14 and Spartan '18 programs. Conformer sampling was carried out in Spartan by rotating molecules in 120 degree steps around their torsions. For radical systems, the radical atom, which was either O or C, had to be assigned as uncharged in Spartan, which would otherwise treat the atoms with an unpaired electron as ions, affecting the conformer sampling. This was done by using the keywords FFHINT~~O_n=6 for radical oxygen atom and FFHINT~~C_n=+-0 for radical carbon atom in Spartan during conformer sampling. The MMFF method was used to carry out the sampling. All non-transition state conformers were first optimized at the ωB97X-D/6-31+G(d) level and subsequently conformers within 2 kcal/mol in relative electronic energies were re-optimized at the ωB97X-D/aug-cc-pVTZ level using Gaussian 09 program. For transition states, the approximate TS structure was first built in Spartan, and then optimized at the B3LYP/6-31+G(d) level in Gaussian 09 with the relevant TS bond distances constrained. B3LYP was used for the initial TS calculations as it has been shown to work well in finding TS geometries. Following the constrained optimization of the approximate TS geometry, unconstrained TS optimization was carried out at the same level of theory. Once the TS was found, the geometry was taken back to Spartan to do a conformer sampling with the relevant bonds constrained. The multiple TS conformers were once again optimized with constraints, and subsequently run through a unconstrained TS optimization at the B3LYP/6-31+G(d) level in Gaussian 09. Conformers within 2 kcal/mol in relative electronic energies were optimized at the higher ωB97X-D/aug-cc-pVTZ level. Single point electronic energy calculation at the ROHF-ROCCSD(T)-F12a/VDZ-F12 level were carried out for the lowest energy conformers of both the transition state and non-transition state structures using Molpro program. The final zero-point corrected energies reported here include the coupled-cluster energy corrections.

It should be noted that the transition state and products of the Criegee pathway (discussed in the following subsection) have open shell singlet configurations, and must therefore be treated using the multirefence methods. In order to compare the activation barriers for the Criegee pathway and Vinoxy pathway the same level of theory must be used. Therefore, the reactant, transition state and product for both the Criegee pathway and the vinoxy pathway were optimized using the extended multi-configuration quasi-degenerate perturbation theory at the second order (XMC-QDPT2)[1] with eight electrons in six molecular orbitals (MOs) and with seven electrons in six MOs, respectively. The calculations were performed using Firefly software[2]. The 6-311++G(d,p) basis set was used.

The XMC-QDPT2 calculated barrier for vinoxy ring-breaking reaction (Figure 3 TS4 in main manuscript) is 12.1 kcal/mol above the vinoxy intermediate, which is a good verification of the ROHF-ROCCSD(T)-F12a/VDZ-F12//ωB97X-D/aug-cc-pVTZ level of theory (TS4 = 14.0 kcal/mol above the vinoxy intermediate) used for the PES and consequently the MESMER simulations.

The example MESMER input in Supplementary Note 8 contains all the geometries, vibrational frequencies, and corrected relative energies of the studied species.

As noted in the manuscript, α-pienene ozonolysis can follow 3 distinct pathways, forming 4 possible Criegee intermediates (see Supplementary Figure 1). The ozonolysis of ethene can also follow a diradical channel, but this pathway is restricted to molecules like ethene that are able to rotate in either direction around the C-C bond connected to ozone in the primary ozonide intermediate[3] (see Supplementary Figure 2). In the α-pinene system, this internal rotation is severely hindered by the 6-member ring, and the diradical channel is consequently unimportant. While the manuscript primarily focused on the pathway P1 in Supplementary Figure 1 where only vinoxy isomerization was possible, the P2 pathway opens up a possible Criegee isomerization reaction that can break the cyclobutyl ring of α-pinene. The ring-breaking scheme is shown in Supplementary Figure 3.

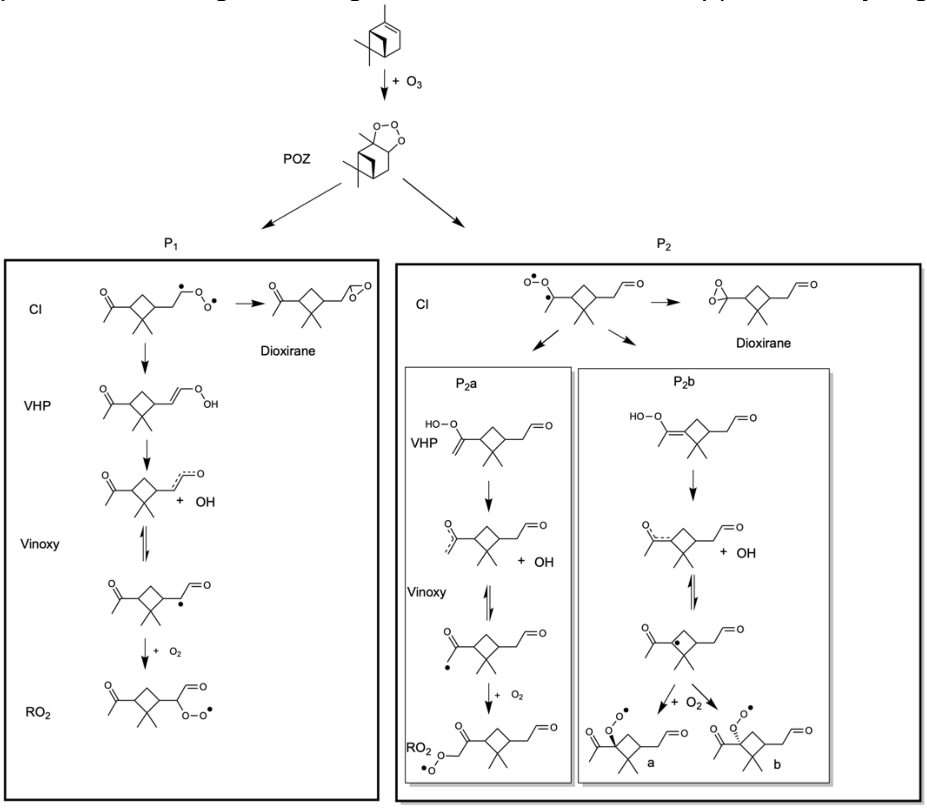

Supplementary Figure 1 - Ozonolysis reaction of α-pinene. The addition of ozone to the double bond creates a primary ozonide (POZ). This can break apart in two ways, form two different Criegee intermediate (CI) isomers and their evolution is depicted along P1 and P2. The CI can do a 1,4 H-shift, forming a vinyl hydroperoxide (VHP). Along P1, the CI has only one possible1,4 H-shift, while it has two along P2 (P2a and P2b). The VHP is assumed to rapidly lose the OH and form a vinoxy radical, which then adds an O_2 to form an RO_2. There are two possible RO_2 isomers along P2b.

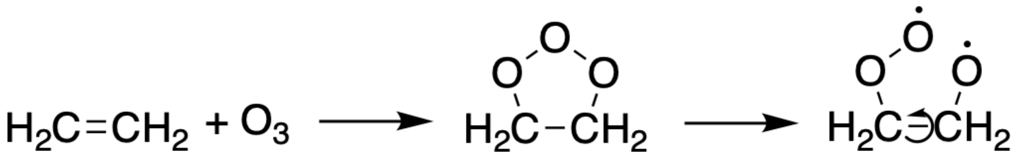

Supplementary Figure 2 - Diradical pathway of ethene ozonolysis. This reaction requires the unhindered rotation of the C-C bond.

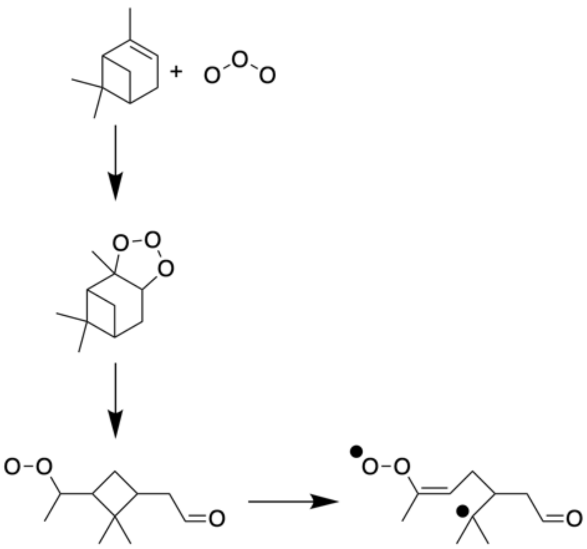

Supplementary Figure 3 – Criegee ring-breaking isomerization pathway.

From preliminary calculations using the SS-CASSCF(8,6)/6-311++G(d,p) method, we found that the wavefunction of the transition state of the Criegee ring-break channel has a multireference character, with two dominant determinants (with weights of -0.78 and 0.56 in the configurational interaction (CI) expansion). A similar situation exists for the product of this channel, where the ground state is best described as an open shell singlet. In both cases, multireference methods are required to treat them accurately. Preliminary calculations on the vinoxy ring-break channel using the SS-CASSCF(7,6)/6-311++G(d,p) method showed that the wavefunctions of the transition state, the reactant, and the product of this channel are all single-reference, with the main determinant having a weight of at least 0.94. However, in order to compare the activation barriers of the Criegee and vinoxy pathways correctly, the same method with similar options should be used. Therefore, we applied XCM-QDPT2 with the same active space and basis set for both pathways (accounting for the loss of one “active” electron to the OH radical in the VHP dissociation reaction forming the vinoxy). We chose the 8 electrons, 6 orbital active space for the Criegee Intermediate, which corresponds to a 7 electron, 6 orbital active space in the vinoxy radical. We chose this active space because it describes both reactions fairly well, and is quite stable, without any rotations or swapping of molecular orbitals in the optimization procedure. The active orbitals for the Criegee Intermediate and vinoxy radical are shown in Supplementary Table 1 and Supplementary Table 2, respectively.

Supplementary Table 1. The stable active space for Criegee ring-breaking isomerization pathway. HOMO = highest occupied molecular orbital, LUMO = lowest unoccupied molecular orbital.

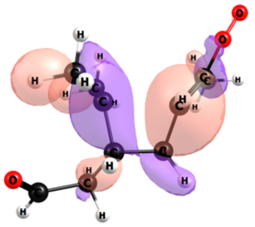	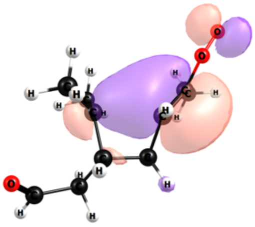

HOMO-3	HOMO-2
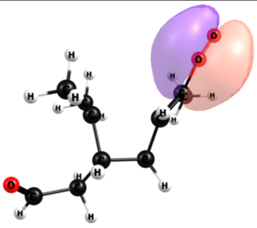<br>HOMO-1	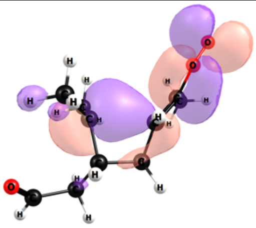<br>HOMO
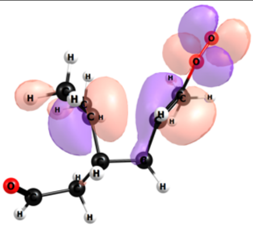<br>LUMO	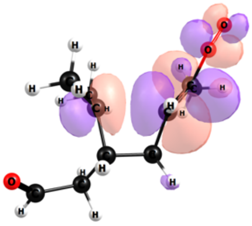<br>LUMO+1

Supplementary Table 2. The stable active space for vinoxy ring-breaking isomerization pathway. HOMO = highest occupied molecular orbital, LUMO = lowest unoccupied molecular orbital, SOMO = singly occupied molecular orbital.

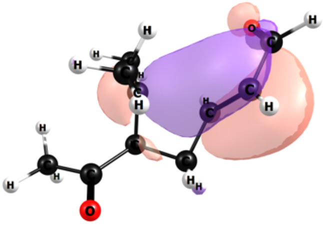<br>HOMO-3	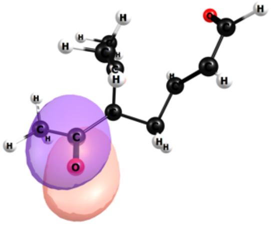<br>HOMO-2
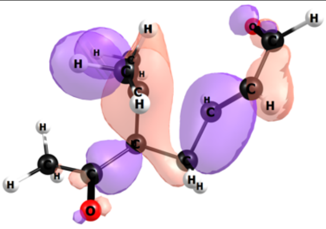<br>HOMO-1	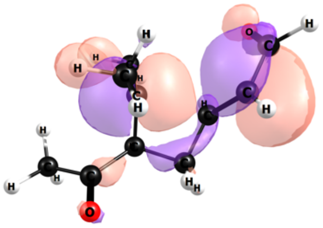<br>SOMO

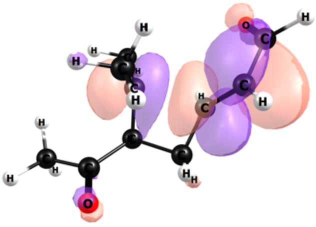 LUMO	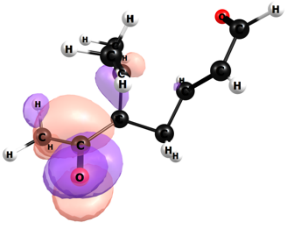 LUMO+1

Geometries of the reaction complex (RC), transition state (TS) and the product (PR) are shown below. Optimizations were carried out at the SS-CASSCF(8,6)/6-311++G(d,p) level of theory. Relative zero-point corrected energies are provided in the following tables.

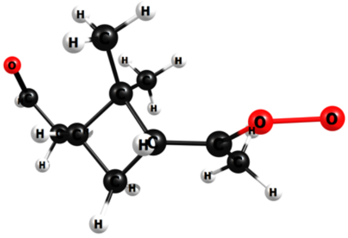

RC

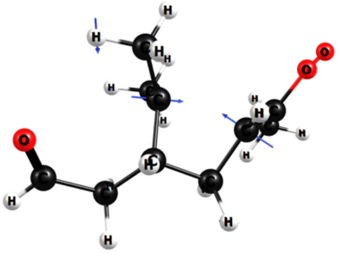

TS

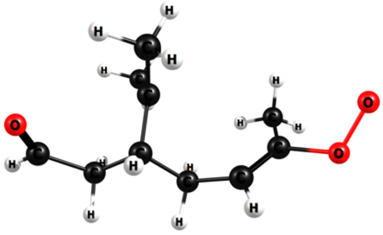

PR

SS-CASSCF(8,6)/6-311++G(d,p)		
0 kcal/mol	42.1 kcal/mol	26.7 kcal/mol
	ω = -520 cm^{-1}	

XMC-QDPT2(8,6)/6-311++G(d,p)		
0 kcal/mol	44.1 kcal/mol	29 kcal/mol
	ω = -600 cm^{-1}	

The high 44.1 kcal/mol barrier for the direct ring-breaking reaction of the Criegee unsurprisingly resulted in zero ring-broken product (structure PR in table above) formed during MESMER simulations.

The vinoxy barrier was also calculated using multireference methods at the XMC-QDPT2(7,6)/6-311++G(d,p) level.

RC	TS	RC	TS
XMC-QDPT2(8,6)/6-311++G(d,p)	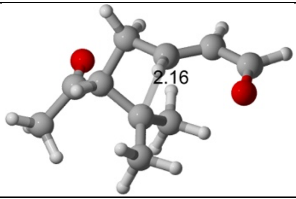	ROHF-ROCCSD(T)-F12a/VDZ-F12//ωB97X-D/aug-cc-pVTZ	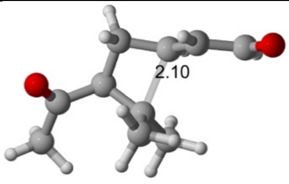
0 kcal/mol	**12.1 kcal/mol**	0 kcal/mol	**14.0 kcal/mol**

	ω = -700 cm^{-1}		ω_{DFT} = -555.45 cm^{-1}

Supplementary Table 3 - Absolute energies in Hartrees of molecules studied. DFT = ωB97X-D/aug-cc-pVTZ; CCSD(T)-F12 = ROHF-ROCCSD(T)-F12*a*/VDZ-F12

**Final Energies**	**DFT - Zero-point corrected energy**	**DFT - Electronic energy**	**CCSD(T)-F12 – Electronic energy**
**α**-pinene	-390.442981	-390.680241	-390.065810
O_3	-225.418552	-225.426363	-225.205905
TS1	-615.851068	-616.098645	-615.263754
POZ	-615.958393	-616.210519	-615.370457
TS2	-615.923545	-616.172441	-615.337146
CI	-615.992896	-616.239934	-615.393073
TS3	-615.967674	-616.210380	-615.363186
VHP	-616.025865	-616.272711	-615.427644
Vinoxy	-540.274335	-540.504244	-539.731471
OH	-75.732142	-75.740745	-75.662874
TS4	-540.251280	-540.478592	-539.706533
RB1	-540.268452	-540.495585	-539.724320
RB1-RO2	-690.642523	-690.880513	-689.959060
TS5	-540.239773	-540.466542	-539.697393
RB2	-540.255222	-540.482088	-539.716354
RB2-RO2	-690.632691	-690.871358	-689.950245
CB-RO2	-690.630931	-690.869729	-689.945433
O_2	-150.330416	-150.334300	-150.175418

Supplementary Table 4 - Reaction energies in kcal/mol relative to **α**-pinene + O_3

Reactions	DFT - Zero-point corrected energy	DFT - Electronic energy	CCSD(T)-F12 – Electronic energy	Final Zero-point corrected energy
**α**-pinene + O_3 => TS1	6.6	5.0	5.0	6.6
**α**-pinene + O_3 => POZ	-60.8	-65.2	-62.0	-57.5
**α**-pinene + O_3 => TS2	-38.9	-41.3	-41.1	-38.7
**α**-pinene + O_3 => CI	-82.4	-83.7	-76.2	-74.9
**α**-pinene + O_3 => TS3	-66.6	-65.1	-57.4	-58.9
**α**-pinene + O_3 => VHP	-103.1	-104.2	-97.8	-96.7
**α**-pinene + O_3 => Vinoxy + OH	-91.0	-86.8	-77.0	-81.1
**α**-pinene + O_3 => TS4 (+OH)	-76.5	-70.7	-61.3	-67.0
**α**-pinene + O_3 => RB1 (+OH)	-87.3	-81.4	-72.5	-78.3
**α**-pinene + O_3 (+O_2) => RB1-RO_2 (+OH)	-114.7	-113.2	-109.7	-111.2

α-pinene + O_3 => TS5 (+OH)	-69.3	-63.2	-55.6	-61.7
α-pinene + O_3 => RB2 (+OH)	-79.0	-72.9	-67.5	-73.5
α-pinene + O_3 (+O_2) => RB2-RO_2 (+OH)	-108.5	-107.4	-104.2	-105.2
α-pinene + O_3 (+O_2) => CB-RO_2 (+OH)	-107.4	-106.4	-101.1	-102.1

Supplementary Note 1. Master equation solver

Master equation solvers are generally not equipped to treat reactions of activated product complexes. It has recently been shown that pseudo-isomerization methodologies can be used to adequately treat multiple activated complex reactions in a single master equation.[4,5] The ozonolysis of α-pinene was the feature of this study, and it leads to the formation of a vinoxy radical with a reaction exothermicity of ~81 kcal/mol. The vinoxy radical can either directly add an O_2 molecule to form a peroxy radical, or isomerize to break the inner 4-member cyclobutyl ring. The latter is a novel mechanism that we report in this work.

The initial association reaction was treated using SimpleRRKM method in MESMER with Eckart tunneling. The other intermediate complexes separated by transition states were also similarly treated. The nascent energy distributed to the vinoxy and OH products from VHP decomposition were approximated using the formulation used by Shannon et al. and will be explained in more detail later.[5] The O_2 addition reaction to vinoxy/alkyl radicals were treated using “Simple Bimolecular Sink” method in MESMER with a bimolecular loss rate coefficient of 2×10^{-12} cm^3 $molecule^{-1}$ s^{-1} and with an O_2 “excess reactant concentration” of 5×10^{18} molecules cm^{-3}. All intermediates were assigned as “modelled” in the simulations and given Lennard-Jones potentials sigma = 6.5 Å and epsilon = 600 K. These are identical to those used by Kurtén et al. for their α-pinene and Δ_3-carene systems.[6] Mesmer utilizes the exponential down (ΔE_{down}) model for simulating the collisional energy transfer. For N_2 bath gas, the MESMER recommended values for ΔE_{down} are between 175-275 cm^{-1}, and a value of 225 cm^{-1} was used in our simulations. Additionally, a grain size of 100 and a value of 60 k_BT for the energy spanned by the grains were used. Ozone was set as the excess reactant and given a high value of 1×10^{18} molecules cm^{-3}. While not relevant to ambient conditions, the high ozone value allowed for rapid formation of products and changes in species profiles in our simulations. Test calculations were run with the more ambiently relevant $[O_3] = 1 \times 10^{12}$ molecules cm^{-3}, and the ratio between CB-RO_2 and RB1-RO_2 fraction populations at the end of the simulation time were near identical to those at the higher $[O_3]$ values. This makes sense since, apart from the initial association reaction, the rest of the ozonolysis steps until the formation of the vinoxy radical are unimolecular. While the formation of the POZ does indeed depend on the initial ozone concentration, the remaining steps do not, and their rates remain the same for the different $[O_3]$ values. Giving a high initial ozone concentration thus allowed us to simulate the interplay between product fractions as a function of time, while keeping the simulation time (and therefore the computation time) feasible.

Ideally, the way to treat post reaction energy distribution in co-products would be to perform classical trajectory calculations. However, this is not computationally feasible for our system size (monoterpene oxidation products with multiple intermediates, TSs and products). Short of that, an approximation can be made of the distribution of the excess energy in the molecule of interest. This can be done in MESMER in two ways.[5] First, the reaction can be

simulated starting from the molecule of interest, which is the vinoxy radical in our case, and initializing the vinoxy with a prior distribution of the energy available from the α-pinene + O_3 reaction. The probability that the vinoxy is formed with an energy E, P(E), is given by:

$$P(E) = \frac{\rho(E)[\rho_t \otimes \rho_{OH}](E_x - E)}{[\rho \otimes \rho_t \otimes \rho_{OH}](E_x)} \qquad (1)$$

where, E_x is the exothermicity of the α-pinene + O_3 reaction, ρ and ρ_{OH} are the rovibrational density of states of the vinoxy and OH, respectively, ρ_t is the relative translational density of states of the co-products vinoxy and OH. This is modelled using a classical expression $\rho_t = \sqrt{E_t}$, where E_t is the relative translational energy. ⊗ represents a convolution (i.e. $[f \otimes g](E) = \int_0^E f(E-x)g(x)dx$). The part of the PES simulated is shown in Supplementary Figure 4.

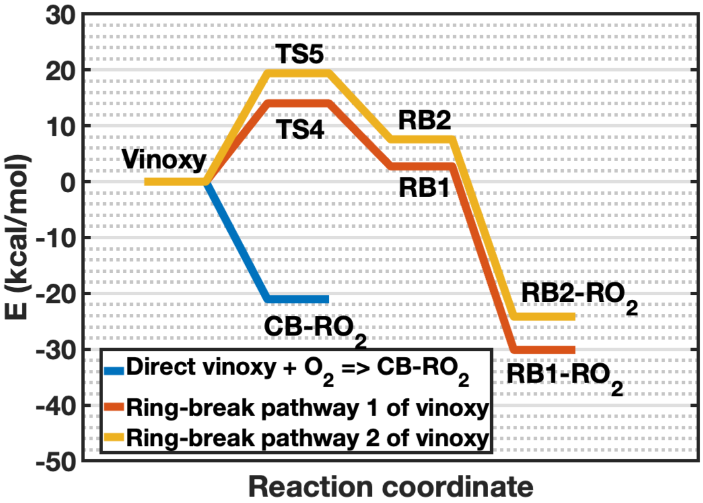

Supplementary Figure 4 - PES starting from the vinoxy radical to the formation of the three RO_2. The vinoxy radical was initialized with a prior energy distribution calculated from an exothermicity Ex of 81.1 kcal/mol. Source data are provided as a Source Data file.

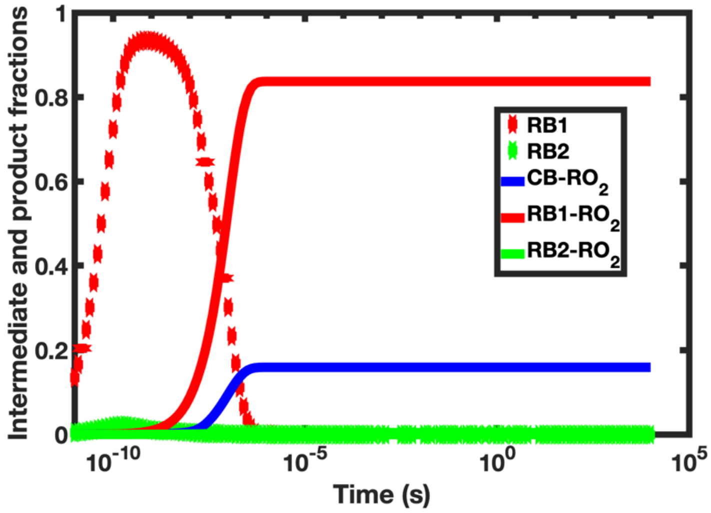

Supplementary Figure 5 - Simulated species profiles as a function of time at T = 298 K and P = 760 Torr. Source data are provided as a Source Data file.

E_x was given a value of 81.1 kcal/mol, which is the exothermicity of the α-pinene + O_3 reaction forming the vinoxy radical. The probability energy distribution of the vinoxy was

calculated using equation 1. This approach resulted in 84% RB1-RO_2 and 16% of CB-RO_2 (as shown in Supplementary Figure 5). The higher barrier to RB2 translated to a near 0 yield (0.4%) of RB2-RO_2. This approach is limited, however, as we would like to include the entire reaction PES starting from α-pinene + O_3, and this method is a poor approximation of the time-dependent energy distribution of the vinoxy fragment.[5]

A second way to simulate activated vinoxy chemistry is by modifying the nascent vinoxy radical energy by following the pseudo-isomerization methodology used by Shannon et al. for their glyoxal + OH system.[5] There are three pseudo-isomerization steps in our reaction: the reversible reaction going from vinoxy + OH to VHP and the 2 reversible reactions going from RB_1 and RB_2 to the vinoxy radical. For a pseudo-isomerization, the dissociation flux, governed by $k(E_i)$ from a grain i in VHP or in RB_1 and RB_2 is partitioned to all grains j of the vinoxy with an energy $E_j \leq E_i$ according to a distribution $Q(E_j|E_i)$ of energy E_j being deposited in the vinoxy fragment. Association rate coefficients are then calculated through detailed balance. The energy distribution in the vinoxy was calculated using the following relation:

$$Q(E_j|E_i) = \frac{(\rho(E_j))^m[\rho_t \otimes \rho_{OH}](E_i - E_x - E_j)}{[(\rho)^m \otimes \rho_t \otimes \rho_{OH}](E_i - E_x)} \qquad (2)$$

The parameter $0 \leq m < 1$ is applied to the density of states of the vinoxy radical and was used to control the energy going into its internal modes. The factor was varied from 0 to 1 in steps of 0.05 in independent simulations. The fraction of the products relative to the precursor α-pinene fraction for the different m values is shown in Supplementary Figure 6.

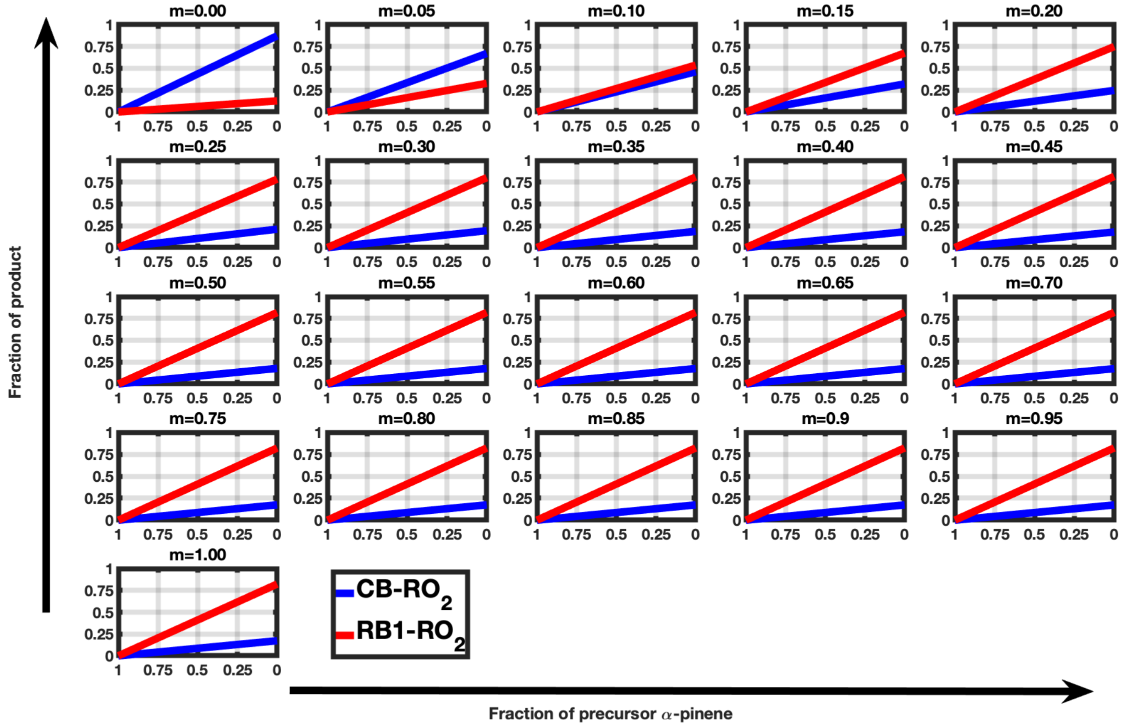

Supplementary Figure 6: Fraction of products CB-RO_2 and RB1-RO_2 as a function of the precursor α-pinene consumed for different m parameter values denoting the fraction of phase space available within vinoxy internal modes to re-distribute reaction exothermicity. Conditions: T = 298 K, P = 760 Torr. Source data are provided as a Source Data file.

As with the simulation that used equation 1, near 0 production of RB_2-RO_2 was observed for all studied m values. Similarly, the high barrier for the direct ring-breaking reaction of the Criegee meant that zero CI_RB were produced. For m=0, only about 12% of RB_1-RO_2 was formed, which was expected as this would mean $\rho(E_j) = 1$ in equation 2. The product fraction of RB_1-RO_2 remained around 82% for m=0.45 and higher, implying 1) energy to the internal modes of the vinoxy corresponding to m=0.45 was sufficient to yield 82% of the ring-broken RO_2, and 2) more energy to the vinoxy did not further increase its yield. The product fractions at the end of the simulation time as a function of the m-parameter is shown in Supplementary Figure 7.

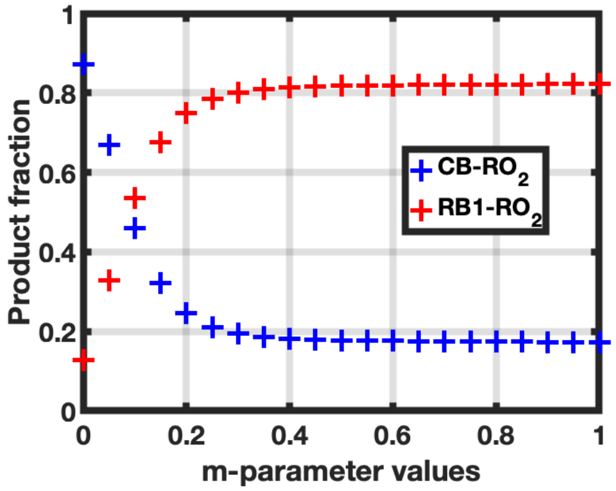

Supplementary Figure 7 - Final CB-RO_2 and RB1-RO_2 fractions as a function of the m-parameter value used during the simulation. Source data are provided as a Source Data file.

The fraction populations of RB1-RO_2 and CB-RO_2 at the final simulation time as a function of temperature and pressure are shown in Supplementary Figure 8. As expected, at higher pressures an increasing fraction of vinoxy is thermalized, and more CB-RO_2 is formed. At T = 350 K, more RB1-RO_2 is formed than at T = 300 K, as the increase in temperature leads to more vinoxy radicals crossing TS4 to form RB1, and consequently RB1-RO_2. A small fraction of α-pinene remains unreacted at the end of the simulation time for T = 250 K due to the barrier for the initial ozonolysis reaction.

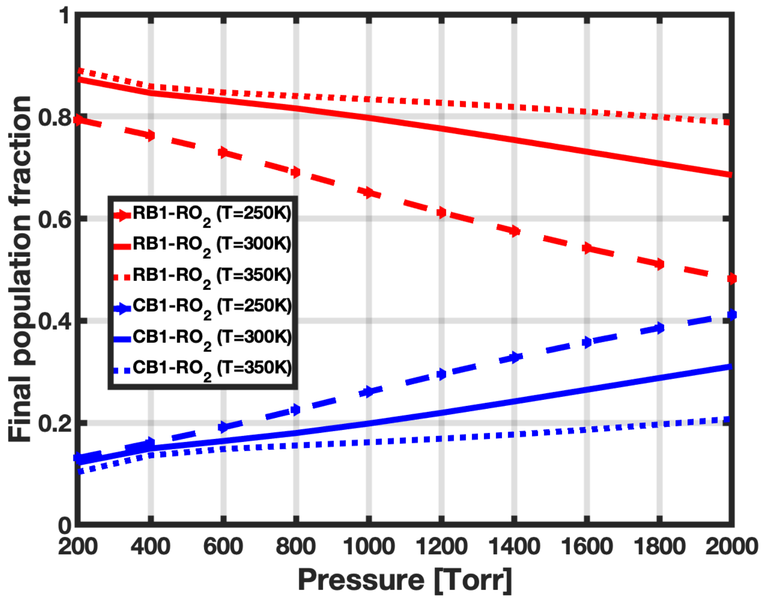

Supplementary Figure 8 - CB-RO$_2$ and RB1-RO$_2$ fractions at the end of the simulation time as a function of pressure and temperature. Source data are provided as a Source Data file.

Pfeifle et al. 2018 carried out trajectory calculations on the ethene + O_3 system and found that for the C_2H_4 + O_3 => CH_2O_2 (Criegee intermediate) + HCOH reaction, 90% of the energy is partitioned into the relative translation energy of the two product fragments.[3] The vinoxy intermediate in our system has 75 internal modes compared to the 12 internal modes of CH_2O_2, and therefore more energy is expected to be partitioned into the rotational and vibrational modes of the vinoxy. (If the relative percentage of energy going into each vibrational mode were roughly similar for the two systems, we would expect slightly less than half of the energy to partition into translational modes for our system). In our simulations, assigning zero internal modes of the vinoxy to spread the excess energy over (in effect replacing the density of states of the vinoxy with that of a single classical harmonic oscillator) still resulted in about 12% ring-broken peroxy radical product (Supplementary Figure 6; m = 0), indicating that some of the translational energy of the vinoxy is converted to useful rotational and vibrational energy following collisions with the bath gas to lead to the ring-breaking isomerization.

Supplementary Note 2. Sensitivity tests

The energetics of ozonolysis reactions are notoriously difficult to accurately calculate using single-reference methods. Therefore, sensitivity tests on the calculated energetics of the α-pinene ozonolysis reaction that were subsequently used in the MESMER simulations were carried out in the following ways, 1. the energetics of ethene ozonolysis were calculated: ethene + O_3 => POZ; ethene + O_3 => to the TS connecting to the Criegee intermediate; using the identical computational procedure that was followed for α-pinene + O_3, and these values were subsequently compared to those from literature, 2. the approximate error margin from step 1 was used to vary the energies of the key TS and intermediates along the PES in the MESMER simulation to report a maximum and minimum RB-RO$_2$ produced, and 3. the energetics of the ring-breaking reaction of the vinoxy was calculated using accurate multireference methods (XMC-QDPT2(8,6)/6-311++G(d,p)).

The calculated energetics of the initial steps of ethene ozonolysis compared to values from literature are shown in Supplementary Table 5.

Supplementary Table 5 - Energies of initial steps of ethene ozonolysis. Comparison between current method and those in literature.

Reaction	E (kcal/mol) (current method)	E (kcal/mol) literature[7] (CCSD(T)/aug-cc-pVTZ//MP4(SDQ)/aug-cc-pVTZ	E (kcal/mol) literature[8, 9] (CCSD(T)/cc-pVTZ	E (kcal/mol) literature[10] (CCSD(T)/6-311G(2d,2p))	E (kcal/mol) literature[3] ANL0-F12
Ethene + O_3 => POZ	-52.7	-56	-50.9	-49.2	-50.2
Ethene + O_3 => TS (to CI)	-33.4	-37	-30.9	-29.5	-32.2

The difference between the energies calculated using the method employed here to those previously calculated in literature was less than 4 kcal/mol. The translation of an error margin of that magnitude on the final product fractions of RB1-RO_2 and CB-RO_2 was investigated by varying the energies of the transition states preceding the vinoxy in the PES by $\pm$5 kcal/mol during the MESMER simulation. Simultaneously, the vinoxy (+OH) energy, which is the excess energy available to the vinoxy to break the ring, was varied between $\pm$10 kcal/mol. These simulations gave us the maximum RB1-RO_2 fraction (when TS energies are -5 kcal/mol and vinoxy energy is +10 kcal/mol relative to the calculated values) and minimum RB1-RO_2 fraction (when TS energies are +5 kcal/mol and vinoxy energy is -10 kcal/mol relative to the calculated values) relative to CB-RO_2. RB1-RO_2 fraction was found to dominate CB-RO_2 also during the "minimum" simulation, and the product fractions were not found to be too sensitive to the TSs (prior to vinoxy) and the vinoxy energies (see Supplementary Table 6).

Supplementary Table 6 - Maximum and minimum ratio of products RB1-RO_2 to CB-RO_2 produced by varying TSs and vinoxy energies during MESMER simulations. m = 0.5, T = 298 K, P = 760 Torr.

Simulation setting	Ratio RB1-RO_2/CB-RO_2
"Minimum": TSs = plus 5 kcal/mol; vinoxy = -10 kcal/mol	2.8
"Maximum": TSs = minus 5 kcal/mol; vinoxy = +10 kcal/mol	5.6

The transition state TS4 controls the rate of formation of RB1-RO_2, and therefore varying that energy during the MESMER simulation should significantly affect the final product fractions. The TS4 energy was varied between -5 to +5 kcal/mol (in steps 1 kcal/mol) relative to the calculated TS4 energy for the MESMER simulations. The RB1-RO_2 and CB-RO_2 product fractions as a function of the relative TS4 energy is shown in Supplementary Figure

9. 0 kcal/mol along the x-axis denotes the TS4 energy calculated in this work (=14.0 kcal/mol relative to vinoxy intermediate). While RB1-RO_2 fraction is sensitive to TS4 energy, which is expected, increasing the calculated barrier height by 5 kcal/mol still produced about 30% RB1-RO_2. As described in Supplementary Methods, the TS4 barrier was also calculated using the more accurate multireference method (XMC-QDPT2(8,6)/6-311++G(d,p) level) and it was found to be lower than the one used in the MESMER simulations (=12.1 kcal/mol compared to 14.0 kcal/mol). The barrier height is therefore unlikely to be significantly higher than the one computed here, and may well be lower.

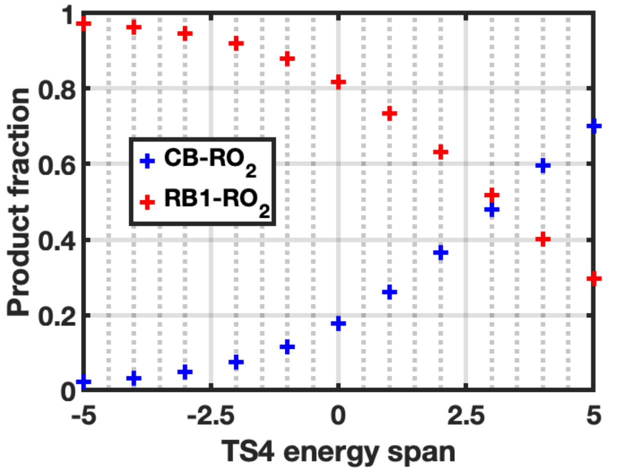

Supplementary Figure 9 - RB1-RO_2 and CB-RO_2 product fractions as a function of relative TS4 energy. Here, 0 kcal/mol denotes the TS4 energy calculated in this work (=14.0 kcal/mol relative to vinoxy intermediate). m = 0.5, T = 298 K, P = 760 Torr. Source data are provided as a Source Data file.

The final RB1-RO_2 fraction can be sensitive to the calculated energy of the ring-broken intermediate RB1 (see Figure 3 in manuscript). An error of a few kcal/mol in the RB1 energy can either reduce or increase the reverse barrier (=11.3 kcal/mol) to the ring-intact vinoxy intermediate. The sensitivity of the RB1-RO_2 fraction produced to this was therefore investigated by running MESMER simulations with the RB1 intermediate energy varied by ±10 kcal/mol. We observed that the RB1-RO_2 fraction is indeed quite sensitive to the RB1 energy (see Supplementary Figure 10 – 0 kcal/mol denotes the RB1 energy calculated in this work = 2.7 kcal/mol relative to vinoxy intermediate).

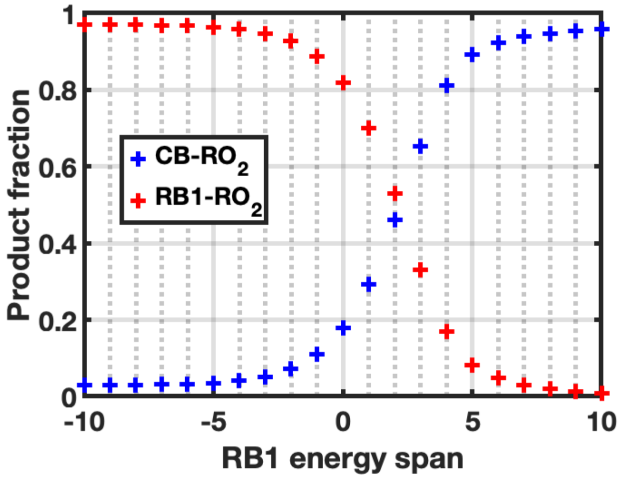

Supplementary Figure 10 - RB1-RO_2 and CB-RO_2 fractions as a function of relative RB1 intermediate energy. Here, 0 kcal/mol denotes the RB1 energy calculated in this work (=2.7

kcal/mol relative to vinoxy intermediate). m = 0.5, T = 298 K, P = 760 Torr. Source data are provided as a Source Data file.

An increase in the RB1 energy by 3 kcal/mol (= reducing the reverse barrier, i.e. RB1 => vinoxy, by 3 kcal/mol) reduced the RB1-RO_2 fraction from 81% to 33% (with the corresponding increase in CB-RO_2). A 5 kcal/mol increase in the RB1 energy still leads to around 8% formation of RB1-RO_2.
The vinoxy intermediate, the RB1 intermediate and the TS4 energies were also calculated using multireference methods at the XMC-QDPT2(8,6)/6-311++G(d,p) level and the RB1 and the TS4 were found to be 4.1 kcal/mol and 12.1 kcal/mol, respectively, relative to the vinoxy intermediate. This equals to a reverse barrier of 8.0 kcal/mol going from the RB1 to the vinoxy intermediate. The reverse barrier is therefore unlikely to be significantly lower than the one computed here. A MESMER simulation with the XMC-QDPT2 relative values for the vinoxy intermediate, TS4 and RB1 produced 52% of the ring-broken peroxy radical RB1-RO_2.

The qualitative conclusion that a significant fraction of the α-pinene + O_3 reaction leads to ring-broken products is thus robust with respect to the likely error margins in the overall ozonolysis PES.

The effect of treating harmonic frequencies as hindered rotors on the final product fractions was investigated by first running computations using Gaussian 16 to identify the hindered rotors, and to calculate the corresponding periodicities and barriers, of all molecules along the studied PES (except the TS and the product of the Criegee ring-break pathway as this channel has no effect on the final RB1-RO_2 and CB-RO_2 fractions) using the Freq=HinderedRotor keyword at the ω97X-D/aug-cc-pVTZ level of theory. The calculations were able to identify the hindered rotors of all molecules except for TS4, RB1 and RB2. For TS4, the hindered rotor calculation identified the number of active rotors = 6, which is the same as that of the vinoxy. As an approximate, we decided to pick the same 6 rotors of the vinoxy for TS4, along with the corresponding periodicities and barriers. For RB1 and RB2, the hindered rotor calculation was found to work with the lower 6-31+G* basis set (ωB97-XD/6-31+G*), and the computed barriers at this level were chosen. For the subsequent MESMER simulation, the HinderedRotorQM1D method was used, which requires the Gaussian derived periodicities and barriers, the bonds corresponding to the hindered rotors and the removal of corresponding harmonic frequencies. The hindered rotor definition section of the MESMER input for the reactant α-pinene is provided below as an example.

```
<me:DOSCMethod xsi:type="me:QMRotors"/>
<!--  Frequency 227.42 has been eliminated in favour of a 1 dimensional free rotor. -->
<me:ExtraDOSCMethod xsi:type="me:HinderedRotorQM1D">
<me:bondRef>b6</me:bondRef>
<me:HinderedRotorPotential format="analytical" units="kJ/mol">
<me:PotentialPoint index="0"  coefficient="6.696932525"/>
<me:PotentialPoint index="3"  coefficient="-6.696932525"/>
</me:HinderedRotorPotential>
<me:periodicity>3</me:periodicity>
</me:ExtraDOSCMethod>

<!--  Frequency 198.34 has been eliminated in favour of a 1 dimensional free rotor. -->
<me:ExtraDOSCMethod xsi:type="me:HinderedRotorQM1D">
```

<me:bondRef>b11</me:bondRef>
<me:HinderedRotorPotential format="analytical" units="kJ/mol">
<me:PotentialPoint index="0" coefficient="5.47356173"/>
<me:PotentialPoint index="3" coefficient="-5.47356173"/>
</me:HinderedRotorPotential>
<me:periodicity>3</me:periodicity>
</me:ExtraDOSCMethod>

Including the hindrance potentials in the MESMER simulation resulted in a final RB1-RO_2 and CB-RO_2 fractions of ~89% and ~11%, respectively, which is an increase from 82% for RB1-RO_2 from the pure harmonic treatment. This indicates that the effect of including internal rotations is surprisingly small – probably due to the very large number of vibrational modes (81 for systems from the POZ to the VHP; 75 for the vinoxy to RB1/RB2, and again 81 for CB-RO_2, RB1-RO_2 and RB2-RO_2), as well as the fact that the number of internal rotational modes changes fairly little across the potential energy surface from the CI onwards (i.e. much of their effects are cancelled out as many of the same rotors are present in the reactants, transition states and products).

We note that our predicted yield of ring-broken peroxy radicals (RB1-RO_2; 89%) is not only qualitatively, but almost quantitatively, in agreement with the experimental result that about 15% of the Criegee Intermediates are thermalized (and thus do not contribute to the formation of RB1-RO_2).

The quantitative yield for RB1-RO_2 from the overall ozonolysis process is difficult to accurately estimate as the yields of the four different Criegee Intermediates from the initial ozone addition reaction to α-pinene are unknown, and estimating them would likely require prohibitively expensive direct dynamics simulations. However, as the formation energetics of the different CIs are to our knowledge reasonably similar, we can crudely assume that each CI will be formed at roughly equal yields, i.e. 25%. The overall yield of RB1 from the α-pinene + O_3 reaction would then be approximately 89% $\times$ 25%, or about 22% according to our best guess, with a "worst-case" lower limit (corresponding to the m=0 simulation) of 12% $\times$ 25% = 3%. Assuming the predicted autoxidation steps from RB1-RO_2 onward are rapid, even this modest yield would be important for atmospheric SOA formation.

Supplementary Note 3. Other ring-break reactions from α-pinene + O_3

Other possible vinoxy ring-breaking reactions along P2 in Supplementary Figure 1 were also investigated. The schematic of the two ring-open mechanisms is shown in Supplementary Figure 11 and the complete α-pinene + O_3 PES with the barriers to ring-broken products RB_3 and RB_4 is shown in Supplementary Figure 12. These calculations were performed at the ωB97X-D/6-31+G(d) level of theory. Further calculations at the higher level of theory were excluded as they are unlikely to change/reduce significantly the high barriers reported below.

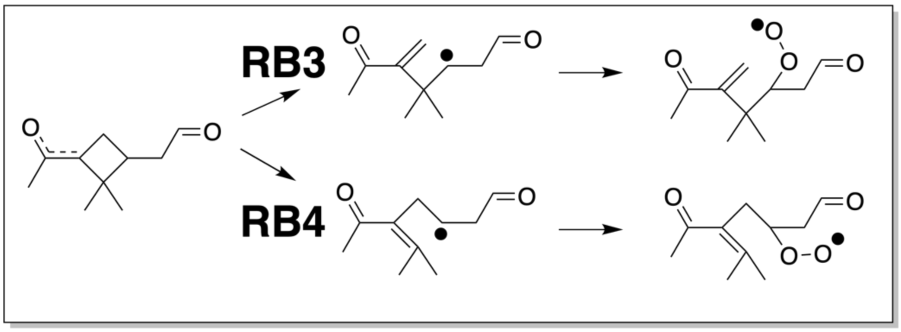

Supplementary Figure 11 – Secondary ring-breaking mechanism. Cyclobutyl ring-breaking isomerization mechanism for vinoxy along P2 in Supplementary Figure 1.

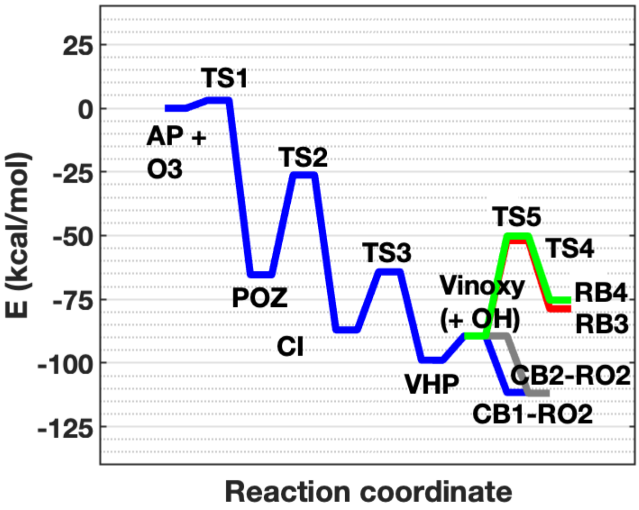

Supplementary Figure 12 – Secondary PES. PES along P2b in Supplementary Figure 1 of the α-pinene + O_3 reaction. Source data are provided as a Source Data file.

The barriers to the two ring-breaking reactions of the vinoxy in this scheme were found to be around 37 kcal/mol, about 2.5 times higher than the barrier forming the tertiary alkyl radical discussed in the main article. This translated to zero ring-broken product formation in our simulation, and a 50/50 divide in the yield of the peroxy radicals CB_1-RO_2 and CB_2-RO_2 with the cyclobutyl ring intact. We can conclude that the fraction of α-pinene ozonolysis reaction that is along P2 will not undergo a rapid cyclobutyl ring break reaction during the ozonolysis process, and would likely involve at least one bimolecular step prior to forming HOM.

Supplementary Note 4. Implications

While α-pinene ozonolysis is one of the most studied reactions in aerosol science, the actual reaction mechanism is still not completely understood. One source of conflict between experiments and theory has been that computationally calculated H-shift (and consequently autoxidation) rates were too slow to explain the observed rapid formation of HOMs from α-pinene ozonolysis in laboratory[11]. By pointing to a novel mechanism that directly breaks the cyclobutyl ring during a-pinene ozonolysis, and showing via master equation simulations that this channel has an appreciable yield, we 1) demystify an important part of the α-pinene ozonolysis chemistry, and 2) to bring theory closer actual observations.

Supplementary note 5. Rapid formation of HOMs from α-pinene ozonolysis

RB1-RO_2 can form either a 6-member of a 7-member ring endoperoxide. Relative rates calculated at the B3LYP/6-31+G(d) level showed that the former is significantly more competitive than the latter (see Supplementary Figure 13). The endoperoxide will subsequently add and O_2, forming a 6-oxygen containing peroxy radical $C_{10}H_{15}O_6$. This peroxy radical has an aldehydic 1,4 H-shift reaction available, with a fast rate of about 2 s^{-1} .[12] A final O_2 addition leads to the 8-oxygen containing peroxy radical $C_{10}H_{15}O_8$.

Multi-conformer transition state theory (MC-TST) rates calculated from quantum chemically calculated energies and partition functions were used to elucidate the preferred endoperoxide channel. Spartan 18' program was used to perform a systematic conformer sampling of the reactants and transition states. First, single-point energies were calculated, and conformers within 5 kcal/mol in relative electronic energies of the lowest energy conformer were subsequently optimized and their frequencies calculated. The computations were carried out at the B3LYP/6-31+G(d) level of theory using Gaussian 09 program.[13] Only conformers with 2 kcal/mol in relative electronic energies were considered for rate calculation. MC-TST rates were calculated as described in Møller et al. 2016.[14] The equation used is shown below:

$$k = \kappa \frac{k_B T}{h} \frac{\sum_i^{all\ TS\ conf.} \exp\left(-\frac{\Delta E_i}{k_B T}\right) Q_{TS,i}}{\sum_j^{all\ reac.\ conf.} \exp\left(-\frac{\Delta E_j}{k_B T}\right) Q_{reac,j}} \exp\left(-\frac{E_{TS,0} - E_{reac,0}}{k_B T}\right),$$

where, k_B is the Boltzmann constant, T = temperature, h = Planck constant, ΔE_i= relative zero-point corrected energy of the TS conformer, ΔE_j = relative zero-point corrected energy of the reactant conformer, $E_{TS,0}$ = zero-point corrected energy of the lowest energy conformer, $E_{react,0}$ = zero-point corrected energy of the lowest energy reactant conformer, $Q_{TS,I}$ = partition function of the TS conformer, $Q_{reac,j}$ = partition function of the reactant conformer. We note that while absolute rates calculated at the B3LYP/6-31+G(d) level have large errors, relative rates are reasonably well predicted by this approach[14,15].

Intrinsic reaction coordinate calculations were conducted at the B3LYP/6-31+G(d) level of theory on the lowest energy TS conformer to find the reactant well and product well energies. These were used to calculate the Eckart tunneling factor, κ. The tunneling factor is calculated by solving the Schrödinger equation for an asymmetrical one-dimensional Eckart potential. It is the exact solution to the approximate potential derived from the reactant well and product well energies, and the imaginary frequency of the TS.

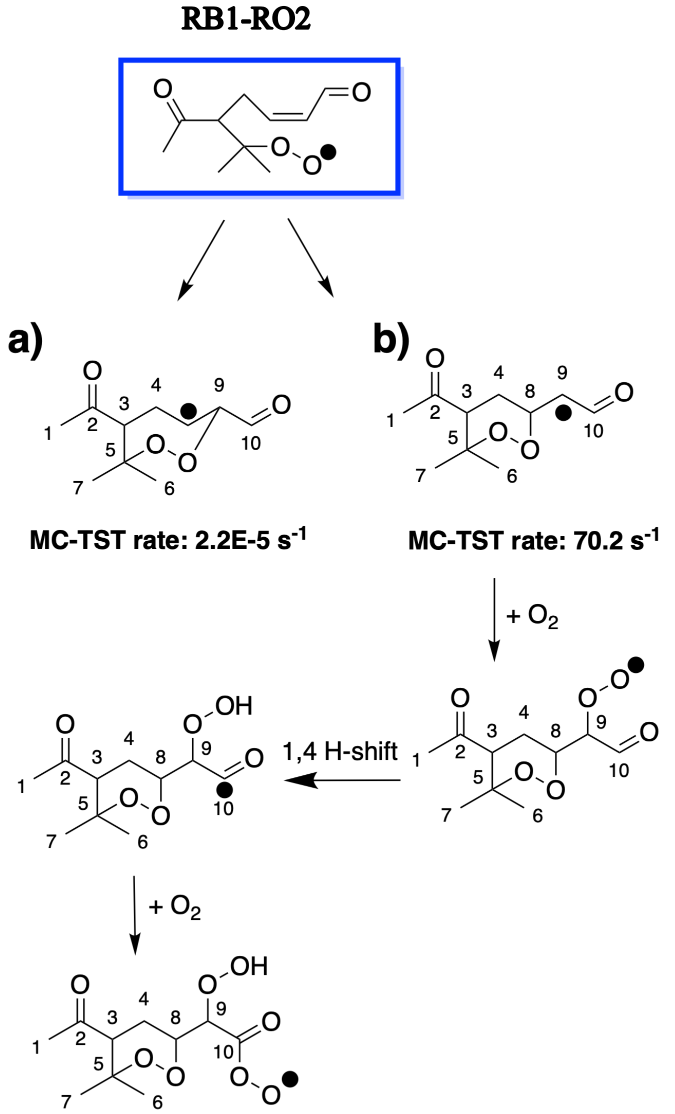

Supplementary Figure 13: Autoxidation steps of RB1-RO_2. Pseudo-unimolecular reactions of RB1-RO_2 leading to a peroxy radical with 8 oxygen atoms following one endoperoxide and one H-shift reaction. a) 7-member ring endoperoxide formation and b) 6-member ring endoperoxide formation. Rates calculated using MC-TST at the B3LYP/6-31+G(d) level of theory.

The rate of the direct 1,8 aldehydic H-shift available to RB1-RO_2 was also investigated at the B3LYP/6-31+G(d) level of theory (see Supplementary Figure 14). This pathway was found to have a large barrier of 32.4 kcal/mol and consequently a slow reaction rate of 2.5×10^{-12} s^{-1}. This reaction can thus safely be ignored despite the likely large error margins of the B3LYP/6-31+G(d) absolute rate.

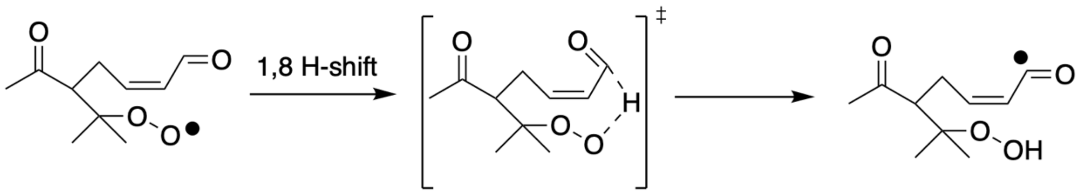

Supplementary Figure 14 - 1,8 aldehydic H-shift available to RB1-RO_2

Supplementary Note 6. Chemical ionization mass spectrometry experiments

Liquid α-pinene (Sigma-Aldrich, purity 91%) bubbled from a reservoir via a N_2 flow and ozone generated by flowing synthetic air through an ozone generator fitted with a 184.9 nm (Hg PenRay) lamp were allowed to interact in a quartz reaction chamber before being detected by a nitrate-based chemical ionization mass spectrometer. The reaction time was controlled by 1) having the O_3 flow through an injector tube within the quartz reaction chamber, limiting the α-pinene and ozone interaction time, and 2) by increasing the inlet flow through the reaction chamber to the mass spectrometer. Nitrate was used as the reagent ion as it is commonly used in HOM formation studies from α-pinene ozonolysis experiments, allowing us to compare our product spectra with those in literature.

Supplementary Note 7. Kinetic modelling of $C_{10}H_{15}O_8$ formation

In addition to the unimolecular pathway, the $C_{10}H_{15}O_8$ peroxy radical can also form from bimolecular channels via alkoxy intermediates.

$RO_2 + RO_2 => RO + RO + O_2$ (RO = alkoxy radical) (7.1)
$RO => H - shift = R'OH$ ($R'OH$ = alkyl radical) (7.2)
$R'OH + O_2 => R''O_2$ ($R''O_2$ = peroxy radical) (7.3)

Kinetic modelling using literature rate constants was carried out using Kinetiscope program[16,17] to estimate the yield of the peroxy radical for the unimolecular and bimolecular pathways.

Unimolecular pathway

As it has been discussed in this paper, the α-pinene + O_3 reaction involves a number of intermediates and transition states that precede the formation of the first-generation peroxy radical. However, for simplicity, the ozonolysis reaction in the kinetic modelling was assumed to directly lead to the first-generation ring-opened peroxy radical $C_{10}H_{15}O_4$ with a rate coefficient of 1×10^{-17} cm^3 $molecule^{-1}$ s^{-1} (literature rate for α-pinene + O_3[18]). The subsequent steps are described in detail in section S5, and is therefore only briefly mentioned here. The peroxy radical $C_{10}H_{15}O_4$ undergoes an endoperoxide formation step (k = 10 s^{-1} [15]), O_2 addition (pseudo-unimolecular rate k = 1×10^7 s^{-1}), 1,4 aldehydic H-shift (k = 2 s^{-1} [12]), and a second O_2 addition (k = 1×10^7 s^{-1}). The simulation time was 1 second.

Reaction steps:

1. α-pinene + O_3 => $C_{10}H_{15}O_4$ (peroxy radical; [α-pinene] = 1×10^{14}; [O_3] = 1×10^{12}; k = 1×10^{-17}).
2. $C_{10}H_{15}O_4$ => Endoperoxide (k = 10).
3. Endoperoxide + O_2 => $C_{10}H_{15}O_6$ (peroxy radical; k = 1×10^7).

4. $C_{10}H_{15}O_6$ => H-shift => $C_{10}H_{14}O_4OOH$ (alkyl radical; k = 2).
5. $C_{10}H_{14}O_4OOH + O_2$ => $C_{10}H_{15}O_8$ (peroxy radical; k = 1×10^7).

The $C_{10}H_{15}O_8$ peroxy radical is formed with 0.2 second simulation time, and reaches a maximum concentration of about 4×10^8 molecules cm^{-3} in the 1 second simulation time. The results (shown in Supplementary Figure 15) indicate that $C_{10}H_{15}O_8$ is formed in significant concentration within 100 ms of reaction time.

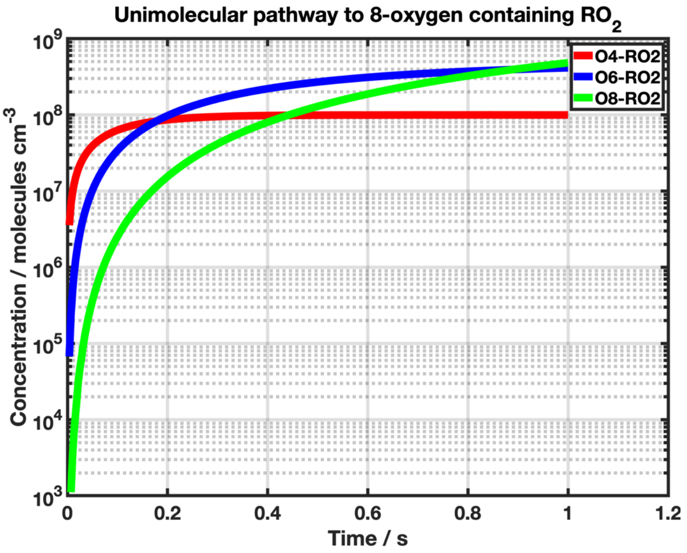

Supplementary Figure 15 - Simulation of the unimolecular pathway to 8-oxygen containing RO_2. Source data are provided as a Source Data file.

Bimolecular pathway

To model the bimolecular pathway, the α-pinene + O_3 reaction was similarly assumed to directly lead to $C_{10}H_{15}O_4$, with a rate coefficient of 1×10^{-17} cm^3 $molecule^{-1}$ s^{-1}. This species was then assumed to react with other peroxy radicals, denoted with the generic name RO_2, and with a starting concentration of 1×10^8 molecules cm^{-3} in the simulation, with a rate coefficient of 8×10^{-11} cm^3 $molecule^{-1}$ s^{-1}. This is the fastest reported $RO_2 + RO_2$ reaction rate .[19] This reaction produces the alkoxy radical $C_{10}H_{15}O_3$ (see equation 7.1). Of the different possible fates of alkoxy radicals, the hydrogen shift is the only pathway that would not directly lead to molecular fragmentation and would allow the molecule to remain a radical. Because both these criteria are essential for the formation of higher $C_{10}H_{15}O_x$ radical (where $x > 3$), we only considered the H-shift pathway in the model (equation 7.2). This has a rate coefficient of about 1×10^6 s^{-1}.[20] This leads to an alkyl radical that adds an O_2 molecule (k = 1×10^7 s^{-1}; reaction 7.3), forming the peroxy radical $C_{10}H_{15}O_5$. The odd-5 oxygen containing peroxy radical cannot form $C_{10}H_{15}O_8$ through autoxidation directly (as autoxidation adds two oxygen atoms to the molecule per step). Therefore, another bimolecular step (equation 7.1) is needed to return to an even number of oxygen atoms. $C_{10}H_{15}O_5$ reacts with RO_2 with a rate coefficient of 8×10^{-11} cm^3 $molecule^{-1}$ s^{-1}, producing $C_{10}H_{15}O_4$. One alkoxy H-shift (k = 1×10^6 s^{-1}), one peroxy H-shift (k = 2 s^{-1}), and two O_2 addition steps (k = 1×10^7 s^{-1}; one after each H-shift) finally leads to the $C_{10}H_{15}O_8$ peroxy radical.

Reaction steps:

1. α-pinene + O_3 => $C_{10}H_{15}O_4$ (peroxy radical) (k = 1×10^{-17})
2. $C_{10}H_{15}O_4 + RO_2$ => $C_{10}H_{15}O_3$ (alkoxy) (k = 8×10^{-11})
3. $C_{10}H_{15}O_3$ => H-shift => $C_{10}H_{14}O_2OH$ (alkyl radical) (k = 1×10^6)

4. $C_{10}H_{14}O_2OH + O_2 => C_{10}H_{15}O_5$ (peroxy radical) ($k = 1\times10^7$)
5. $C_{10}H_{15}O_5 + RO_2 => C_{10}H_{15}O_4$ (alkoxy) ($k = 8\times10^{-11}$)
6. $C_{10}H_{15}O_4$ => H-shift => $C_{10}H_{14}O_3OH$ (alkyl radical) ($k = 1\times10^6$)
7. $C_{10}H_{14}O_3OH + O_2 => C_{10}H_{15}O_6$ ($k = 1\times10^7$)
8. $C_{10}H_{15}O_6$ => H-shift => $C_{10}H_{14}O_4OOH$ (alkyl radical) ($k = 2$)
9. $C_{10}H_{14}O_4OOH + O_2 => C_{10}H_{15}O_8$ ($k = 1\times10^7$)

The second bimolecular $RO_2 + RO_2$ step limits $C_{10}H_{15}O_6$, and consequently, $C_{10}H_{15}O_8$, to low concentrations. Note that $C_{10}H_{15}O_4$ reacts with a generic RO_2 with a concentration set at 1×10^8 molecules cm^{-3} at time = 0. In reality, bimolecular reactions of $C_{10}H_{15}O_4$ would involve reacting with other $C_{10}H_{15}O_4$ or with other first-generation RO_2 produced following α-pinene ozonolysis. This would reduce the rate of $C_{10}H_{15}O_4$ formation and its concentration, affecting the production of the higher oxidized RO_2. Therefore, this simulation should be considered as an upper limit of $C_{10}H_{15}O_8$ produced.

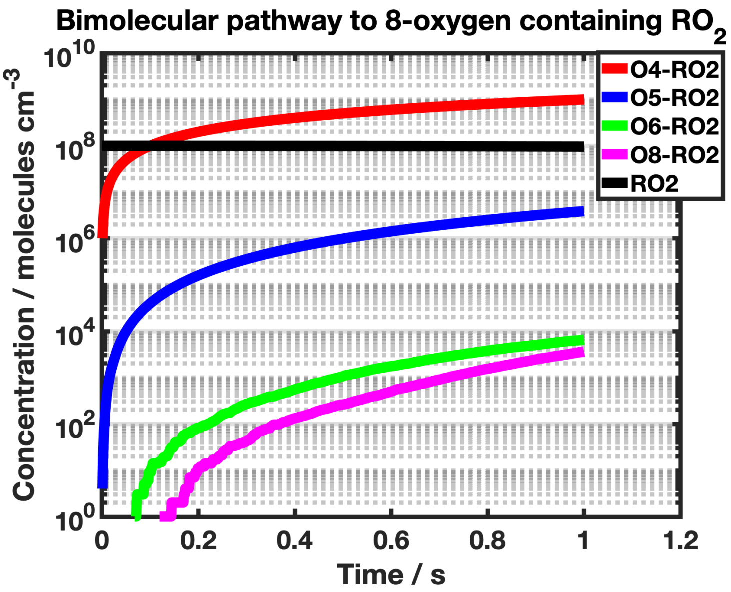

Supplementary Figure 16 - Simulation of the bimolecular pathway to 8-oxygen containing RO_2. Source data are provided as a Source Data file.

If we consider a single bimolecular reaction leading to an alkoxy radical, the subsequent peroxy radicals will have an odd number of oxygen atoms. We checked how highly oxidized the products formed at our experimental conditions could be. The concentrations and rate coefficients were identical to the ones used previously.

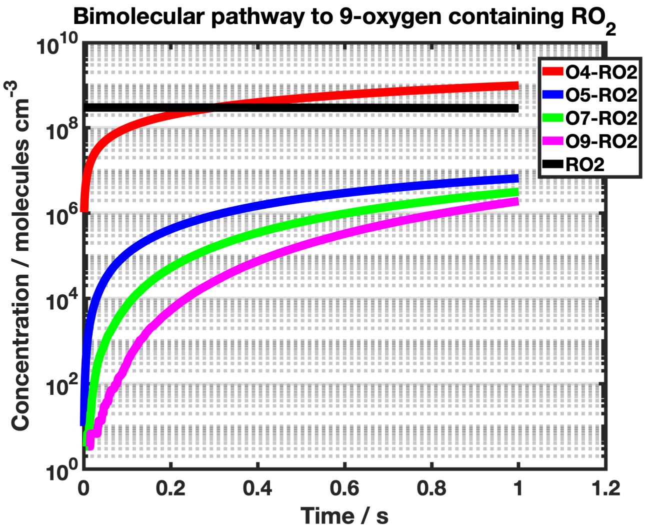

Supplementary Figure 17 - Simulation of the bimolecular pathway to 9-oxygen containing RO_2. Source data are provided as a Source Data file.

Significant concentration of $C_{10}H_{15}O_9$ is produced within the 1 second simulation time. A single RO_2 bimolecular reaction does not therefore limit the rapid formation of highly oxidized molecules. This likely explains the observation of $C_{10}H_{15}O_7$ with 75 ms of our α-pinene ozonolysis experiment (Figure 6 in the manuscript).

Supplementary Note 8. Example MESMER input

The example MESMER input contains all the geometries, vibrational frequencies, and corrected relative energies of the studied species.


```
<?xml version="1.0" encoding="utf-8" ?>
<?xml-stylesheet type='text/xsl' href='../../mesmer2.xsl' media='other'?>
<?xml-stylesheet type='text/xsl' href='../../mesmer1.xsl' media='screen'?>
<me:mesmer xmlns="http://www.xml-cml.org/schema"
xmlns:me="http://www.chem.leeds.ac.uk/mesmer"
xmlns:xsi="http://www.w3.org/2001/XMLSchema-instance">
<me:title> Alphapinene ozonolysis reaction</me:title>
<moleculeList>
<molecule id="AP" description="Reac1">
<atomArray>
<atom id="a1" elementType="C" x3="0.291046" y3="1.800969" z3="0.535790"/>
<atom id="a2" elementType="H" x3="-0.004341" y3="1.997216" z3="1.571462"/>
<atom id="a3" elementType="H" x3="0.526939" y3="2.775506" z3="0.098260"/>
<atom id="a4" elementType="C" x3="1.504026" y3="0.906587" z3="0.506543"/>
<atom id="a5" elementType="C" x3="1.434610" y3="-0.279227" z3="-0.090349"/>
<atom id="a6" elementType="C" x3="0.123149" y3="-0.630628" z3="-0.758740"/>
<atom id="a7" elementType="H" x3="0.146928" y3="-1.604323" z3="-1.248542"/>
<atom id="a8" elementType="C" x3="-1.075328" y3="-0.326782" z3="0.204804"/>
<atom id="a9" elementType="C" x3="-0.863884" y3="1.156798" z3="-0.235328"/>
<atom id="a10" elementType="H" x3="-1.744170" y3="1.800320" z3="-0.257011"/>
<atom id="a11" elementType="C" x3="-0.316880" y3="0.611754" z3="-1.574916"/>
<atom id="a12" elementType="H" x3="0.478028" y3="1.179545" z3="-2.055405"/>
```

<atom id="a13" elementType="H" x3="-1.098678" y3="0.399930" z3="-2.298070"/>
<atom id="a14" elementType="C" x3="-0.956276" y3="-0.682565" z3="1.677987"/>
<atom id="a15" elementType="H" x3="-1.020660" y3="-1.764601" z3="1.811732"/>
<atom id="a16" elementType="H" x3="-1.779708" y3="-0.234289" z3="2.238438"/>
<atom id="a17" elementType="C" x3="-2.391366" y3="-0.898758" z3="-0.317616"/>
<atom id="a18" elementType="H" x3="-2.408020" y3="-1.983018" z3="-0.189912"/>
<atom id="a19" elementType="H" x3="-2.568752" y3="-0.687580" z3="-1.370237"/>
<atom id="a20" elementType="H" x3="-3.229680" y3="-0.486379" z3="0.247790"/>
<atom id="a21" elementType="C" x3="2.551570" y3="-1.269455" z3="-0.144289"/>
<atom id="a22" elementType="H" x3="3.445797" y3="-0.895702" z3="0.353023"/>
<atom id="a23" elementType="H" x3="2.806553" y3="-1.512989" z3="-1.178769"/>
<atom id="a24" elementType="H" x3="2.256088" y3="-2.206345" z3="0.335401"/>
<atom id="a25" elementType="H" x3="-0.022046" y3="-0.347889" z3="2.121621"/>
<atom id="a26" elementType="H" x3="2.411714" y3="1.238429" z3="0.996898"/>
</atomArray>
<bondArray>
<bond atomRefs2="a13 a11" order="1"/>
<bond atomRefs2="a12 a11" order="1"/>
<bond atomRefs2="a11 a6" order="1"/>
<bond atomRefs2="a11 a9" order="1"/>
<bond atomRefs2="a19 a17" order="1"/>
<bond atomRefs2="a7 a6" order="1"/>
<bond atomRefs2="a23 a21" order="1"/>
<bond atomRefs2="a6 a5" order="1"/>
<bond atomRefs2="a6 a8" order="1"/>
<bond atomRefs2="a17 a18" order="1"/>
<bond atomRefs2="a17 a8" order="1"/>
<bond atomRefs2="a17 a20" order="1"/>
<bond atomRefs2="a10 a9" order="1"/>
<bond atomRefs2="a9 a8" order="1"/>
<bond atomRefs2="a9 a1" order="1"/>
<bond atomRefs2="a21 a5" order="1"/>
<bond atomRefs2="a21 a24" order="1"/>
<bond atomRefs2="a21 a22" order="1"/>
<bond atomRefs2="a5 a4" order="2"/>
<bond atomRefs2="a3 a1" order="1"/>
<bond atomRefs2="a8 a14" order="1"/>
<bond atomRefs2="a4 a1" order="1"/>
<bond atomRefs2="a4 a26" order="1"/>
<bond atomRefs2="a1 a2" order="1"/>
<bond atomRefs2="a14 a15" order="1"/>
<bond atomRefs2="a14 a25" order="1"/>
<bond atomRefs2="a14 a16" order="1"/>
</bondArray>
<propertyList>
<property title="File Format">
<scalar>g09</scalar>
</property>
<property dictRef="me:ZPE">
<scalar units="kcal/mol">0.0</scalar>

</property>
<property title="Vibrational Frequencies" dictRef="me:vibFreqs">
<array units="cm-1">123.68 172.79 194.63 203.80 224.33 269.88 312.03 342.40 397.46 407.59 440.79 473.09 491.79 584.42 633.14 684.02 794.38 818.71 844.02 870.23 913.11 935.12 953.29 965.48 982.21 990.06 1026.98 1043.64 1071.22 1074.44 1092.75 1119.27 1141.53 1160.94 1201.52 1221.75 1246.47 1255.81 1265.52 1288.44 1315.68 1351.24 1374.14 1381.39 1411.64 1420.33 1429.09 1483.13 1485.95 1488.85 1494.25 1497.98 1505.77 1523.15 1530.05 1753.70 3027.34 3029.44 3038.39 3042.93 3056.65 3075.55 3082.21 3083.24 3092.45 3097.87 3103.56 3126.99 3131.32 3149.04 3155.76 3173.32
</array>
</property>
<property dictRef="me:rotConsts">
<array units="cm-1">0.065 0.041 0.038</array>
</property>
<property dictRef="me:symmetryNumber">
<scalar>1</scalar>
</property>
<property dictRef="me:spinMultiplicity">
<scalar>1.0</scalar>
</property>
</propertyList>
<me:DOSCMethod>ClassicalRotors</me:DOSCMethod>
</molecule>
<molecule id="Ozone" description="Reac2">
<atomArray>
<atom id="a1" elementType="O" spinMultiplicity="2" x3="0.000000" y3="1.062898" z3="-0.212541"/>
<atom id="a2" elementType="O" x3="0.000000" y3="0.000000" z3="0.425081"/>
<atom id="a3" elementType="O" spinMultiplicity="2" x3="0.000000" y3="-1.062898" z3="-0.212541"/>
</atomArray>
<bondArray>
<bond atomRefs2="a1 a2" order="1"/>
<bond atomRefs2="a3 a2" order="1"/>
</bondArray>
<propertyList>
<property title="File Format">
<scalar>g09</scalar>
</property>
<property dictRef="me:ZPE">
<scalar units="kcal/mol">0.0</scalar>
</property>
<property dictRef="me:spinMultiplicity">
<scalar>1.0</scalar>
</property>
<property title="Vibrational Frequencies" dictRef="me:vibFreqs">
<array units="cm-1">780.91 1315.31 1332.38</array>
</property>
<property title="Rotational Constants" dictRef="me:rotConsts">
<array units="cm-1">3.886 0.466 0.416</array>

</property>
</propertyList>
<me:DOSCMethod>ClassicalRotors</me:DOSCMethod>
</molecule>
<molecule id="TS1" description="TS connecting reactants to INT2">
<atomArray>
<atom id="a1" elementType="C" x3="-0.333976" y3="0.306412" z3="1.871456"/>
<atom id="a2" elementType="H" x3="-0.115590" y3="-0.660677" z3="2.328206"/>
<atom id="a3" elementType="H" x3="-0.435901" y3="1.012138" z3="2.699953"/>
<atom id="a4" elementType="C" spinMultiplicity="2" x3="0.805268" y3="0.782868" z3="1.001939"/>
<atom id="a5" elementType="C" spinMultiplicity="2" x3="0.578397" y3="0.949456" z3="-0.347046"/>
<atom id="a6" elementType="C" x3="-0.823636" y3="0.661006" z3="-0.823789"/>
<atom id="a7" elementType="H" x3="-0.929946" y3="0.808486" z3="-1.897391"/>
<atom id="a8" elementType="C" x3="-1.493494" y3="-0.605854" z3="-0.201574"/>
<atom id="a9" elementType="C" x3="-1.647933" y3="0.263878" z3="1.079704"/>
<atom id="a10" elementType="H" x3="-2.497480" y3="0.059809" z3="1.731514"/>
<atom id="a11" elementType="C" x3="-1.715509" y3="1.498237" z3="0.151071"/>
<atom id="a12" elementType="H" x3="-1.292736" y3="2.432309" z3="0.519489"/>
<atom id="a13" elementType="H" x3="-2.711900" y3="1.687017" z3="-0.234393"/>
<atom id="a14" elementType="C" x3="-0.743713" y3="-1.922203" z3="-0.105396"/>
<atom id="a15" elementType="H" x3="-0.422078" y3="-2.254504" z3="-1.092692"/>
<atom id="a16" elementType="H" x3="-1.404013" y3="-2.687302" z3="0.308171"/>
<atom id="a17" elementType="C" x3="-2.833996" y3="-0.891744" z3="-0.881180"/>
<atom id="a18" elementType="H" x3="-2.663125" y3="-1.375001" z3="-1.844811"/>
<atom id="a19" elementType="H" x3="-3.437506" y3="-0.004676" z3="-1.061142"/>
<atom id="a20" elementType="H" x3="-3.423160" y3="-1.575809" z3="-0.267794"/>
<atom id="a21" elementType="C" x3="1.391699" y3="1.902929" z3="-1.160487"/>
<atom id="a22" elementType="H" x3="2.390496" y3="2.018591" z3="-0.745838"/>
<atom id="a23" elementType="H" x3="0.899289" y3="2.880150" z3="-1.166562"/>
<atom id="a24" elementType="H" x3="1.478211" y3="1.569322" z3="-2.193392"/>
<atom id="a25" elementType="H" x3="0.134700" y3="-1.876638" z3="0.526526"/>
<atom id="a26" elementType="O" spinMultiplicity="2" x3="1.973801" y3="-0.679833" z3="-1.143783"/>
<atom id="a27" elementType="O" x3="2.193051" y3="-1.345903" z3="-0.077110"/>
<atom id="a28" elementType="O" spinMultiplicity="2" x3="2.549013" y3="-0.603662" z3="0.897004"/>
<atom id="a29" elementType="H" x3="1.605179" y3="1.332063" z3="1.473069"/>
</atomArray>
<bondArray>
<bond atomRefs2="a24 a21" order="1"/>
<bond atomRefs2="a7 a6" order="1"/>
<bond atomRefs2="a18 a17" order="1"/>
<bond atomRefs2="a23 a21" order="1"/>
<bond atomRefs2="a21 a22" order="1"/>
<bond atomRefs2="a21 a5" order="1"/>
<bond atomRefs2="a26 a27" order="1"/>
<bond atomRefs2="a15 a14" order="1"/>
<bond atomRefs2="a19 a17" order="1"/>

<bond atomRefs2="a17 a20" order="1"/>
<bond atomRefs2="a17 a8" order="1"/>
<bond atomRefs2="a6 a5" order="1"/>
<bond atomRefs2="a6 a8" order="1"/>
<bond atomRefs2="a6 a11" order="1"/>
<bond atomRefs2="a5 a4" order="1"/>
<bond atomRefs2="a13 a11" order="1"/>
<bond atomRefs2="a8 a14" order="1"/>
<bond atomRefs2="a8 a9" order="1"/>
<bond atomRefs2="a14 a16" order="1"/>
<bond atomRefs2="a14 a25" order="1"/>
<bond atomRefs2="a27 a28" order="1"/>
<bond atomRefs2="a11 a12" order="1"/>
<bond atomRefs2="a11 a9" order="1"/>
<bond atomRefs2="a4 a29" order="1"/>
<bond atomRefs2="a4 a1" order="1"/>
<bond atomRefs2="a9 a10" order="1"/>
<bond atomRefs2="a9 a1" order="1"/>
<bond atomRefs2="a1 a2" order="1"/>
<bond atomRefs2="a1 a3" order="1"/>
</bondArray>
<propertyList>
<property title="File Format">
<scalar>g09</scalar>
</property>
<property dictRef="me:ZPE">
<scalar units="kcal/mol">6.568219638</scalar>
</property>
<property dictRef="me:rotConsts">
<array units="cm-1">0.040 0.023 0.021</array>
</property>
<property dictRef="me:symmetryNumber">
<scalar>1</scalar>
</property>
<property dictRef="me:vibFreqs">
<array units="cm-1">78.72 103.71 151.84 178.97 198.34 206.15 227.42 239.55 258.92 272.09 333.16 351.92 399.78 429.15 451.55 468.85 502.58 513.66 584.36 622.65 687.05 766.75 805.15 836.80 867.39 883.64 894.25 925.32 952.71 965.29 979.37 996.10 1029.81 1050.81 1055.99 1060.69 1094.83 1105.24 1137.87 1148.24 1174.13 1197.30 1207.58 1224.40 1235.11 1256.93 1265.40 1288.55 1315.32 1345.06 1376.31 1378.72 1415.46 1418.77 1433.88 1479.69 1481.46 1492.57 1493.02 1498.33 1509.73 1523.32 1527.17 1577.02 3036.45 3045.56 3049.50 3053.25 3081.14 3084.12 3087.86 3101.36 3109.11 3111.41 3116.37 3135.75 3153.71 3161.98 3193.46 3220.60</array>
</property>
<property dictRef="me:imFreqs">
<array units="cm-1">258.17</array>
</property>
</propertyList>
<me:DOSCMethod>ClassicalRotors</me:DOSCMethod>
</molecule>

```
<molecule id="INT1" description="First Intermediate">
<atomArray>
<atom id="a1" elementType="C" x3="-0.709713" y3="0.677865" z3="1.786017"/>
<atom id="a2" elementType="H" x3="-0.255752" y3="-0.109707" z3="2.405244"/>
<atom id="a3" elementType="H" x3="-1.007304" y3="1.475415" z3="2.481544"/>
<atom id="a4" elementType="C" x3="0.294396" y3="1.215116" z3="0.789006"/>
<atom id="a5" elementType="C" x3="0.043347" y3="1.117916" z3="-0.524120"/>
<atom id="a6" elementType="C" x3="-1.285564" y3="0.496726" z3="-0.915660"/>
<atom id="a7" elementType="H" x3="-1.434148" y3="0.464440" z3="-2.000836"/>
<atom id="a8" elementType="C" x3="-1.549936" y3="-0.821926" z3="-0.105787"/>
<atom id="a9" elementType="C" x3="-1.953287" y3="0.141988" z3="1.058336"/>
<atom id="a10" elementType="H" x3="-2.714411" y3="-0.211221" z3="1.764053"/>
<atom id="a11" elementType="C" x3="-2.370650" y3="1.167784" z3="-0.025762"/>
<atom id="a12" elementType="H" x3="-2.222220" y3="2.227489" z3="0.204026"/>
<atom id="a13" elementType="H" x3="-3.389050" y3="1.018121" z3="-0.388500"/>
<atom id="a14" elementType="C" x3="-0.407573" y3="-1.807364" z3="0.116678"/>
<atom id="a15" elementType="H" x3="-0.096014" y3="-2.255277" z3="-0.835148"/>
<atom id="a16" elementType="H" x3="-0.732998" y3="-2.620234" z3="0.777998"/>
<atom id="a17" elementType="C" x3="-2.746462" y3="-1.603018" z3="-0.659685"/>
<atom id="a18" elementType="H" x3="-2.466519" y3="-2.115622" z3="-1.588381"/>
<atom id="a19" elementType="H" x3="-3.614642" y3="-0.975643" z3="-0.879196"/>
<atom id="a20" elementType="H" x3="-3.065062" y3="-2.368045" z3="0.059358"/>
<atom id="a21" elementType="C" x3="0.932040" y3="1.651765" z3="-1.607437"/>
<atom id="a22" elementType="H" x3="1.864541" y3="2.056828" z3="-1.203551"/>
<atom id="a23" elementType="H" x3="0.418583" y3="2.446607" z3="-2.165407"/>
<atom id="a24" elementType="H" x3="1.184937" y3="0.866525" z3="-2.330061"/>
<atom id="a25" elementType="H" x3="0.469815" y3="-1.340172" z3="0.565742"/>
<atom id="a26" elementType="O" spinMultiplicity="2" x3="2.890308" y3="-0.922809" z3="-
0.905440"/>
<atom id="a27" elementType="O" x3="3.104365" y3="-0.939833" z3="0.324131"/>
<atom id="a28" elementType="O" spinMultiplicity="2" x3="3.303326" y3="0.152931"
z3="0.894276"/>
<atom id="a29" elementType="H" x3="1.196665" y3="1.697060" z3="1.159867"/>
</atomArray>
<bondArray>
<bond atomRefs2="a24 a21" order="1"/>
<bond atomRefs2="a23 a21" order="1"/>
<bond atomRefs2="a7 a6" order="1"/>
<bond atomRefs2="a21 a22" order="1"/>
<bond atomRefs2="a21 a5" order="1"/>
<bond atomRefs2="a18 a17" order="1"/>
<bond atomRefs2="a6 a5" order="1"/>
<bond atomRefs2="a6 a8" order="1"/>
<bond atomRefs2="a6 a11" order="1"/>
<bond atomRefs2="a26 a27" order="1"/>
<bond atomRefs2="a19 a17" order="1"/>
<bond atomRefs2="a15 a14" order="1"/>
<bond atomRefs2="a17 a8" order="1"/>
<bond atomRefs2="a17 a20" order="1"/>
<bond atomRefs2="a5 a4" order="2"/>
```

<bond atomRefs2="a13 a11" order="1"/>
<bond atomRefs2="a8 a14" order="1"/>
<bond atomRefs2="a8 a9" order="1"/>
<bond atomRefs2="a11 a12" order="1"/>
<bond atomRefs2="a11 a9" order="1"/>
<bond atomRefs2="a14 a25" order="1"/>
<bond atomRefs2="a14 a16" order="1"/>
<bond atomRefs2="a27 a28" order="1"/>
<bond atomRefs2="a4 a29" order="1"/>
<bond atomRefs2="a4 a1" order="1"/>
<bond atomRefs2="a9 a10" order="1"/>
<bond atomRefs2="a9 a1" order="1"/>
<bond atomRefs2="a1 a2" order="1"/>
<bond atomRefs2="a1 a3" order="1"/>
</bondArray>
<propertyList>
<property title="File Format">
<scalar>g09</scalar>
</property>
<property dictRef="me:ZPE">
<scalar units="kcal/mol">-2.07</scalar>
</property>
<property dictRef="me:rotConsts">
<array units="cm-1">0.040 0.017 0.016</array>
</property>
<property dictRef="me:symmetryNumber">
<scalar>1</scalar>
</property>
<property dictRef="me:vibFreqs">
<array units="cm-1">11.06 25.18 41.43 57.86 73.98 94.65 140.25 162.42 202.19 223.87 232.44 268.67 314.47 354.50 394.90 401.94 442.98 470.70 492.33 584.62 632.67 686.88 768.07 800.99 826.56 848.68 876.71 914.51 938.71 960.55 961.81 987.10 991.59 1034.34 1053.13 1078.11 1081.68 1100.43 1123.59 1149.85 1168.99 1209.04 1229.64 1252.58 1263.59 1275.73 1297.70 1324.64 1327.85 1343.94 1363.52 1387.41 1393.60 1426.62 1446.15 1448.26 1507.07 1507.73 1509.23 1519.00 1523.19 1526.52 1545.34 1555.15 1745.92 3039.23 3047.06 3053.18 3059.98 3070.23 3087.56 3094.34 3105.77 3106.95 3116.41 3121.90 3147.85 3151.70 3169.02 3178.88 3200.68</array>
</property>
<property dictRef="me:spinMultiplicity">
<scalar>1</scalar>
</property>
<property dictRef="me:epsilon">
<scalar>600.00</scalar>
</property>
<property dictRef="me:sigma">
<scalar>6.5</scalar>
</property>
</propertyList>
<me:energyTransferModel xsi:type="me:ExponentialDown">
<me:deltaEDown units="cm-1">225.0</me:deltaEDown>

</me:energyTransferModel>
<me:DOSCMethod>ClassicalRotors</me:DOSCMethod>
</molecule>
<molecule id="TS2" description="TS connecting INT2 and CI">
<atomArray>
<atom id="a1" elementType="H" x3="-0.409868" y3="3.028807" z3="-0.070745"/>
<atom id="a2" elementType="C" x3="-0.763859" y3="2.193409" z3="0.535862"/>
<atom id="a3" elementType="H" x3="-1.792391" y3="2.399495" z3="0.825844"/>
<atom id="a4" elementType="H" x3="-0.142286" y3="2.137448" z3="1.423485"/>
<atom id="a5" elementType="C" x3="-0.755041" y3="0.937910" z3="-0.329307"/>
<atom id="a6" elementType="C" x3="0.594483" y3="0.483924" z3="-0.924019"/>
<atom id="a7" elementType="H" x3="0.868292" y3="1.226919" z3="-1.675479"/>
<atom id="a8" elementType="C" x3="0.531139" y3="-0.988641" z3="-1.370558"/>
<atom id="a9" elementType="C" x3="1.148529" y3="-1.354151" z3="-0.003368"/>
<atom id="a10" elementType="C" x3="1.745806" y3="0.076411" z3="0.055854"/>
<atom id="a11" elementType="C" x3="-1.084248" y3="-0.560314" z3="0.876883"/>
<atom id="a12" elementType="H" x3="-1.369484" y3="0.088481" z3="1.698305"/>
<atom id="a13" elementType="O" spinMultiplicity="2" x3="-1.744974" y3="0.856010" z3="-
1.126785"/>
<atom id="a14" elementType="O" x3="-2.107119" y3="-1.105507" z3="0.224752"/>
<atom id="a15" elementType="O" spinMultiplicity="2" x3="-3.022457" y3="-0.233608" z3="-
0.083443"/>
<atom id="a16" elementType="C" x3="0.042660" y3="-1.554387" z3="1.035029"/>
<atom id="a17" elementType="H" x3="-0.385581" y3="-2.549294" z3="0.905450"/>
<atom id="a18" elementType="H" x3="0.445438" y3="-1.504191" z3="2.045846"/>
<atom id="a19" elementType="H" x3="1.855943" y3="-2.182185" z3="0.040358"/>
<atom id="a20" elementType="C" x3="3.084839" y3="0.155006" z3="-0.678857"/>
<atom id="a21" elementType="H" x3="3.860331" y3="-0.362351" z3="-0.111220"/>
<atom id="a22" elementType="H" x3="3.390726" y3="1.196768" z3="-0.790930"/>
<atom id="a23" elementType="H" x3="3.048858" y3="-0.287129" z3="-1.672794"/>
<atom id="a24" elementType="C" x3="1.935330" y3="0.714549" z3="1.423963"/>
<atom id="a25" elementType="H" x3="2.760786" y3="0.219774" z3="1.940189"/>
<atom id="a26" elementType="H" x3="1.064255" y3="0.641941" z3="2.070614"/>
<atom id="a27" elementType="H" x3="2.192035" y3="1.770503" z3="1.330076"/>
<atom id="a28" elementType="H" x3="-0.459127" y3="-1.375358" z3="-1.599630"/>
<atom id="a29" elementType="H" x3="1.190650" y3="-1.207086" z3="-2.204453"/>
</atomArray>
<bondArray>
<bond atomRefs2="a29 a8" order="1"/>
<bond atomRefs2="a7 a6" order="1"/>
<bond atomRefs2="a23 a20" order="1"/>
<bond atomRefs2="a28 a8" order="1"/>
<bond atomRefs2="a8 a6" order="1"/>
<bond atomRefs2="a8 a9" order="1"/>
<bond atomRefs2="a13 a5" order="1"/>
<bond atomRefs2="a6 a5" order="1"/>
<bond atomRefs2="a6 a10" order="1"/>
<bond atomRefs2="a22 a20" order="1"/>
<bond atomRefs2="a20 a21" order="1"/>
<bond atomRefs2="a20 a10" order="1"/>

<bond atomRefs2="a5 a2" order="1"/>
<bond atomRefs2="a5 a11" order="1"/>
<bond atomRefs2="a15 a14" order="1"/>
<bond atomRefs2="a1 a2" order="1"/>
<bond atomRefs2="a9 a19" order="1"/>
<bond atomRefs2="a9 a10" order="1"/>
<bond atomRefs2="a9 a16" order="1"/>
<bond atomRefs2="a10 a24" order="1"/>
<bond atomRefs2="a14 a11" order="1"/>
<bond atomRefs2="a2 a3" order="1"/>
<bond atomRefs2="a2 a4" order="1"/>
<bond atomRefs2="a11 a16" order="1"/>
<bond atomRefs2="a11 a12" order="1"/>
<bond atomRefs2="a17 a16" order="1"/>
<bond atomRefs2="a16 a18" order="1"/>
<bond atomRefs2="a27 a24" order="1"/>
<bond atomRefs2="a24 a25" order="1"/>
<bond atomRefs2="a24 a26" order="1"/>
</bondArray>
<propertyList>
<property title="File Format">
<scalar>g09</scalar>
</property>
<property dictRef="me:ZPE">
<scalar units="kcal/mol">-38.65835857</scalar>
</property>
<property dictRef="me:rotConsts">
<array units="cm-1">0.046 0.023 0.021</array>
</property>
<property dictRef="me:symmetryNumber">
<scalar>1</scalar>
</property>
<property dictRef="me:vibFreqs">
<array units="cm-1">89.91 139.96 190.60 206.31 226.09 234.55 256.62 289.45 320.25 352.57 367.27 393.56 399.44 422.79 435.51 499.16 535.61 554.72 573.09 599.02 650.16 683.24 770.83 844.54 868.52 877.25 919.70 947.20 953.62 965.59 977.97 992.16 1023.85 1045.31 1047.94 1089.08 1096.24 1127.55 1162.20 1176.08 1198.61 1219.27 1234.37 1246.36 1257.14 1271.67 1287.95 1310.11 1320.60 1341.54 1376.31 1400.24 1410.35 1415.78 1429.64 1448.71 1482.22 1490.65 1493.26 1499.51 1501.62 1515.02 1525.08 1530.92 3042.71 3047.30 3061.60 3066.50 3070.16 3088.04 3106.92 3110.09 3111.56 3120.01 3134.97 3135.88 3141.56 3157.41 3163.38 3182.12</array>
</property>
<property dictRef="me:imFreqs">
<array units="cm-1">521.23</array>
</property>
</propertyList>
<me:DOSCMethod>ClassicalRotors</me:DOSCMethod>
</molecule>
<molecule id="INT2" description="Second Intermediate">
<atomArray>

<atom id="a1" elementType="C" x3="-0.155270" y3="-0.276385" z3="1.854336"/>
<atom id="a2" elementType="H" x3="-0.186386" y3="-1.347169" z3="2.057091"/>
<atom id="a3" elementType="H" x3="-0.068875" y3="0.226413" z3="2.817933"/>
<atom id="a4" elementType="C" x3="1.104794" y3="0.022034" z3="1.020196"/>
<atom id="a5" elementType="C" x3="0.855851" y3="0.627981" z3="-0.405350"/>
<atom id="a6" elementType="C" x3="-0.627096" y3="0.843455" z3="-0.671720"/>
<atom id="a7" elementType="H" x3="-0.779816" y3="1.287572" z3="-1.655851"/>
<atom id="a8" elementType="C" x3="-1.547627" y3="-0.359426" z3="-0.284889"/>
<atom id="a9" elementType="C" x3="-1.438981" y3="0.185075" z3="1.167830"/>
<atom id="a10" elementType="H" x3="-2.300851" y3="0.044034" z3="1.820267"/>
<atom id="a11" elementType="C" x3="-1.198287" y3="1.585267" z3="0.566845"/>
<atom id="a12" elementType="H" x3="-0.529046" y3="2.249148" z3="1.113277"/>
<atom id="a13" elementType="H" x3="-2.111925" y3="2.126866" z3="0.347051"/>
<atom id="a14" elementType="C" x3="-1.146882" y3="-1.800843" z3="-0.563263"/>
<atom id="a15" elementType="H" x3="-1.097115" y3="-1.977895" z3="-1.638187"/>
<atom id="a16" elementType="H" x3="-1.907258" y3="-2.468216" z3="-0.150330"/>
<atom id="a17" elementType="C" x3="-2.948664" y3="-0.150929" z3="-0.863033"/>
<atom id="a18" elementType="H" x3="-2.942921" y3="-0.359712" z3="-1.934242"/>
<atom id="a19" elementType="H" x3="-3.333791" y3="0.857894" z3="-0.725302"/>
<atom id="a20" elementType="H" x3="-3.651853" y3="-0.841512" z3="-0.394373"/>
<atom id="a21" elementType="C" x3="1.669540" y3="1.895639" z3="-0.628116"/>
<atom id="a22" elementType="H" x3="2.729911" y3="1.676690" z3="-0.511695"/>
<atom id="a23" elementType="H" x3="1.400599" y3="2.674302" z3="0.084812"/>
<atom id="a24" elementType="H" x3="1.498931" y3="2.274165" z3="-1.635338"/>
<atom id="a25" elementType="H" x3="-0.186941" y3="-2.081125" z3="-0.145258"/>
<atom id="a26" elementType="H" x3="1.784184" y3="0.681924" z3="1.563199"/>
<atom id="a27" elementType="O" x3="1.351553" y3="-0.401895" z3="-1.262847"/>
<atom id="a28" elementType="O" x3="2.406320" y3="-0.954070" z3="-0.501679"/>
<atom id="a29" elementType="O" x3="1.776988" y3="-1.200858" z3="0.743268"/>
</atomArray>
<bondArray>
<bond atomRefs2="a18 a17" order="1"/>
<bond atomRefs2="a7 a6" order="1"/>
<bond atomRefs2="a15 a14" order="1"/>
<bond atomRefs2="a24 a21" order="1"/>
<bond atomRefs2="a27 a28" order="1"/>
<bond atomRefs2="a27 a5" order="1"/>
<bond atomRefs2="a17 a19" order="1"/>
<bond atomRefs2="a17 a20" order="1"/>
<bond atomRefs2="a17 a8" order="1"/>
<bond atomRefs2="a6 a5" order="1"/>
<bond atomRefs2="a6 a8" order="1"/>
<bond atomRefs2="a6 a11" order="1"/>
<bond atomRefs2="a21 a22" order="1"/>
<bond atomRefs2="a21 a5" order="1"/>
<bond atomRefs2="a21 a23" order="1"/>
<bond atomRefs2="a14 a8" order="1"/>
<bond atomRefs2="a14 a16" order="1"/>
<bond atomRefs2="a14 a25" order="1"/>
<bond atomRefs2="a28 a29" order="1"/>

<bond atomRefs2="a5 a4" order="1"/>
<bond atomRefs2="a8 a9" order="1"/>
<bond atomRefs2="a13 a11" order="1"/>
<bond atomRefs2="a11 a12" order="1"/>
<bond atomRefs2="a11 a9" order="1"/>
<bond atomRefs2="a29 a4" order="1"/>
<bond atomRefs2="a4 a26" order="1"/>
<bond atomRefs2="a4 a1" order="1"/>
<bond atomRefs2="a9 a10" order="1"/>
<bond atomRefs2="a9 a1" order="1"/>
<bond atomRefs2="a1 a2" order="1"/>
<bond atomRefs2="a1 a3" order="1"/>
</bondArray>
<propertyList>
<property title="File Format">
<scalar>g09</scalar>
</property>
<property dictRef="me:ZPE">
<scalar units="kcal/mol">-57.53433024</scalar>
</property>
<property dictRef="me:rotConsts">
<array units="cm-1">0.044 0.026 0.024</array>
</property>
<property dictRef="me:symmetryNumber">
<scalar>1</scalar>
</property>
<property dictRef="me:vibFreqs">
<array units="cm-1">123.25 166.92 187.17 201.79 228.22 238.34 277.93 292.24 312.54 356.10 377.65 396.13 417.08 434.84 468.31 500.75 559.86 607.42 630.98 679.28 735.53 773.24 835.76 845.96 872.23 897.87 918.96 937.28 944.94 971.70 981.55 984.56 1000.03 1025.09 1038.46 1048.59 1074.95 1097.10 1120.44 1139.65 1168.03 1176.68 1212.72 1229.70 1249.71 1264.28 1272.33 1291.64 1305.79 1322.99 1356.80 1361.53 1393.04 1402.47 1415.28 1417.79 1437.18 1489.31 1492.78 1496.53 1501.75 1501.82 1513.46 1528.39 1535.55 3043.08 3047.16 3055.81 3059.13 3070.68 3082.22 3084.87 3091.95 3107.44 3112.45 3114.49 3130.56 3138.40 3143.86 3164.57 3185.55</array>
</property>
<property dictRef="me:spinMultiplicity">
<scalar>1</scalar>
</property>
<property dictRef="me:epsilon">
<scalar>600.00</scalar>
</property>
<property dictRef="me:sigma">
<scalar>6.5</scalar>
</property>
</propertyList>
<me:energyTransferModel xsi:type="me:ExponentialDown">
<me:deltaEDown units="cm-1">225.0</me:deltaEDown>
</me:energyTransferModel>
<me:DOSCMethod>ClassicalRotors</me:DOSCMethod>

</molecule>
<molecule id="INT-CI" description="Criegee Intermediate">
<atomArray>
<atom id="a1" elementType="C" x3="0.075094" y3="-1.424705" z3="-0.896822"/>
<atom id="a2" elementType="C" x3="-0.788346" y3="-0.149530" z3="-0.706108"/>
<atom id="a3" elementType="C" x3="1.337736" y3="-0.556560" z3="-0.667341"/>
<atom id="a4" elementType="C" x3="0.435312" y3="0.532678" z3="0.009698"/>
<atom id="a5" elementType="H" x3="-0.015205" y3="-1.943659" z3="-1.856555"/>
<atom id="a6" elementType="H" x3="-0.055420" y3="-2.153495" z3="-0.088308"/>
<atom id="a7" elementType="C" x3="0.665311" y3="1.979180" z3="-0.405368"/>
<atom id="a8" elementType="H" x3="0.740930" y3="2.065406" z3="-1.495706"/>
<atom id="a9" elementType="H" x3="1.590978" y3="2.366024" z3="0.031545"/>
<atom id="a10" elementType="H" x3="-0.172436" y3="2.605810" z3="-0.072651"/>
<atom id="a11" elementType="C" x3="0.374983" y3="0.429306" z3="1.533737"/>
<atom id="a12" elementType="H" x3="-0.433152" y3="1.061332" z3="1.923381"/>
<atom id="a13" elementType="H" x3="1.313822" y3="0.794766" z3="1.968273"/>
<atom id="a14" elementType="H" x3="0.206600" y3="-0.591017" z3="1.896942"/>
<atom id="a15" elementType="C" x3="2.492958" y3="-1.214350" z3="0.067043"/>
<atom id="a16" elementType="H" x3="2.196368" y3="-1.547902" z3="1.072536"/>
<atom id="a17" elementType="H" x3="2.796560" y3="-2.128893" z3="-0.466883"/>
<atom id="a18" elementType="C" x3="3.732069" y3="-0.366567" z3="0.222798"/>
<atom id="a19" elementType="H" x3="4.585894" y3="-0.853185" z3="0.728149"/>
<atom id="a20" elementType="O" x3="3.830996" y3="0.794889" z3="-0.180071"/>
<atom id="a21" elementType="C" spinMultiplicity="2" x3="-2.100287" y3="-0.214621" z3="-0.021806"/>
<atom id="a22" elementType="C" x3="-2.560148" y3="-1.150317" z3="1.020658"/>
<atom id="a23" elementType="H" x3="-1.831645" y3="-1.947667" z3="1.164416"/>
<atom id="a24" elementType="H" x3="-3.534061" y3="-1.561432" z3="0.726070"/>
<atom id="a25" elementType="H" x3="-2.727375" y3="-0.606636" z3="1.959105"/>
<atom id="a26" elementType="O" x3="-2.949525" y3="0.765629" z3="-0.360694"/>
<atom id="a27" elementType="O" spinMultiplicity="2" x3="-4.082903" y3="0.836132" z3="0.244683"/>
<atom id="a28" elementType="H" x3="-0.926463" y3="0.339709" z3="-1.678563"/>
<atom id="a29" elementType="H" x3="1.694394" y3="-0.158414" z3="-1.625738"/>
</atomArray>
<bondArray>
<bond atomRefs2="a5 a1" order="1"/>
<bond atomRefs2="a28 a2" order="1"/>
<bond atomRefs2="a29 a3" order="1"/>
<bond atomRefs2="a8 a7" order="1"/>
<bond atomRefs2="a1 a2" order="1"/>
<bond atomRefs2="a1 a3" order="1"/>
<bond atomRefs2="a1 a6" order="1"/>
<bond atomRefs2="a2 a21" order="1"/>
<bond atomRefs2="a2 a4" order="1"/>
<bond atomRefs2="a3 a4" order="1"/>
<bond atomRefs2="a3 a15" order="1"/>
<bond atomRefs2="a17 a15" order="1"/>
<bond atomRefs2="a7 a10" order="1"/>
<bond atomRefs2="a7 a4" order="1"/>

<bond atomRefs2="a7 a9" order="1"/>
<bond atomRefs2="a26 a21" order="1"/>
<bond atomRefs2="a26 a27" order="1"/>
<bond atomRefs2="a20 a18" order="2"/>
<bond atomRefs2="a21 a22" order="1"/>
<bond atomRefs2="a4 a11" order="1"/>
<bond atomRefs2="a15 a18" order="1"/>
<bond atomRefs2="a15 a16" order="1"/>
<bond atomRefs2="a18 a19" order="1"/>
<bond atomRefs2="a24 a22" order="1"/>
<bond atomRefs2="a22 a23" order="1"/>
<bond atomRefs2="a22 a25" order="1"/>
<bond atomRefs2="a11 a14" order="1"/>
<bond atomRefs2="a11 a12" order="1"/>
<bond atomRefs2="a11 a13" order="1"/>
</bondArray>
<propertyList>
<property title="File Format">
<scalar>g09</scalar>
</property>
<property dictRef="me:ZPE">
<scalar units="kcal/mol">-74.91872203</scalar>
</property>
<property dictRef="me:rotConsts">
<array units="cm-1">0.0596 0.01378987 0.0128585623</array>
</property>
<property dictRef="me:symmetryNumber">
<scalar>1</scalar>
</property>
<property dictRef="me:vibFreqs">
<array units="cm-1">31.14 39.68 73.38 110.86 118.41 153.48 171.06
222.45 259.12 270.31 273.68 283.09 323.73
353.73 356.91 383.11 403.73 463.01 505.27
589.9 619.37 644.91 708.27 751.39 779.67 791.97
840.14 946.62 971.16 991.1 1020.65 1031.39 1043.7
1087.48 1108.32 1122.03 1125.74 1177.26 1182.5
1243.38 1260.96 1295.18 1316.75 1367.99 1371.08
1394.69 1403.57 1424.15 1472.69 1493.04 1521.22
1527.21 1534.2 1549.68 1555.14 1580.99 1583.25
1593.41 1607.69 1610.39 1620.56 1631.89 1633.64
1794.09 1890.23 3115.36 3159.47 3163.74 3169.79
3181.31 3200.62 3207.19 3209.65 3220.7 3229.01
3232.23 3239.61 3243.12 3258.18 3275.4
3308.78</array>
</property>
<property dictRef="me:spinMultiplicity">
<scalar>1</scalar>
</property>
<property dictRef="me:epsilon">
<scalar>600.00</scalar>

</property>
<property dictRef="me:sigma">
<scalar>6.5</scalar>
</property>
</propertyList>
<me:energyTransferModel xsi:type="me:ExponentialDown">
<me:deltaEDown units="cm-1">225.0</me:deltaEDown>
</me:energyTransferModel>
<me:DOSCMethod>ClassicalRotors</me:DOSCMethod>
</molecule>
<molecule id="TS-CI_RB" description="TS to CI RingBreak">
<atomArray>
<atom id="a1" elementType="C" x3="0.042632" y3="-1.329727" z3="-0.813911"/>
<atom id="a2" elementType="C" x3="-1.185314" y3="-0.494824" z3="-1.077338"/>
<atom id="a3" elementType="C" x3="1.271348" y3="-0.409294" z3="-0.576143"/>
<atom id="a4" elementType="C" spinMultiplicity="2" x3="0.911316" y3="0.762752" z3="0.299322"/>
<atom id="a5" elementType="H" x3="0.244495" y3="-1.997241" z3="-1.663734"/>
<atom id="a6" elementType="H" x3="-0.107217" y3="-1.969504" z3="0.061937"/>
<atom id="a7" elementType="C" x3="0.955286" y3="2.160159" z3="-0.225569"/>
<atom id="a8" elementType="H" x3="0.953753" y3="2.175793" z3="-1.321008"/>
<atom id="a9" elementType="H" x3="1.864856" y3="2.690692" z3="0.101492"/>
<atom id="a10" elementType="H" x3="0.091524" y3="2.740836" z3="0.126491"/>
<atom id="a11" elementType="C" x3="0.521864" y3="0.523252" z3="1.724251"/>
<atom id="a12" elementType="H" x3="-0.312234" y3="1.175316" z3="2.016850"/>
<atom id="a13" elementType="H" x3="1.349280" y3="0.735483" z3="2.425164"/>
<atom id="a14" elementType="H" x3="0.208336" y3="-0.513333" z3="1.899800"/>
<atom id="a15" elementType="C" x3="2.433456" y3="-1.218011" z3="0.013775"/>
<atom id="a16" elementType="H" x3="2.173986" y3="-1.604402" z3="1.010861"/>
<atom id="a17" elementType="H" x3="2.666714" y3="-2.105802" z3="-0.595281"/>
<atom id="a18" elementType="C" x3="3.710002" y3="-0.423065" z3="0.160123"/>
<atom id="a19" elementType="H" x3="4.540144" y3="-0.950263" z3="0.677311"/>
<atom id="a20" elementType="O" x3="3.862647" y3="0.713242" z3="-0.246982"/>
<atom id="a21" elementType="C" x3="-2.276411" y3="-0.437698" z3="-0.287289"/>
<atom id="a22" elementType="C" x3="-2.691497" y3="-1.275959" z3="0.874890"/>
<atom id="a23" elementType="H" x3="-1.941359" y3="-2.047203" z3="1.050214"/>
<atom id="a24" elementType="H" x3="-3.652272" y3="-1.754562" z3="0.672913"/>
<atom id="a25" elementType="H" x3="-2.804966" y3="-0.670351" z3="1.776950"/>
<atom id="a26" elementType="O" x3="-3.181877" y3="0.589441" z3="-0.682036"/>
<atom id="a27" elementType="O" spinMultiplicity="2" x3="-4.235394" y3="0.692093" z3="0.053923"/>
<atom id="a28" elementType="H" x3="-1.113956" y3="0.258865" z3="-1.862022"/>
<atom id="a29" elementType="H" x3="1.593899" y3="-0.014773" z3="-1.548536"/>
</atomArray>
<bondArray>
<bond atomRefs2="a28 a2" order="1"/>
<bond atomRefs2="a5 a1" order="1"/>
<bond atomRefs2="a29 a3" order="1"/>
<bond atomRefs2="a8 a7" order="1"/>
<bond atomRefs2="a2 a1" order="1"/>

<bond atomRefs2="a2 a21" order="2"/>
<bond atomRefs2="a1 a3" order="1"/>
<bond atomRefs2="a1 a6" order="1"/>
<bond atomRefs2="a26 a21" order="1"/>
<bond atomRefs2="a26 a27" order="1"/>
<bond atomRefs2="a17 a15" order="1"/>
<bond atomRefs2="a3 a15" order="1"/>
<bond atomRefs2="a3 a4" order="1"/>
<bond atomRefs2="a21 a22" order="1"/>
<bond atomRefs2="a20 a18" order="2"/>
<bond atomRefs2="a7 a9" order="1"/>
<bond atomRefs2="a7 a10" order="1"/>
<bond atomRefs2="a7 a4" order="1"/>
<bond atomRefs2="a15 a18" order="1"/>
<bond atomRefs2="a15 a16" order="1"/>
<bond atomRefs2="a18 a19" order="1"/>
<bond atomRefs2="a4 a11" order="1"/>
<bond atomRefs2="a24 a22" order="1"/>
<bond atomRefs2="a22 a23" order="1"/>
<bond atomRefs2="a22 a25" order="1"/>
<bond atomRefs2="a11 a14" order="1"/>
<bond atomRefs2="a11 a12" order="1"/>
<bond atomRefs2="a11 a13" order="1"/>
</bondArray>
<propertyList>
<property title="File Format">
<scalar>g09</scalar>
</property>
<property dictRef="me:ZPE">
<scalar units="kcal/mol">-30.82</scalar>
</property>
<property dictRef="me:rotConsts">
<array units="cm-1">0.05695707 0.013195128 0.012321858</array>
</property>
<property dictRef="me:symmetryNumber">
<scalar>1</scalar>
</property>
<property dictRef="me:vibFreqs">
<array units="cm-1">45.41 79.3 84.46 112.44 136.76 188.86 236.55
250.55 268.9 276.34 287.98 297.64 313.71 321.48
343.27 383.24 417.6 439.9 482.83 542.42 564.71
660.8 715.89 799.66 842.99 878.97 892.02 979.1
994.55 1032.56 1056.84 1072.37 1087.76 1114.48
1145.19 1160.75 1168.77 1173.26 1190.22 1228.32
1292.68 1326.7 1362.35 1385.16 1410.6 1417.08
1466.7 1505.79 1512.23 1526.78 1546.56 1557.39
1559.79 1575.82 1578.93 1596.41 1598.55 1603.38
1609.21 1615.79 1622.78 1630 1704.03 1898.04 2995.29
3055.36 3080.69 3097.16 3122.74 3124.92 3130.32

3147.28 3148.46 3154.54 3169.74 3182.02 3191.22
3198.36 3242.71 3251.45</array>
</property>
<property dictRef="me:imFreqs">
<array units="cm-1">520.64</array>
</property>
</propertyList>
<me:DOSCMethod>ClassicalRotors</me:DOSCMethod>
</molecule>
<molecule id="TS3" description="TS connecting CI and VHP">
<atomArray>
<atom id="a1" elementType="C" x3="2.896741" y3="-0.300571" z3="0.036853"/>
<atom id="a2" elementType="C" x3="3.206796" y3="-1.638003" z3="0.656633"/>
<atom id="a3" elementType="H" x3="3.097159" y3="-2.437867" z3="-0.077215"/>
<atom id="a4" elementType="H" x3="2.491244" y3="-1.841552" z3="1.456362"/>
<atom id="a5" elementType="H" x3="4.212715" y3="-1.633130" z3="1.066086"/>
<atom id="a6" elementType="O" x3="3.643053" y3="0.642365" z3="0.154912"/>
<atom id="a7" elementType="C" x3="1.605755" y3="-0.141747" z3="-0.729774"/>
<atom id="a8" elementType="H" x3="1.875805" y3="0.204878" z3="-1.729105"/>
<atom id="a9" elementType="C" x3="0.532185" y3="-1.241527" z3="-0.808324"/>
<atom id="a10" elementType="H" x3="0.509685" y3="-1.863872" z3="0.086583"/>
<atom id="a11" elementType="H" x3="0.534383" y3="-1.890683" z3="-1.681840"/>
<atom id="a12" elementType="C" x3="-0.541781" y3="-0.134318" z3="-0.743072"/>
<atom id="a13" elementType="H" x3="-0.763670" y3="0.212190" z3="-1.755324"/>
<atom id="a14" elementType="C" x3="0.529811" y3="0.828626" z3="-0.138301"/>
<atom id="a15" elementType="C" x3="-1.819828" y3="-0.361867" z3="0.008709"/>
<atom id="a16" elementType="C" spinMultiplicity="2" x3="-3.005140" y3="-0.597067" z3="-
0.696302"/>
<atom id="a17" elementType="C" x3="0.535684" y3="2.246744" z3="-0.671452"/>
<atom id="a18" elementType="H" x3="-0.346267" y3="2.790263" z3="-0.325667"/>
<atom id="a19" elementType="H" x3="0.536499" y3="2.257453" z3="-1.762542"/>
<atom id="a20" elementType="H" x3="1.422914" y3="2.779811" z3="-0.327879"/>
<atom id="a21" elementType="C" x3="0.526943" y3="0.830915" z3="1.385710"/>
<atom id="a22" elementType="H" x3="-0.352425" y3="1.353131" z3="1.763783"/>
<atom id="a23" elementType="H" x3="1.411989" y3="1.343621" z3="1.762467"/>
<atom id="a24" elementType="H" x3="0.517910" y3="-0.176733" z3="1.806154"/>
<atom id="a25" elementType="O" x3="-4.114763" y3="-0.345394" z3="-0.093763"/>
<atom id="a26" elementType="O" spinMultiplicity="2" x3="-3.845941" y3="0.111888"
z3="1.184953"/>
<atom id="a27" elementType="H" x3="-1.723807" y3="-0.956035" z3="0.920833"/>
<atom id="a28" elementType="H" x3="-2.558050" y3="0.422539" z3="0.782283"/>
<atom id="a29" elementType="H" x3="-3.127874" y3="-0.781996" z3="-1.757876"/>
</atomArray>
<bondArray>
<bond atomRefs2="a19 a17" order="1"/>
<bond atomRefs2="a29 a16" order="1"/>
<bond atomRefs2="a13 a12" order="1"/>
<bond atomRefs2="a8 a7" order="1"/>
<bond atomRefs2="a11 a9" order="1"/>
<bond atomRefs2="a9 a12" order="1"/>

<bond atomRefs2="a9 a7" order="1"/>
<bond atomRefs2="a9 a10" order="1"/>
<bond atomRefs2="a12 a14" order="1"/>
<bond atomRefs2="a12 a15" order="1"/>
<bond atomRefs2="a7 a14" order="1"/>
<bond atomRefs2="a7 a1" order="1"/>
<bond atomRefs2="a16 a25" order="1"/>
<bond atomRefs2="a16 a15" order="1"/>
<bond atomRefs2="a17 a20" order="1"/>
<bond atomRefs2="a17 a18" order="1"/>
<bond atomRefs2="a17 a14" order="1"/>
<bond atomRefs2="a14 a21" order="1"/>
<bond atomRefs2="a25 a26" order="1"/>
<bond atomRefs2="a3 a2" order="1"/>
<bond atomRefs2="a15 a28" order="1"/>
<bond atomRefs2="a15 a27" order="1"/>
<bond atomRefs2="a1 a6" order="2"/>
<bond atomRefs2="a1 a2" order="1"/>
<bond atomRefs2="a2 a5" order="1"/>
<bond atomRefs2="a2 a4" order="1"/>
<bond atomRefs2="a21 a23" order="1"/>
<bond atomRefs2="a21 a22" order="1"/>
<bond atomRefs2="a21 a24" order="1"/>
</bondArray>
<propertyList>
<property title="File Format">
<scalar>g09</scalar>
</property>
<property dictRef="me:ZPE">
<scalar units="kcal/mol">-58.88277808</scalar>
</property>
<property dictRef="me:rotConsts">
<array units="cm-1">0.056 0.013 0.012</array>
</property>
<property dictRef="me:symmetryNumber">
<scalar>1</scalar>
</property>
<property dictRef="me:vibFreqs">
<array units="cm-1">30.59 39.26 53.60 97.70 145.21 173.42 176.36 227.01 252.45 269.51 293.56 308.42 321.39 355.63 397.30 463.41 492.77 524.96 529.32 571.56 615.14 640.50 724.32 750.05 825.89 876.25 886.91 924.31 942.21 961.40 970.71 973.70 997.19 1011.29 1028.06 1042.85 1103.81 1121.40 1170.21 1182.43 1200.36 1210.27 1233.34 1255.18 1261.37 1280.64 1282.84 1316.79 1330.17 1359.44 1393.35 1395.81 1414.47 1428.39 1450.83 1467.36 1478.41 1491.06 1498.64 1504.68 1517.35 1519.75 1596.68 1809.09 1867.48 3036.70 3044.88 3050.84 3054.03 3074.75 3076.87 3078.99 3108.85 3113.71 3116.69 3121.70 3129.05 3130.72 3170.70 3191.50</array>
</property>
<property dictRef="me:imFreqs">
<array units="cm-1">1572.25</array>
</property>

</propertyList>
<me:DOSCMethod>ClassicalRotors</me:DOSCMethod>
</molecule>
<molecule id="TS_vin" description="TS connnecting VHP and Vinoxy">
<atomArray>
<atom id="a1" elementType="C" x3="-2.479634" y3="-0.508384" z3="-0.257487"/>
<atom id="a2" elementType="C" x3="-3.637671" y3="0.244479" z3="-0.870815"/>
<atom id="a3" elementType="H" x3="-3.328611" y3="1.239177" z3="-1.195756"/>
<atom id="a4" elementType="H" x3="-4.413360" y3="0.386690" z3="-0.113802"/>
<atom id="a5" elementType="H" x3="-4.050331" y3="-0.313057" z3="-1.707422"/>
<atom id="a6" elementType="O" x3="-2.229517" y3="-1.652632" z3="-0.576718"/>
<atom id="a7" elementType="C" x3="-1.646801" y3="0.241529" z3="0.759974"/>
<atom id="a8" elementType="H" x3="-2.277290" y3="0.971993" z3="1.267622"/>
<atom id="a9" elementType="C" x3="-0.777853" y3="-0.647135" z3="1.674484"/>
<atom id="a10" elementType="H" x3="-1.025738" y3="-1.705139" z3="1.640893"/>
<atom id="a11" elementType="H" x3="-0.773053" y3="-0.313345" z3="2.709694"/>
<atom id="a12" elementType="C" x3="0.518238" y3="-0.247811" z3="0.916342"/>
<atom id="a13" elementType="H" x3="1.311859" y3="0.180165" z3="1.523363"/>
<atom id="a14" elementType="C" x3="-0.321565" y3="0.893540" z3="0.173072"/>
<atom id="a15" elementType="C" spinMultiplicity="2" x3="1.083062" y3="-1.287412" z3="0.041161"/>
<atom id="a16" elementType="H" x3="0.408791" y3="-2.014686" z3="-0.397788"/>
<atom id="a17" elementType="C" x3="2.427303" y3="-1.375357" z3="-0.275471"/>
<atom id="a18" elementType="H" x3="2.751610" y3="-2.114799" z3="-1.013742"/>
<atom id="a19" elementType="C" x3="-0.045804" y3="2.243698" z3="0.827424"/>
<atom id="a20" elementType="H" x3="0.964197" y3="2.578781" z3="0.587119"/>
<atom id="a21" elementType="H" x3="-0.137435" y3="2.194919" z3="1.913413"/>
<atom id="a22" elementType="H" x3="-0.750391" y3="2.995303" z3="0.463659"/>
<atom id="a23" elementType="C" x3="-0.164557" y3="1.000915" z3="-1.337514"/>
<atom id="a24" elementType="H" x3="0.863456" y3="1.271062" z3="-1.582267"/>
<atom id="a25" elementType="H" x3="-0.817130" y3="1.780196" z3="-1.737444"/>
<atom id="a26" elementType="H" x3="-0.395658" y3="0.069305" z3="-1.852612"/>
<atom id="a27" elementType="O" x3="3.306255" y3="-0.622400" z3="0.264132"/>
<atom id="a28" elementType="O" spinMultiplicity="2" x3="3.596791" y3="0.856110" z3="-0.895216"/>
<atom id="a29" elementType="H" x3="4.552547" y3="0.796443" z3="-0.749535"/>
</atomArray>
<bondArray>
<bond atomRefs2="a26 a23" order="1"/>
<bond atomRefs2="a25 a23" order="1"/>
<bond atomRefs2="a5 a2" order="1"/>
<bond atomRefs2="a24 a23" order="1"/>
<bond atomRefs2="a23 a14" order="1"/>
<bond atomRefs2="a3 a2" order="1"/>
<bond atomRefs2="a18 a17" order="1"/>
<bond atomRefs2="a28 a29" order="1"/>
<bond atomRefs2="a2 a1" order="1"/>
<bond atomRefs2="a2 a4" order="1"/>
<bond atomRefs2="a6 a1" order="2"/>
<bond atomRefs2="a16 a15" order="1"/>

<bond atomRefs2="a17 a15" order="1"/>
<bond atomRefs2="a17 a27" order="2"/>
<bond atomRefs2="a1 a7" order="1"/>
<bond atomRefs2="a15 a12" order="1"/>
<bond atomRefs2="a14 a7" order="1"/>
<bond atomRefs2="a14 a19" order="1"/>
<bond atomRefs2="a14 a12" order="1"/>
<bond atomRefs2="a22 a19" order="1"/>
<bond atomRefs2="a20 a19" order="1"/>
<bond atomRefs2="a7 a8" order="1"/>
<bond atomRefs2="a7 a9" order="1"/>
<bond atomRefs2="a19 a21" order="1"/>
<bond atomRefs2="a12 a13" order="1"/>
<bond atomRefs2="a12 a9" order="1"/>
<bond atomRefs2="a10 a9" order="1"/>
<bond atomRefs2="a9 a11" order="1"/>
</bondArray>
<propertyList>
<property title="File Format">
<scalar>g09</scalar>
</property>
<property dictRef="me:ZPE">
<scalar units="kcal/mol">-90.57</scalar>
</property>
<property dictRef="me:rotConsts">
<array units="cm-1">0.044 0.016 0.015</array>
</property>
<property dictRef="me:symmetryNumber">
<scalar>1</scalar>
</property>
<property dictRef="me:vibFreqs">
<array units="cm-1">26.76 33.44 49.60 62.62 117.80 126.34 153.86 162.23 169.68 221.70 252.87 260.35 287.04 296.26 318.25 355.06 380.60 391.18 423.10 495.42 575.26 590.88 629.96 683.89 748.78 776.34 795.57 829.59 866.65 895.60 936.31 954.97 964.45 986.39 995.53 1019.54 1036.57 1040.63 1076.33 1138.59 1143.43 1168.00 1188.10 1211.68 1227.76 1250.82 1266.85 1276.08 1284.68 1327.87 1368.14 1387.97 1406.58 1425.97 1441.55 1462.82 1475.10 1475.78 1486.47 1493.91 1506.75 1512.21 1515.55 1759.91 3016.83 3025.35 3028.05 3036.98 3054.27 3071.64 3077.23 3082.18 3085.25 3092.06 3096.44 3102.21 3124.12 3137.37 3158.55 3767.03</array>
</property>
<property dictRef="me:imFreqs">
<array units="cm-1">379.02</array>
</property>
</propertyList>
<me:DOSCMethod>ClassicalRotors</me:DOSCMethod>
</molecule>
<molecule id="INT-VHP" description="VHP Intermediate">
<atomArray>
<atom id="a1" elementType="C" x3="2.912674" y3="-0.326105" z3="-0.215275"/>
<atom id="a2" elementType="C" x3="3.809206" y3="0.886825" z3="-0.265616"/>

<atom id="a3" elementType="H" x3="3.291751" y3="1.756970" z3="0.141842"/>
<atom id="a4" elementType="H" x3="4.047607" y3="1.118005" z3="-1.305667"/>
<atom id="a5" elementType="H" x3="4.726723" y3="0.703073" z3="0.286203"/>
<atom id="a6" elementType="O" x3="3.282797" y3="-1.371914" z3="0.261253"/>
<atom id="a7" elementType="C" x3="1.524291" y3="-0.140267" z3="-0.766250"/>
<atom id="a8" elementType="H" x3="1.585085" y3="0.395569" z3="-1.715942"/>
<atom id="a9" elementType="C" x3="0.582135" y3="-1.346102" z3="-0.844544"/>
<atom id="a10" elementType="H" x3="0.848501" y3="-2.089094" z3="-0.093582"/>
<atom id="a11" elementType="H" x3="0.479536" y3="-1.840260" z3="-1.807786"/>
<atom id="a12" elementType="C" x3="-0.577606" y3="-0.439355" z3="-0.386913"/>
<atom id="a13" elementType="H" x3="-1.064582" y3="0.012791" z3="-1.252278"/>
<atom id="a14" elementType="C" x3="0.477285" y3="0.583183" z3="0.158792"/>
<atom id="a15" elementType="C" x3="-1.606592" y3="-0.990328" z3="0.533977"/>
<atom id="a16" elementType="H" x3="-1.300235" y3="-1.737538" z3="1.255297"/>
<atom id="a17" elementType="C" x3="-2.874752" y3="-0.608376" z3="0.587328"/>
<atom id="a18" elementType="H" x3="-3.610975" y3="-1.017275" z3="1.265712"/>
<atom id="a19" elementType="C" x3="0.186367" y3="2.038403" z3="-0.150399"/>
<atom id="a20" elementType="H" x3="-0.699088" y3="2.370285" z3="0.395152"/>
<atom id="a21" elementType="H" x3="-0.000358" y3="2.186788" z3="-1.214841"/>
<atom id="a22" elementType="H" x3="1.019012" y3="2.682008" z3="0.142453"/>
<atom id="a23" elementType="C" x3="0.769196" y3="0.392219" z3="1.640995"/>
<atom id="a24" elementType="H" x3="-0.096873" y3="0.689984" z3="2.233134"/>
<atom id="a25" elementType="H" x3="1.614899" y3="1.008615" z3="1.951869"/>
<atom id="a26" elementType="H" x3="1.007897" y3="-0.642861" z3="1.885293"/>
<atom id="a27" elementType="O" x3="-3.336085" y3="0.415538" z3="-0.194594"/>
<atom id="a28" elementType="O" x3="-4.735213" y3="0.240353" z3="-0.397862"/>
<atom id="a29" elementType="H" x3="-4.754113" y3="-0.169465" z3="-1.269805"/>
</atomArray>
<bondArray>
<bond atomRefs2="a11 a9" order="1"/>
<bond atomRefs2="a8 a7" order="1"/>
<bond atomRefs2="a4 a2" order="1"/>
<bond atomRefs2="a29 a28" order="1"/>
<bond atomRefs2="a13 a12" order="1"/>
<bond atomRefs2="a21 a19" order="1"/>
<bond atomRefs2="a9 a7" order="1"/>
<bond atomRefs2="a9 a12" order="1"/>
<bond atomRefs2="a9 a10" order="1"/>
<bond atomRefs2="a7 a1" order="1"/>
<bond atomRefs2="a7 a14" order="1"/>
<bond atomRefs2="a28 a27" order="1"/>
<bond atomRefs2="a12 a14" order="1"/>
<bond atomRefs2="a12 a15" order="1"/>
<bond atomRefs2="a2 a1" order="1"/>
<bond atomRefs2="a2 a3" order="1"/>
<bond atomRefs2="a2 a5" order="1"/>
<bond atomRefs2="a1 a6" order="2"/>
<bond atomRefs2="a27 a17" order="1"/>
<bond atomRefs2="a19 a22" order="1"/>
<bond atomRefs2="a19 a14" order="1"/>

<bond atomRefs2="a19 a20" order="1"/>
<bond atomRefs2="a14 a23" order="1"/>
<bond atomRefs2="a15 a17" order="2"/>
<bond atomRefs2="a15 a16" order="1"/>
<bond atomRefs2="a17 a18" order="1"/>
<bond atomRefs2="a23 a26" order="1"/>
<bond atomRefs2="a23 a25" order="1"/>
<bond atomRefs2="a23 a24" order="1"/>
</bondArray>
<propertyList>
<property title="File Format">
<scalar>g09</scalar>
</property>
<property dictRef="me:ZPE">
<scalar units="kcal/mol">-96.73336358</scalar>
</property>
<property dictRef="me:rotConsts">
<array units="cm-1">0.063 0.012 0.012</array>
</property>
<property dictRef="me:symmetryNumber">
<scalar>1</scalar>
</property>
<property dictRef="me:vibFreqs">
<array units="cm-1">21.91 47.23 64.11 73.31 111.44 138.06 147.23 178.82 215.81 233.57 267.19 276.70 295.50 311.60 338.51 378.02 403.50 426.24 476.07 530.45 587.25 613.78 630.84 686.55 735.28 794.39 810.79 911.20 932.99 955.99 969.35 972.51 976.80 997.08 1005.01 1016.52 1045.17 1073.43 1112.73 1123.34 1167.98 1183.92 1198.90 1210.71 1235.05 1264.89 1270.17 1282.40 1320.12 1353.10 1382.53 1402.60 1410.70 1415.74 1432.10 1438.13 1468.14 1481.22 1489.58 1496.05 1505.12 1513.55 1514.13 1738.09 1817.14 3034.69 3045.42 3051.87 3068.48 3074.78 3085.83 3105.64 3113.77 3114.50 3118.77 3127.95 3143.67 3168.90 3186.10 3217.49 3816.57</array>
</property>
<property dictRef="me:spinMultiplicity">
<scalar>1</scalar>
</property>
<property dictRef="me:epsilon">
<scalar>600.00</scalar>
</property>
<property dictRef="me:sigma">
<scalar>6.5</scalar>
</property>
</propertyList>
<me:energyTransferModel xsi:type="me:ExponentialDown">
<me:deltaEDown units="cm-1">225.0</me:deltaEDown>
</me:energyTransferModel>
<me:DOSCMethod>ClassicalRotors</me:DOSCMethod>
</molecule>
<molecule id="INT-VinoxyOH" description="VHP Intermediate">
<atomArray>
<atom id="a1" elementType="C" x3="2.732074" y3="-0.047123" z3="-0.273758"/>

<atom id="a2" elementType="C" x3="3.507706" y3="1.140760" z3="0.248553"/>
<atom id="a3" elementType="H" x3="2.916270" y3="1.692458" z3="0.988717"/>
<atom id="a4" elementType="H" x3="3.712551" y3="1.834855" z3="-0.575827"/>
<atom id="a5" elementType="H" x3="4.451394" y3="0.814593" z3="0.689507"/>
<atom id="a6" elementType="O" x3="3.219645" y3="-1.159429" z3="-0.327306"/>
<atom id="a7" elementType="C" x3="1.305342" y3="0.219002" z3="-0.696619"/>
<atom id="a8" elementType="H" x3="1.257405" y3="1.165328" z3="-1.249078"/>
<atom id="a9" elementType="C" x3="0.522087" y3="-0.897585" z3="-1.401062"/>
<atom id="a10" elementType="H" x3="0.915111" y3="-1.880721" z3="-1.123733"/>
<atom id="a11" elementType="H" x3="0.439404" y3="-0.839108" z3="-2.488812"/>
<atom id="a12" elementType="C" x3="-0.732787" y3="-0.506147" z3="-0.591411"/>
<atom id="a13" elementType="H" x3="-1.312760" y3="0.261730" z3="-1.113666"/>
<atom id="a14" elementType="C" x3="0.218450" y3="0.238419" z3="0.441784"/>
<atom id="a15" elementType="C" spinMultiplicity="2" x3="-1.625945" y3="-1.553278" z3="-
0.071477"/>
<atom id="a16" elementType="H" x3="-1.237705" y3="-2.560072" z3="0.075056"/>
<atom id="a17" elementType="C" x3="-2.974331" y3="-1.309563" z3="0.333097"/>
<atom id="a18" elementType="H" x3="-3.540415" y3="-2.168610" z3="0.729873"/>
<atom id="a19" elementType="C" x3="-0.272896" y3="1.602838" z3="0.896879"/>
<atom id="a20" elementType="H" x3="-1.163019" y3="1.500107" z3="1.528956"/>
<atom id="a21" elementType="H" x3="-0.535730" y3="2.240876" z3="0.045736"/>
<atom id="a22" elementType="H" x3="0.495247" y3="2.118543" z3="1.487211"/>
<atom id="a23" elementType="C" x3="0.592262" y3="-0.636413" z3="1.637810"/>
<atom id="a24" elementType="H" x3="-0.276009" y3="-0.774737" z3="2.292463"/>
<atom id="a25" elementType="H" x3="1.380350" y3="-0.158291" z3="2.231744"/>
<atom id="a26" elementType="H" x3="0.960562" y3="-1.622963" z3="1.338334"/>
<atom id="a27" elementType="O" x3="-3.532239" y3="-0.203938" z3="0.273484"/>
<atom id="a28" elementType="O" spinMultiplicity="2" x3="-2.808390" y3="2.289803" z3="-
0.888842"/>
<atom id="a29" elementType="H" x3="-3.126549" y3="1.459064" z3="-0.457948"/>
</atomArray>
<bondArray>
<bond atomRefs2="a11 a9" order="1"/>
<bond atomRefs2="a9 a10" order="1"/>
<bond atomRefs2="a9 a7" order="1"/>
<bond atomRefs2="a9 a12" order="1"/>
<bond atomRefs2="a8 a7" order="1"/>
<bond atomRefs2="a13 a12" order="1"/>
<bond atomRefs2="a28 a29" order="1"/>
<bond atomRefs2="a7 a1" order="1"/>
<bond atomRefs2="a7 a14" order="1"/>
<bond atomRefs2="a12 a15" order="1"/>
<bond atomRefs2="a12 a14" order="1"/>
<bond atomRefs2="a4 a2" order="1"/>
<bond atomRefs2="a6 a1" order="2"/>
<bond atomRefs2="a1 a2" order="1"/>
<bond atomRefs2="a15 a16" order="1"/>
<bond atomRefs2="a15 a17" order="1"/>
<bond atomRefs2="a21 a19" order="1"/>
<bond atomRefs2="a2 a5" order="1"/>

<bond atomRefs2="a2 a3" order="1"/>
<bond atomRefs2="a27 a17" order="2"/>
<bond atomRefs2="a17 a18" order="1"/>
<bond atomRefs2="a14 a19" order="1"/>
<bond atomRefs2="a14 a23" order="1"/>
<bond atomRefs2="a19 a22" order="1"/>
<bond atomRefs2="a19 a20" order="1"/>
<bond atomRefs2="a26 a23" order="1"/>
<bond atomRefs2="a23 a25" order="1"/>
<bond atomRefs2="a23 a24" order="1"/>
</bondArray>
<propertyList>
<property title="File Format">
<scalar>g09</scalar>
</property>
<property dictRef="me:ZPE">
<scalar units="kcal/mol">-97.19</scalar>
</property>
<property dictRef="me:rotConsts">
<array units="cm-1">0.043 0.015 0.014</array>
</property>
<property dictRef="me:symmetryNumber">
<scalar>1</scalar>
</property>
<property dictRef="me:vibFreqs">
<array units="cm-1">37.65 44.40 52.74 62.79 81.79 105.18 139.10 150.69 195.83 212.30 241.58 250.51 278.92 302.01 319.72 333.33 376.71 387.87 447.06 466.18 531.02 574.60 586.81 612.22 624.42 681.75 725.02 742.45 808.64 912.39 929.84 968.12 976.46 983.40 991.10 1010.21 1018.08 1045.72 1063.21 1113.50 1144.37 1166.14 1194.79 1206.54 1220.61 1249.65 1268.43 1279.06 1333.53 1357.77 1403.85 1425.90 1434.40 1439.54 1455.56 1491.21 1499.79 1503.17 1512.99 1520.26 1525.50 1537.43 1537.99 1635.57 1832.19 3037.33 3052.32 3062.53 3065.93 3087.45 3103.90 3109.07 3125.05 3132.04 3134.85 3136.20 3149.08 3166.48 3188.89 3199.28 3554.97</array>
</property>
<property dictRef="me:spinMultiplicity">
<scalar>1</scalar>
</property>
<property dictRef="me:epsilon">
<scalar>600.00</scalar>
</property>
<property dictRef="me:sigma">
<scalar>6.5</scalar>
</property>
</propertyList>
<me:energyTransferModel xsi:type="me:ExponentialDown">
<me:deltaEDown units="cm-1">225.0</me:deltaEDown>
</me:energyTransferModel>
<me:DOSCMethod>ClassicalRotors</me:DOSCMethod>
</molecule>
<molecule id="Vinoxy" description="Prod1">

<atomArray>
<atom id="a1" elementType="C" x3="2.448034" y3="-0.315061" z3="-0.051672"/>
<atom id="a2" elementType="C" x3="3.336386" y3="0.903583" z3="-0.071228"/>
<atom id="a3" elementType="H" x3="2.771335" y3="1.789811" z3="0.222639"/>
<atom id="a4" elementType="H" x3="3.689044" y3="1.076446" z3="-1.089981"/>
<atom id="a5" elementType="H" x3="4.187304" y3="0.763477" z3="0.589286"/>
<atom id="a6" elementType="O" x3="2.772047" y3="-1.333386" z3="0.509746"/>
<atom id="a7" elementType="C" x3="1.120243" y3="-0.173024" z3="-0.749331"/>
<atom id="a8" elementType="H" x3="1.269172" y3="0.330856" z3="-1.706315"/>
<atom id="a9" elementType="C" x3="0.214604" y3="-1.401057" z3="-0.880025"/>
<atom id="a10" elementType="H" x3="0.424289" y3="-2.117454" z3="-0.086448"/>
<atom id="a11" elementType="H" x3="0.214846" y3="-1.920172" z3="-1.835181"/>
<atom id="a12" elementType="C" x3="-1.006401" y3="-0.516949" z3="-0.567594"/>
<atom id="a13" elementType="H" x3="-1.435339" y3="-0.086277" z3="-1.473114"/>
<atom id="a14" elementType="C" x3="-0.021154" y3="0.564537" z3="0.039644"/>
<atom id="a15" elementType="C" spinMultiplicity="2" x3="-2.084943" y3="-1.027265" z3="0.285908"/>
<atom id="a16" elementType="H" x3="-1.897958" y3="-1.872267" z3="0.938878"/>
<atom id="a17" elementType="C" x3="-3.364696" y3="-0.400562" z3="0.352318"/>
<atom id="a18" elementType="H" x3="-4.100896" y3="-0.855161" z3="1.036110"/>
<atom id="a19" elementType="C" x3="-0.317444" y3="1.993261" z3="-0.365433"/>
<atom id="a20" elementType="H" x3="-1.266003" y3="2.319275" z3="0.063196"/>
<atom id="a21" elementType="H" x3="-0.391320" y3="2.089921" z3="-1.448923"/>
<atom id="a22" elementType="H" x3="0.465282" y3="2.669297" z3="-0.013444"/>
<atom id="a23" elementType="C" x3="0.126124" y3="0.442211" z3="1.550197"/>
<atom id="a24" elementType="H" x3="-0.792149" y3="0.762698" z3="2.043952"/>
<atom id="a25" elementType="H" x3="0.934837" y3="1.081052" z3="1.909846"/>
<atom id="a26" elementType="H" x3="0.347672" y3="-0.577930" z3="1.863613"/>
<atom id="a27" elementType="O" x3="-3.662626" y3="0.599434" z3="-0.293597"/>
</atomArray>
<bondArray>
<bond atomRefs2="a11 a9" order="1"/>
<bond atomRefs2="a8 a7" order="1"/>
<bond atomRefs2="a13 a12" order="1"/>
<bond atomRefs2="a21 a19" order="1"/>
<bond atomRefs2="a4 a2" order="1"/>
<bond atomRefs2="a9 a7" order="1"/>
<bond atomRefs2="a9 a12" order="1"/>
<bond atomRefs2="a9 a10" order="1"/>
<bond atomRefs2="a7 a1" order="1"/>
<bond atomRefs2="a7 a14" order="1"/>
<bond atomRefs2="a12 a14" order="1"/>
<bond atomRefs2="a12 a15" order="1"/>
<bond atomRefs2="a19 a22" order="1"/>
<bond atomRefs2="a19 a14" order="1"/>
<bond atomRefs2="a19 a20" order="1"/>
<bond atomRefs2="a27 a17" order="2"/>
<bond atomRefs2="a2 a1" order="1"/>
<bond atomRefs2="a2 a3" order="1"/>
<bond atomRefs2="a2 a5" order="1"/>

<bond atomRefs2="a1 a6" order="2"/>
<bond atomRefs2="a14 a23" order="1"/>
<bond atomRefs2="a15 a17" order="1"/>
<bond atomRefs2="a15 a16" order="1"/>
<bond atomRefs2="a17 a18" order="1"/>
<bond atomRefs2="a23 a26" order="1"/>
<bond atomRefs2="a23 a25" order="1"/>
<bond atomRefs2="a23 a24" order="1"/>
</bondArray>
<propertyList>
<property title="File Format">
<scalar>g09</scalar>
</property>
<property dictRef="me:ZPE">
<scalar units="kcal/mol">-81.06658826</scalar>
</property>
<property dictRef="me:rotConsts">
<array units="cm-1">0.065 0.018 0.017</array>
</property>
<property dictRef="me:symmetryNumber">
<scalar>1</scalar>
</property>
<property dictRef="me:vibFreqs">
<array units="cm-1">24.50 57.04 62.78 109.12 142.16 156.58 196.18 224.49 239.73 275.85 296.92 317.63 332.80 371.97 385.01 447.52 467.24 571.50 585.09 623.06 667.66 723.34 735.16 803.35 908.53 921.84 957.00 970.03 979.48 983.99 1004.01 1013.68 1036.43 1051.83 1107.48 1134.14 1154.92 1185.87 1193.70 1210.05 1238.16 1259.48 1267.15 1313.29 1336.88 1382.35 1403.43 1417.37 1420.44 1433.97 1467.21 1471.55 1480.83 1488.62 1495.32 1503.61 1512.32 1513.78 1624.92 1819.43 2965.11 3038.06 3045.30 3052.00 3074.18 3084.13 3088.99 3109.86 3113.79 3118.53 3124.25 3128.41 3148.26 3169.68 3178.36</array>
</property>
<property dictRef="me:spinMultiplicity">
<scalar>2</scalar>
</property>
<property dictRef="me:epsilon">
<scalar>600.00</scalar>
</property>
<property dictRef="me:sigma">
<scalar>6.5</scalar>
</property>
<property dictRef="me:MW">
<scalar units="amu">167</scalar>
</property>
</propertyList>
<me:energyTransferModel xsi:type="me:ExponentialDown">
<me:deltaEDown units="cm-1">225.0</me:deltaEDown>
</me:energyTransferModel>
<me:DOSCMethod>ClassicalRotors</me:DOSCMethod>
</molecule>

<molecule id="OH" description="Prod2">
<atomArray>
<atom id="a1" elementType="H" x3="0.000000" y3="0.000000" z3="-0.862362"/>
<atom id="a2" elementType="O" spinMultiplicity="2" x3="0.000000" y3="0.000000" z3="0.107795"/>
</atomArray>
<bondArray>
<bond atomRefs2="a1 a2" order="1"/>
</bondArray>
<propertyList>
<property title="File Format">
<scalar>g09</scalar>
</property>
<property dictRef="me:ZPE">
<scalar units="kcal/mol">0.0</scalar>
</property>
<property dictRef="me:rotConsts">
<array units="cm-1">18.878</array>
</property>
<property dictRef="me:symmetryNumber">
<scalar>1</scalar>
</property>
<property dictRef="me:vibFreqs">
<array units="cm-1">3776.50</array>
</property>
<property dictRef="me:spinMultiplicity">
<scalar>2</scalar>
</property>
</propertyList>
<me:DOSCMethod>ClassicalRotors</me:DOSCMethod>
</molecule>
<molecule id="O2" description="Reac3">
<atomArray>
<atom id="a1" elementType="O" x3="0.000000" y3="0.000000" z3="0.597938"/>
<atom id="a2" elementType="O" x3="0.000000" y3="0.000000" z3="-0.597938"/>
</atomArray>
<bondArray>
<bond atomRefs2="a2 a1" order="2"/>
</bondArray>
<propertyList>
<property title="File Format">
<scalar>g09</scalar>
</property>
<property dictRef="me:ZPE">
<scalar units="kcal/mol">0.0</scalar>
</property>
<property dictRef="me:spinMultiplicity">
<scalar>3.0</scalar>
</property>
<property title="Vibrational Frequencies" dictRef="me:vibFreqs">

<array units="cm-1">1704.47</array>
</property>
<property title="Rotational Constants" dictRef="me:rotConsts">
<array units="cm-1">1.473</array>
</property>
<property dictRef="me:MW">
<scalar units="amu">32</scalar>
</property>
</propertyList>
<me:DOSCMethod>ClassicalRotors</me:DOSCMethod>
</molecule>
<molecule id="Vinoxy-RB-prod1" description="Ring-broken product">
<atomArray>
<atom id="a1" elementType="C" x3="2.320173" y3="-0.556126" z3="-0.342634"/>
<atom id="a2" elementType="C" x3="3.139409" y3="0.172482" z3="-1.376720"/>
<atom id="a3" elementType="H" x3="2.853306" y3="1.222859" z3="-1.431794"/>
<atom id="a4" elementType="H" x3="2.940075" y3="-0.267239" z3="-2.356585"/>
<atom id="a5" elementType="H" x3="4.198135" y3="0.081211" z3="-1.151186"/>
<atom id="a6" elementType="O" x3="2.819427" y3="-1.293665" z3="0.472174"/>
<atom id="a7" elementType="C" x3="0.813980" y3="-0.267063" z3="-0.345450"/>
<atom id="a8" elementType="H" x3="0.509195" y3="-0.082941" z3="-1.378867"/>
<atom id="a9" elementType="C" x3="0.037062" y3="-1.455014" z3="0.216463"/>
<atom id="a10" elementType="H" x3="0.368678" y3="-1.662445" z3="1.236247"/>
<atom id="a11" elementType="H" x3="0.302299" y3="-2.358327" z3="-0.341072"/>
<atom id="a12" elementType="C" x3="-1.447371" y3="-1.323712" z3="0.209949"/>
<atom id="a13" elementType="C" spinMultiplicity="2" x3="0.660021" y3="1.004811" z3="0.439438"/>
<atom id="a14" elementType="C" x3="-2.173558" y3="-0.307777" z3="-0.252475"/>
<atom id="a15" elementType="H" x3="-1.734295" y3="0.579657" z3="-0.689698"/>
<atom id="a16" elementType="C" x3="-3.637137" y3="-0.347110" z3="-0.182167"/>
<atom id="a17" elementType="H" x3="-4.057778" y3="-1.267590" z3="0.272808"/>
<atom id="a18" elementType="C" x3="0.491343" y3="2.316098" z3="-0.242694"/>
<atom id="a19" elementType="H" x3="-0.388600" y3="2.846529" z3="0.137685"/>
<atom id="a20" elementType="H" x3="0.374306" y3="2.209467" z3="-1.321404"/>
<atom id="a21" elementType="H" x3="1.344613" y3="2.981799" z3="-0.063622"/>
<atom id="a22" elementType="C" x3="0.709383" y3="0.956771" z3="1.926193"/>
<atom id="a23" elementType="H" x3="-0.258526" y3="0.658972" z3="2.349853"/>
<atom id="a24" elementType="H" x3="0.954029" y3="1.933718" z3="2.343423"/>
<atom id="a25" elementType="H" x3="1.448572" y3="0.237089" z3="2.286514"/>
<atom id="a26" elementType="O" x3="-4.363248" y3="0.530910" z3="-0.575202"/>
<atom id="a27" elementType="H" x3="-1.983276" y3="-2.170878" z3="0.632500"/>
</atomArray>
<bondArray>
<bond atomRefs2="a4 a2" order="1"/>
<bond atomRefs2="a3 a2" order="1"/>
<bond atomRefs2="a8 a7" order="1"/>
<bond atomRefs2="a2 a5" order="1"/>
<bond atomRefs2="a2 a1" order="1"/>
<bond atomRefs2="a20 a18" order="1"/>
<bond atomRefs2="a15 a14" order="1"/>

<bond atomRefs2="a26 a16" order="2"/>
<bond atomRefs2="a7 a1" order="1"/>
<bond atomRefs2="a7 a9" order="1"/>
<bond atomRefs2="a7 a13" order="1"/>
<bond atomRefs2="a1 a6" order="2"/>
<bond atomRefs2="a11 a9" order="1"/>
<bond atomRefs2="a14 a16" order="1"/>
<bond atomRefs2="a14 a12" order="2"/>
<bond atomRefs2="a18 a21" order="1"/>
<bond atomRefs2="a18 a19" order="1"/>
<bond atomRefs2="a18 a13" order="1"/>
<bond atomRefs2="a16 a17" order="1"/>
<bond atomRefs2="a12 a9" order="1"/>
<bond atomRefs2="a12 a27" order="1"/>
<bond atomRefs2="a9 a10" order="1"/>
<bond atomRefs2="a13 a22" order="1"/>
<bond atomRefs2="a22 a25" order="1"/>
<bond atomRefs2="a22 a24" order="1"/>
<bond atomRefs2="a22 a23" order="1"/>
</bondArray>
<propertyList>
<property title="File Format">
<scalar>g09</scalar>
</property>
<property dictRef="me:ZPE">
<scalar units="kcal/mol">-78.32172657</scalar>
</property>
<property dictRef="me:rotConsts">
<array units="cm-1">0.051 0.016 0.015</array>
</property>
<property dictRef="me:symmetryNumber">
<scalar>1</scalar>
</property>
<property dictRef="me:vibFreqs">
<array units="cm-1">39.13 46.42 70.85 85.50 86.51 108.10 121.44 132.74 140.67 151.04
192.47 250.33 269.55 315.36 332.07 351.83 379.85 467.74 537.51 584.34 614.52 627.93
723.04 803.86 875.99 930.86 954.82 959.07 993.72 1003.91 1011.46 1024.39 1031.79
1049.94 1057.12 1117.01 1149.47 1183.69 1191.93 1255.85 1270.14 1287.05 1333.19
1342.56 1366.88 1396.06 1408.98 1412.42 1420.86 1430.32 1463.86 1467.54 1475.08
1482.75 1484.57 1497.88 1500.41 1726.04 1808.55 1811.37 2897.26 2991.04 2995.90
3036.32 3048.69 3051.86 3056.02 3064.64 3089.62 3110.26 3116.30 3122.44 3138.01
3169.67 3201.28</array>
</property>
<property dictRef="me:spinMultiplicity">
<scalar>2.0</scalar>
</property>
<!-- <property dictRef="me:epsilon">
<scalar>600.00</scalar>
</property>
<property dictRef="me:sigma">

<scalar>6.5</scalar>
</property> -->
<property dictRef="me:MW">
<scalar units="amu">167</scalar>
</property>
</propertyList>
<me:energyTransferModel xsi:type="me:ExponentialDown">
<me:deltaEDown units="cm-1">225.0</me:deltaEDown>
</me:energyTransferModel>
<me:DOSCMethod>ClassicalRotors</me:DOSCMethod>
</molecule>
<molecule id="Vinoxy-RB-prod2" description="Ring-broken product">
<atomArray>
<atom id="a1" elementType="C" x3="-2.058949" y3="-0.823607" z3="-0.162559"/>
<atom id="a2" elementType="C" x3="-3.532518" y3="-0.936414" z3="0.133748"/>
<atom id="a3" elementType="H" x3="-4.051333" y3="-0.119155" z3="-0.372755"/>
<atom id="a4" elementType="H" x3="-3.726001" y3="-0.829775" z3="1.201107"/>
<atom id="a5" elementType="H" x3="-3.917461" y3="-1.885608" z3="-0.227589"/>
<atom id="a6" elementType="O" x3="-1.498249" y3="-1.589900" z3="-0.904325"/>
<atom id="a7" elementType="C" x3="-1.329180" y3="0.320280" z3="0.572638"/>
<atom id="a8" elementType="H" x3="-2.078222" y3="1.101803" z3="0.733317"/>
<atom id="a9" elementType="C" spinMultiplicity="2" x3="-0.939686" y3="-0.242370" z3="1.892940"/>
<atom id="a10" elementType="H" x3="-1.631171" y3="-0.237957" z3="2.722105"/>
<atom id="a11" elementType="H" x3="-0.015433" y3="-0.793008" z3="1.992786"/>
<atom id="a12" elementType="C" x3="0.974518" y3="0.008565" z3="-0.407862"/>
<atom id="a13" elementType="H" x3="0.733317" y3="-0.842620" z3="-1.036151"/>
<atom id="a14" elementType="C" x3="-0.156291" y3="0.983369" z3="-0.211455"/>
<atom id="a15" elementType="C" x3="2.202883" y3="0.090146" z3="0.098368"/>
<atom id="a16" elementType="H" x3="2.530694" y3="0.895480" z3="0.743207"/>
<atom id="a17" elementType="C" x3="3.194823" y3="-0.947357" z3="-0.206321"/>
<atom id="a18" elementType="H" x3="2.833386" y3="-1.756196" z3="-0.872937"/>
<atom id="a19" elementType="C" x3="0.279687" y3="2.220753" z3="0.575627"/>
<atom id="a20" elementType="H" x3="1.098869" y3="2.731761" z3="0.069790"/>
<atom id="a21" elementType="H" x3="0.604219" y3="1.963640" z3="1.584183"/>
<atom id="a22" elementType="H" x3="-0.551189" y3="2.922376" z3="0.660329"/>
<atom id="a23" elementType="C" x3="-0.653065" y3="1.424308" z3="-1.597476"/>
<atom id="a24" elementType="H" x3="0.125493" y3="1.985100" z3="-2.114526"/>
<atom id="a25" elementType="H" x3="-1.524215" y3="2.074922" z3="-1.496643"/>
<atom id="a26" elementType="H" x3="-0.925620" y3="0.572456" z3="-2.217980"/>
<atom id="a27" elementType="O" x3="4.323415" y3="-0.956257" z3="0.217559"/>
</atomArray>
<bondArray>
<bond atomRefs2="a26 a23" order="1"/>
<bond atomRefs2="a24 a23" order="1"/>
<bond atomRefs2="a23 a25" order="1"/>
<bond atomRefs2="a23 a14" order="1"/>
<bond atomRefs2="a13 a12" order="1"/>
<bond atomRefs2="a6 a1" order="2"/>
<bond atomRefs2="a18 a17" order="1"/>

<bond atomRefs2="a12 a14" order="1"/>
<bond atomRefs2="a12 a15" order="2"/>
<bond atomRefs2="a3 a2" order="1"/>
<bond atomRefs2="a5 a2" order="1"/>
<bond atomRefs2="a14 a7" order="1"/>
<bond atomRefs2="a14 a19" order="1"/>
<bond atomRefs2="a17 a15" order="1"/>
<bond atomRefs2="a17 a27" order="2"/>
<bond atomRefs2="a1 a2" order="1"/>
<bond atomRefs2="a1 a7" order="1"/>
<bond atomRefs2="a20 a19" order="1"/>
<bond atomRefs2="a15 a16" order="1"/>
<bond atomRefs2="a2 a4" order="1"/>
<bond atomRefs2="a7 a8" order="1"/>
<bond atomRefs2="a7 a9" order="1"/>
<bond atomRefs2="a19 a22" order="1"/>
<bond atomRefs2="a19 a21" order="1"/>
<bond atomRefs2="a9 a11" order="1"/>
<bond atomRefs2="a9 a10" order="1"/>
</bondArray>
<propertyList>
<property title="File Format">
<scalar>g09</scalar>
</property>
<property dictRef="me:ZPE">
<scalar units="kcal/mol">-73.49010024</scalar>
</property>
<property dictRef="me:rotConsts">
<array units="cm-1">0.048 0.017 0.016</array>
</property>
<property dictRef="me:symmetryNumber">
<scalar>1</scalar>
</property>
<property dictRef="me:vibFreqs">
<array units="cm-1">41.43 58.53 75.79 130.75 139.05 152.97 166.72 181.31 190.17 238.32 253.54 267.38 306.13 316.36 324.83 349.38 388.12 415.00 517.77 522.86 556.80 589.22 610.20 641.11 763.95 841.75 876.80 924.67 954.02 964.74 995.42 1027.18 1030.51 1040.36 1043.59 1065.64 1107.29 1140.52 1178.20 1205.12 1219.35 1267.66 1288.83 1293.97 1353.33 1376.58 1395.07 1410.79 1425.67 1435.61 1462.51 1467.94 1483.55 1496.17 1501.86 1512.28 1526.82 1722.02 1810.28 1819.11 2908.01 3039.10 3045.68 3049.32 3052.39 3117.62 3121.62 3122.64 3129.33 3144.54 3164.79 3171.82 3176.86 3201.71 3274.29</array>
</property>
<property dictRef="me:spinMultiplicity">
<scalar>2.0</scalar>
</property>
<!-- <property dictRef="me:epsilon">
<scalar>600.00</scalar>
</property>
<property dictRef="me:sigma">

<scalar>6.5</scalar>
</property> -->
<property dictRef="me:MW">
<scalar units="amu">167</scalar>
</property>
</propertyList>
<me:energyTransferModel xsi:type="me:ExponentialDown">
<me:deltaEDown units="cm-1">225.0</me:deltaEDown>
</me:energyTransferModel>
<me:DOSCMethod>ClassicalRotors</me:DOSCMethod>
</molecule>
<molecule id="Vinoxy-RO2-prod" description="RO2 product following O2 addition">
<atomArray>
<atom id="a1" elementType="C" x3="-2.772667" y3="0.634202" z3="0.185808"/>
<atom id="a2" elementType="C" x3="-3.965104" y3="-0.256618" z3="-0.056736"/>
<atom id="a3" elementType="H" x3="-3.660789" y3="-1.304783" z3="-0.061522"/>
<atom id="a4" elementType="H" x3="-4.381524" y3="-0.043198" z3="-1.043159"/>
<atom id="a5" elementType="H" x3="-4.723610" y3="-0.089565" z3="0.702607"/>
<atom id="a6" elementType="O" x3="-2.778658" y3="1.495898" z3="1.030149"/>
<atom id="a7" elementType="C" x3="-1.561406" y3="0.361348" z3="-0.669026"/>
<atom id="a8" elementType="H" x3="-1.877970" y3="0.193322" z3="-1.700272"/>
<atom id="a9" elementType="C" x3="-0.363692" y3="1.313928" z3="-0.581701"/>
<atom id="a10" elementType="H" x3="-0.332671" y3="1.810065" z3="0.386489"/>
<atom id="a11" elementType="H" x3="-0.254754" y3="2.062624" z3="-1.360590"/>
<atom id="a12" elementType="C" x3="0.567239" y3="0.089448" z3="-0.647067"/>
<atom id="a13" elementType="H" x3="0.833194" y3="-0.133144" z3="-1.683156"/>
<atom id="a14" elementType="C" x3="-0.624927" y3="-0.823581" z3="-0.222750"/>
<atom id="a15" elementType="C" x3="1.830415" y3="0.111057" z3="0.185329"/>
<atom id="a16" elementType="C" x3="2.802208" y3="1.164022" z3="-0.319106"/>
<atom id="a17" elementType="H" x3="3.637495" y3="0.778060" z3="-0.933856"/>
<atom id="a18" elementType="C" x3="-0.765131" y3="-2.118559" z3="-0.999962"/>
<atom id="a19" elementType="H" x3="0.041697" y3="-2.806885" z3="-0.744071"/>
<atom id="a20" elementType="H" x3="-0.730160" y3="-1.939835" z3="-2.075506"/>
<atom id="a21" elementType="H" x3="-1.711418" y3="-2.612503" z3="-0.770429"/>
<atom id="a22" elementType="C" x3="-0.681265" y3="-1.081422" z3="1.277521"/>
<atom id="a23" elementType="H" x3="0.133930" y3="-1.738890" z3="1.581942"/>
<atom id="a24" elementType="H" x3="-1.617147" y3="-1.575355" z3="1.543087"/>
<atom id="a25" elementType="H" x3="-0.623286" y3="-0.162200" z3="1.860547"/>
<atom id="a26" elementType="O" x3="2.657639" y3="2.330667" z3="-0.098807"/>
<atom id="a27" elementType="O" x3="2.438510" y3="-1.195479" z3="0.069833"/>
<atom id="a28" elementType="O" spinMultiplicity="2" x3="3.538617" y3="-1.267764" z3="0.766812"/>
<atom id="a29" elementType="H" x3="1.624120" y3="0.292771" z3="1.240134"/>
</atomArray>
<bondArray>
<bond atomRefs2="a20 a18" order="1"/>
<bond atomRefs2="a8 a7" order="1"/>
<bond atomRefs2="a13 a12" order="1"/>
<bond atomRefs2="a11 a9" order="1"/>
<bond atomRefs2="a4 a2" order="1"/>

<bond atomRefs2="a18 a21" order="1"/>
<bond atomRefs2="a18 a19" order="1"/>
<bond atomRefs2="a18 a14" order="1"/>
<bond atomRefs2="a17 a16" order="1"/>
<bond atomRefs2="a7 a9" order="1"/>
<bond atomRefs2="a7 a14" order="1"/>
<bond atomRefs2="a7 a1" order="1"/>
<bond atomRefs2="a12 a9" order="1"/>
<bond atomRefs2="a12 a14" order="1"/>
<bond atomRefs2="a12 a15" order="1"/>
<bond atomRefs2="a9 a10" order="1"/>
<bond atomRefs2="a16 a26" order="2"/>
<bond atomRefs2="a16 a15" order="1"/>
<bond atomRefs2="a14 a22" order="1"/>
<bond atomRefs2="a3 a2" order="1"/>
<bond atomRefs2="a2 a1" order="1"/>
<bond atomRefs2="a2 a5" order="1"/>
<bond atomRefs2="a27 a15" order="1"/>
<bond atomRefs2="a27 a28" order="1"/>
<bond atomRefs2="a15 a29" order="1"/>
<bond atomRefs2="a1 a6" order="2"/>
<bond atomRefs2="a22 a24" order="1"/>
<bond atomRefs2="a22 a23" order="1"/>
<bond atomRefs2="a22 a25" order="1"/>
</bondArray>
<propertyList>
<property title="File Format">
<scalar>g09</scalar>
</property>
<property dictRef="me:ZPE">
<scalar units="kcal/mol">-102.1127873</scalar>
</property>
<property dictRef="me:rotConsts">
<array units="cm-1">0.040 0.014 0.012</array>
</property>
<property dictRef="me:symmetryNumber">
<scalar>1</scalar>
</property>
<property dictRef="me:vibFreqs">
<array units="cm-1">38.81 54.60 58.23 71.31 87.12 119.03 134.40 148.05 194.38 214.75 227.09 255.95 278.46 297.10 312.83 346.86 383.99 392.29 432.63 459.62 539.36 587.90 607.48 617.24 627.85 733.16 801.90 911.31 931.90 954.88 961.25 969.40 981.64 995.64 1010.55 1024.98 1043.75 1074.66 1115.99 1126.63 1172.46 1186.90 1201.92 1210.05 1239.29 1256.21 1268.93 1275.29 1288.91 1306.47 1324.99 1378.64 1398.48 1409.25 1414.96 1428.82 1434.19 1467.67 1479.93 1491.97 1497.34 1507.81 1516.59 1517.75 1821.16 1853.34 2937.60 3038.25 3048.14 3051.49 3060.47 3074.25 3102.79 3105.84 3110.23 3116.79 3118.17 3119.99 3129.39 3162.04 3170.59</array>
</property>
<property dictRef="me:spinMultiplicity">
<scalar>2</scalar>

</property>
<!-- <property dictRef="me:epsilon">
<scalar>600.00</scalar>
</property>
<property dictRef="me:sigma">
<scalar>6.5</scalar>
</property> -->
<property dictRef="me:MW">
<scalar units="amu">199</scalar>
</property>
</propertyList>
<me:energyTransferModel xsi:type="me:ExponentialDown">
<me:deltaEDown units="cm-1">225.0</me:deltaEDown>
</me:energyTransferModel>
<me:DOSCMethod>ClassicalRotors</me:DOSCMethod>
</molecule>
<molecule id="CriegeeRB_prod" description="Criegee ring-broken product">
<atomArray>
<atom id="a1" elementType="C" x3="-0.036657" y3="-1.391539" z3="0.497366"/>
<atom id="a2" elementType="C" x3="-1.350847" y3="-1.239682" z3="-0.219192"/>
<atom id="a3" elementType="C" x3="1.115359" y3="-0.580840" z3="-0.148319"/>
<atom id="a4" elementType="C" spinMultiplicity="2" x3="0.894948" y3="0.912796" z3="0.035001"/>
<atom id="a5" elementType="H" x3="0.220553" y3="-2.449094" z3="0.488000"/>
<atom id="a6" elementType="H" x3="-0.129493" y3="-1.110421" z3="1.540656"/>
<atom id="a7" elementType="C" x3="0.744979" y3="1.799421" z3="-1.168325"/>
<atom id="a8" elementType="H" x3="0.167262" y3="1.315837" z3="-1.949071"/>
<atom id="a9" elementType="H" x3="1.714898" y3="2.064551" z3="-1.589602"/>
<atom id="a10" elementType="H" x3="0.237385" y3="2.723666" z3="-0.904152"/>
<atom id="a11" elementType="C" x3="1.307054" y3="1.574896" z3="1.322474"/>
<atom id="a12" elementType="H" x3="0.721828" y3="2.472979" z3="1.497853"/>
<atom id="a13" elementType="H" x3="2.354225" y3="1.885424" z3="1.299412"/>
<atom id="a14" elementType="H" x3="1.178326" y3="0.926836" z3="2.184174"/>
<atom id="a15" elementType="C" x3="2.453888" y3="-1.079053" z3="0.421327"/>
<atom id="a16" elementType="H" x3="2.517335" y3="-0.898636" z3="1.492585"/>
<atom id="a17" elementType="H" x3="2.529774" y3="-2.160953" z3="0.314372"/>
<atom id="a18" elementType="C" x3="3.702911" y3="-0.506708" z3="-0.203900"/>
<atom id="a19" elementType="H" x3="4.637354" y3="-0.778465" z3="0.298116"/>
<atom id="a20" elementType="O" x3="3.735662" y3="0.180655" z3="-1.185845"/>
<atom id="a21" elementType="C" x3="-2.402138" y3="-0.552775" z3="0.174839"/>
<atom id="a22" elementType="C" x3="-2.604218" y3="0.299431" z3="1.389236"/>
<atom id="a23" elementType="H" x3="-1.776961" y3="0.197124" z3="2.076538"/>
<atom id="a24" elementType="H" x3="-3.518896" y3="0.009108" z3="1.896390"/>
<atom id="a25" elementType="H" x3="-2.692236" y3="1.344656" z3="1.113921"/>
<atom id="a26" elementType="O" x3="-3.536661" y3="-0.695757" z3="-0.632112"/>
<atom id="a27" elementType="O" spinMultiplicity="2" x3="-4.138697" y3="0.428114" z3="-0.874507"/>
<atom id="a28" elementType="H" x3="-1.449508" y3="-1.769767" z3="-1.153641"/>
<atom id="a29" elementType="H" x3="1.111662" y3="-0.795255" z3="-1.213634"/>
</atomArray>

<bondArray>
<bond atomRefs2="a8 a7" order="1"/>
<bond atomRefs2="a9 a7" order="1"/>
<bond atomRefs2="a29 a3" order="1"/>
<bond atomRefs2="a20 a18" order="2"/>
<bond atomRefs2="a7 a10" order="1"/>
<bond atomRefs2="a7 a4" order="1"/>
<bond atomRefs2="a28 a2" order="1"/>
<bond atomRefs2="a27 a26" order="1"/>
<bond atomRefs2="a26 a21" order="1"/>
<bond atomRefs2="a2 a21" order="2"/>
<bond atomRefs2="a2 a1" order="1"/>
<bond atomRefs2="a18 a19" order="1"/>
<bond atomRefs2="a18 a15" order="1"/>
<bond atomRefs2="a3 a4" order="1"/>
<bond atomRefs2="a3 a15" order="1"/>
<bond atomRefs2="a3 a1" order="1"/>
<bond atomRefs2="a4 a11" order="1"/>
<bond atomRefs2="a21 a22" order="1"/>
<bond atomRefs2="a17 a15" order="1"/>
<bond atomRefs2="a15 a16" order="1"/>
<bond atomRefs2="a5 a1" order="1"/>
<bond atomRefs2="a1 a6" order="1"/>
<bond atomRefs2="a25 a22" order="1"/>
<bond atomRefs2="a13 a11" order="1"/>
<bond atomRefs2="a11 a12" order="1"/>
<bond atomRefs2="a11 a14" order="1"/>
<bond atomRefs2="a22 a24" order="1"/>
<bond atomRefs2="a22 a23" order="1"/>
</bondArray>
<propertyList>
<property title="File Format">
<scalar>g09</scalar>
</property>
<property dictRef="me:ZPE">
<scalar units="kcal/mol">-45.92</scalar>
</property>
<property dictRef="me:rotConsts">
<array units="cm-1">0.049564956 0.012288168 0.011773478</array>
</property>
<property dictRef="me:symmetryNumber">
<scalar>1</scalar>
</property>
<property dictRef="me:vibFreqs">
<array units="cm-1">20.42 42.41 65.08 71.98 89.64 91.45 118.93 125.17
152.95 163 178.81 211.82 254.51 286.5 298 338.34
374.12 405.86 468.69 491.25 524.04 587.89
654.09 712.43 767.69 811.61 858.57 963.11
973.61 1020.17 1021.33 1031.53 1061.17 1081.37
1097.89 1118.37 1142.86 1158.42 1164.43 1180.8

1227.73 1277.95 1291.09 1332.22 1362.32 1393.55
1400.23 1462.92 1497.03 1504.61 1518.69 1527.62
1535.59 1541.71 1551.94 1584.31 1594.97 1597.69
1601.2 1603.89 1610.8 1618.32 1623.05 1892.83
1910.71 3098.55 3099.33 3127.97 3159.76 3170.25
3176.83 3177.89 3183.06 3184.78 3197.01 3210.15
3216.21 3231.67 3235.32 3274.9 3311.17</array>
</property>
<property dictRef="me:spinMultiplicity">
<scalar>2</scalar>
</property>
<!-- <property dictRef="me:epsilon">
<scalar>600.00</scalar>
</property>
<property dictRef="me:sigma">
<scalar>6.5</scalar>
</property> -->
<property dictRef="me:MW">
<scalar units="amu">199</scalar>
</property>
</propertyList>
<me:energyTransferModel xsi:type="me:ExponentialDown">
<me:deltaEDown units="cm-1">225.0</me:deltaEDown>
</me:energyTransferModel>
<me:DOSCMethod>ClassicalRotors</me:DOSCMethod>
</molecule>
<molecule id="TS4_1" description="TS connecting Vinoxy to Ring-Break product">
<atomArray>
<atom id="a1" elementType="C" x3="2.466979" y3="-0.512470" z3="0.055469"/>
<atom id="a2" elementType="C" x3="3.523260" y3="0.562459" z3="0.001219"/>
<atom id="a3" elementType="H" x3="3.098401" y3="1.527829" z3="0.282049"/>
<atom id="a4" elementType="H" x3="3.888410" y3="0.661749" z3="-1.022791"/>
<atom id="a5" elementType="H" x3="4.349700" y3="0.312979" z3="0.660219"/>
<atom id="a6" elementType="O" x3="2.625539" y3="-1.538310" z3="0.667559"/>
<atom id="a7" elementType="C" x3="1.175380" y3="-0.210539" z3="-0.685671"/>
<atom id="a8" elementType="H" x3="1.432590" y3="0.176140" z3="-1.674741"/>
<atom id="a9" elementType="C" x3="0.162589" y3="-1.345889" z3="-0.789751"/>
<atom id="a10" elementType="H" x3="0.219419" y3="-1.982549" z3="0.091909"/>
<atom id="a11" elementType="H" x3="0.276109" y3="-1.978839" z3="-1.669811"/>
<atom id="a12" elementType="C" x3="-1.112141" y3="-0.531218" z3="-0.790031"/>
<atom id="a13" elementType="H" x3="-1.284210" y3="0.069392" z3="-1.680181"/>
<atom id="a14" elementType="C" spinMultiplicity="2" x3="0.286990" y3="0.813201" z3="0.019279"/>
<atom id="a15" elementType="C" x3="-2.193811" y3="-0.758387" z3="0.017299"/>
<atom id="a16" elementType="H" x3="-2.139231" y3="-1.450027" z3="0.849619"/>
<atom id="a17" elementType="C" x3="-3.421560" y3="-0.014497" z3="-0.149111"/>
<atom id="a18" elementType="H" x3="-3.431200" y3="0.680373" z3="-1.015651"/>
<atom id="a19" elementType="C" x3="0.116631" y3="2.174851" z3="-0.567331"/>
<atom id="a20" elementType="H" x3="-0.800749" y3="2.639582" z3="-0.202431"/>
<atom id="a21" elementType="H" x3="0.086551" y3="2.150591" z3="-1.656581"/>

<atom id="a22" elementType="H" x3="0.946581" y3="2.830001" z3="-0.274331"/>
<atom id="a23" elementType="C" x3="0.173090" y3="0.718791" z3="1.507299"/>
<atom id="a24" elementType="H" x3="-0.767080" y3="1.152312" z3="1.848959"/>
<atom id="a25" elementType="H" x3="0.985591" y3="1.276341" z3="1.987539"/>
<atom id="a26" elementType="H" x3="0.228600" y3="-0.309319" z3="1.861959"/>
<atom id="a27" elementType="O" x3="-4.394780" y3="-0.103486" z3="0.570219"/>
</atomArray>
<bondArray>
<bond atomRefs2="a13 a12" order="1"/>
<bond atomRefs2="a8 a7" order="1"/>
<bond atomRefs2="a11 a9" order="1"/>
<bond atomRefs2="a21 a19" order="1"/>
<bond atomRefs2="a4 a2" order="1"/>
<bond atomRefs2="a18 a17" order="1"/>
<bond atomRefs2="a12 a9" order="1"/>
<bond atomRefs2="a12 a15" order="2"/>
<bond atomRefs2="a9 a7" order="1"/>
<bond atomRefs2="a9 a10" order="1"/>
<bond atomRefs2="a7 a14" order="1"/>
<bond atomRefs2="a7 a1" order="1"/>
<bond atomRefs2="a19 a22" order="1"/>
<bond atomRefs2="a19 a20" order="1"/>
<bond atomRefs2="a19 a14" order="1"/>
<bond atomRefs2="a17 a15" order="1"/>
<bond atomRefs2="a17 a27" order="2"/>
<bond atomRefs2="a2 a1" order="1"/>
<bond atomRefs2="a2 a3" order="1"/>
<bond atomRefs2="a2 a5" order="1"/>
<bond atomRefs2="a15 a16" order="1"/>
<bond atomRefs2="a14 a23" order="1"/>
<bond atomRefs2="a1 a6" order="2"/>
<bond atomRefs2="a23 a24" order="1"/>
<bond atomRefs2="a23 a26" order="1"/>
<bond atomRefs2="a23 a25" order="1"/>
</bondArray>
<propertyList>
<property title="File Format">
<scalar>g09</scalar>
</property>
<property dictRef="me:ZPE">
<scalar units="kcal/mol">-67.04812779</scalar>
</property>
<property dictRef="me:rotConsts">
<array units="cm-1">0.061 0.016 0.015</array>
</property>
<property dictRef="me:symmetryNumber">
<scalar>1</scalar>
</property>
<property dictRef="me:vibFreqs">

<array units="cm-1">43.47 51.55 66.35 97.48 134.59 155.39 183.51 195.77 220.58 231.62 253.91 271.04 280.36 356.74 371.74 410.77 469.50 517.74 574.00 599.08 626.34 737.31 804.35 886.88 933.50 951.29 960.24 978.29 999.17 1000.37 1009.95 1022.39 1059.14 1096.42 1102.34 1149.75 1180.48 1195.61 1223.85 1263.85 1288.37 1297.62 1314.10 1337.96 1376.75 1398.70 1413.64 1425.21 1427.77 1466.73 1478.16 1480.02 1482.23 1495.95 1497.83 1507.53 1578.53 1745.85 1824.65 2879.05 3003.87 3016.89 3052.10 3064.01 3077.58 3081.57 3095.02 3118.12 3124.33 3124.97 3137.99 3140.52 3171.71 3186.31</array>
</property>
<property dictRef="me:imFreqs">
<array units="cm-1">555.46 </array>
</property>
<property dictRef="me:MW">
<scalar units="amu">167</scalar>
</property>
</propertyList>
<me:DOSCMethod>ClassicalRotors</me:DOSCMethod>
</molecule>
<molecule id="TS4_2" description="TS connecting Vinoxy to Ring-Break product">
<atomArray>
<atom id="a1" elementType="C" x3="2.414410" y3="-0.657030" z3="-0.020570"/>
<atom id="a2" elementType="C" x3="3.534800" y3="0.333509" z3="-0.213820"/>
<atom id="a3" elementType="H" x3="3.276290" y3="1.275769" z3="0.273120"/>
<atom id="a4" elementType="H" x3="3.680760" y3="0.551069" z3="-1.272260"/>
<atom id="a5" elementType="H" x3="4.451620" y3="-0.053671" z3="0.221420"/>
<atom id="a6" elementType="O" x3="2.551060" y3="-1.645700" z3="0.654940"/>
<atom id="a7" elementType="C" x3="1.090100" y3="-0.335210" z3="-0.698940"/>
<atom id="a8" elementType="H" x3="1.299840" y3="0.023970" z3="-1.708790"/>
<atom id="a9" elementType="C" spinMultiplicity="2" x3="0.110920" y3="-1.467560" z3="-0.742190"/>
<atom id="a10" elementType="H" x3="-0.131570" y3="-1.951900" z3="-1.677150"/>
<atom id="a11" elementType="H" x3="-0.014200" y3="-2.059360" z3="0.153070"/>
<atom id="a12" elementType="C" x3="-1.143040" y3="0.261580" z3="-0.453980"/>
<atom id="a13" elementType="H" x3="-1.305480" y3="0.380180" z3="-1.523160"/>
<atom id="a14" elementType="C" x3="0.221810" y3="0.749660" z3="0.021450"/>
<atom id="a15" elementType="C" x3="-2.247200" y3="0.042730" z3="0.311690"/>
<atom id="a16" elementType="H" x3="-2.204210" y3="0.011890" z3="1.392580"/>
<atom id="a17" elementType="C" x3="-3.533450" y3="-0.229640" z3="-0.301240"/>
<atom id="a18" elementType="H" x3="-3.537810" y3="-0.205300" z3="-1.411030"/>
<atom id="a19" elementType="C" x3="0.472800" y3="2.173930" z3="-0.468180"/>
<atom id="a20" elementType="H" x3="-0.248310" y3="2.858340" z3="-0.019470"/>
<atom id="a21" elementType="H" x3="0.375050" y3="2.241920" z3="-1.552580"/>
<atom id="a22" elementType="H" x3="1.471850" y3="2.516110" z3="-0.196680"/>
<atom id="a23" elementType="C" x3="0.361850" y3="0.677080" z3="1.538240"/>
<atom id="a24" elementType="H" x3="-0.361970" y3="1.336160" z3="2.017400"/>
<atom id="a25" elementType="H" x3="1.356200" y3="0.999970" z3="1.848030"/>
<atom id="a26" elementType="H" x3="0.209320" y3="-0.333280" z3="1.916490"/>
<atom id="a27" elementType="O" x3="-4.552980" y3="-0.465070" z3="0.308090"/>
</atomArray>
<bondArray>

<bond atomRefs2="a8 a7" order="1"/>
<bond atomRefs2="a10 a9" order="1"/>
<bond atomRefs2="a21 a19" order="1"/>
<bond atomRefs2="a13 a12" order="1"/>
<bond atomRefs2="a18 a17" order="1"/>
<bond atomRefs2="a4 a2" order="1"/>
<bond atomRefs2="a9 a7" order="1"/>
<bond atomRefs2="a9 a11" order="1"/>
<bond atomRefs2="a7 a1" order="1"/>
<bond atomRefs2="a7 a14" order="1"/>
<bond atomRefs2="a19 a22" order="1"/>
<bond atomRefs2="a19 a20" order="1"/>
<bond atomRefs2="a19 a14" order="1"/>
<bond atomRefs2="a12 a14" order="1"/>
<bond atomRefs2="a12 a15" order="2"/>
<bond atomRefs2="a17 a27" order="2"/>
<bond atomRefs2="a17 a15" order="1"/>
<bond atomRefs2="a2 a1" order="1"/>
<bond atomRefs2="a2 a5" order="1"/>
<bond atomRefs2="a2 a3" order="1"/>
<bond atomRefs2="a1 a6" order="2"/>
<bond atomRefs2="a14 a23" order="1"/>
<bond atomRefs2="a15 a16" order="1"/>
<bond atomRefs2="a23 a25" order="1"/>
<bond atomRefs2="a23 a26" order="1"/>
<bond atomRefs2="a23 a24" order="1"/>
</bondArray>
<propertyList>
<property title="File Format">
<scalar>g09</scalar>
</property>
<property dictRef="me:ZPE">
<scalar units="kcal/mol">-61.65326907</scalar>
</property>
<property dictRef="me:rotConsts">
<array units="cm-1">0.063 0.016 0.015</array>
</property>
<property dictRef="me:symmetryNumber">
<scalar>1</scalar>
</property>
<property dictRef="me:vibFreqs">
<array units="cm-1">32.86 57.73 76.51 100.66 158.05 181.63 199.13 227.76 241.08 264.86 282.44 307.50 325.42 352.19 382.36 416.87 484.87 485.65 562.99 590.02 617.19 640.79 735.71 779.14 827.00 866.15 938.27 961.41 963.11 993.03 996.80 1004.56 1022.19 1030.88 1063.56 1121.88 1149.49 1182.49 1207.48 1212.64 1255.48 1272.99 1302.87 1329.16 1376.07 1399.92 1414.21 1426.03 1437.01 1465.75 1472.68 1482.22 1489.23 1499.75 1509.90 1517.94 1585.76 1763.34 1821.40 2888.30 3044.52 3054.67 3056.16 3068.23 3119.29 3122.19 3124.51 3128.20 3128.67 3135.68 3162.20 3170.80 3203.60 3267.02</array>
</property>

<property dictRef="me:imFreqs">
<array units="cm-1">545.53</array>
</property>
<property dictRef="me:MW">
<scalar units="amu">167</scalar>
</property>
</propertyList>
<me:DOSCMethod>ClassicalRotors</me:DOSCMethod>
</molecule>
<molecule id="RB-prod1-RO2" description="RO2 product following O2 addition to RBProd1">
<atomArray>
<atom id="a1" elementType="C" x3="-0.120787" y3="1.442346" z3="-0.057744"/>
<atom id="a2" elementType="C" x3="0.180749" y3="2.155555" z3="-1.344596"/>
<atom id="a3" elementType="H" x3="-0.677800" y3="2.104922" z3="-2.015922"/>
<atom id="a4" elementType="H" x3="1.004085" y3="1.638872" z3="-1.843633"/>
<atom id="a5" elementType="H" x3="0.455794" y3="3.188806" z3="-1.154322"/>
<atom id="a6" elementType="O" x3="0.028101" y3="1.963948" z3="1.019262"/>
<atom id="a7" elementType="C" x3="-0.598408" y3="-0.009620" z3="-0.169325"/>
<atom id="a8" elementType="H" x3="-0.562696" y3="-0.303516" z3="-1.219910"/>
<atom id="a9" elementType="C" x3="0.342908" y3="-0.918218" z3="0.638579"/>
<atom id="a10" elementType="H" x3="-0.100094" y3="-1.913824" z3="0.701195"/>
<atom id="a11" elementType="H" x3="0.447528" y3="-0.524286" z3="1.648407"/>
<atom id="a12" elementType="C" x3="1.678684" y3="-1.014525" z3="-0.020340"/>
<atom id="a13" elementType="C" x3="-2.078519" y3="-0.136437" z3="0.245476"/>
<atom id="a14" elementType="C" x3="2.804519" y3="-0.471764" z3="0.435305"/>
<atom id="a15" elementType="H" x3="2.839822" y3="0.086112" z3="1.363697"/>
<atom id="a16" elementType="C" x3="4.057935" y3="-0.604874" z3="-0.318099"/>
<atom id="a17" elementType="H" x3="3.975673" y3="-1.189685" z3="-1.257648"/>
<atom id="a18" elementType="C" x3="-2.321346" y3="-0.054125" z3="1.739331"/>
<atom id="a19" elementType="H" x3="-1.819144" y3="-0.863854" z3="2.265265"/>
<atom id="a20" elementType="H" x3="-3.388258" y3="-0.120795" z3="1.946677"/>
<atom id="a21" elementType="H" x3="-1.941254" y3="0.891232" z3="2.119980"/>
<atom id="a22" elementType="C" x3="-2.967942" y3="0.821586" z3="-0.529047"/>
<atom id="a23" elementType="H" x3="-2.758130" y3="1.847460" z3="-0.227265"/>
<atom id="a24" elementType="H" x3="-4.014890" y3="0.613129" z3="-0.314242"/>
<atom id="a25" elementType="H" x3="-2.806924" y3="0.724382" z3="-1.601150"/>
<atom id="a26" elementType="O" x3="5.111474" y3="-0.130554" z3="0.020671"/>
<atom id="a27" elementType="H" x3="1.709790" y3="-1.553121" z3="-0.965558"/>
<atom id="a28" elementType="O" x3="-2.502880" y3="-1.518873" z3="-0.086085"/>
<atom id="a29" elementType="O" spinMultiplicity="2" x3="-2.415477" y3="-1.800194" z3="-1.349201"/>
</atomArray>
<bondArray>
<bond atomRefs2="a3 a2" order="1"/>
<bond atomRefs2="a4 a2" order="1"/>
<bond atomRefs2="a25 a22" order="1"/>
<bond atomRefs2="a29 a28" order="1"/>
<bond atomRefs2="a2 a5" order="1"/>
<bond atomRefs2="a2 a1" order="1"/>

<bond atomRefs2="a17 a16" order="1"/>
<bond atomRefs2="a8 a7" order="1"/>
<bond atomRefs2="a27 a12" order="1"/>
<bond atomRefs2="a22 a24" order="1"/>
<bond atomRefs2="a22 a23" order="1"/>
<bond atomRefs2="a22 a13" order="1"/>
<bond atomRefs2="a16 a26" order="2"/>
<bond atomRefs2="a16 a14" order="1"/>
<bond atomRefs2="a7 a1" order="1"/>
<bond atomRefs2="a7 a13" order="1"/>
<bond atomRefs2="a7 a9" order="1"/>
<bond atomRefs2="a28 a13" order="1"/>
<bond atomRefs2="a1 a6" order="2"/>
<bond atomRefs2="a12 a14" order="2"/>
<bond atomRefs2="a12 a9" order="1"/>
<bond atomRefs2="a13 a18" order="1"/>
<bond atomRefs2="a14 a15" order="1"/>
<bond atomRefs2="a9 a10" order="1"/>
<bond atomRefs2="a9 a11" order="1"/>
<bond atomRefs2="a18 a20" order="1"/>
<bond atomRefs2="a18 a21" order="1"/>
<bond atomRefs2="a18 a19" order="1"/>
</bondArray>
<propertyList>
<property title="File Format">
<scalar>g09</scalar>
</property>
<property dictRef="me:ZPE">
<scalar units="kcal/mol">-111.1709676</scalar>
</property>
<property dictRef="me:rotConsts">
<array units="cm-1">0.037 0.012 0.011</array>
</property>
<property dictRef="me:symmetryNumber">
<scalar>1</scalar>
</property>
<property dictRef="me:vibFreqs">
<array units="cm-1">32.70 39.05 53.83 70.48 117.54 131.72 140.96 154.06 171.35 199.25 207.61 235.33 255.20 269.90 285.14 320.04 326.63 358.20 397.78 452.01 480.98 537.36 554.05 575.49 608.06 637.86 771.86 802.16 847.09 866.85 955.78 958.82 990.48 1001.34 1014.50 1027.04 1046.88 1056.20 1085.39 1097.47 1150.55 1156.88 1193.45 1214.57 1233.90 1261.54 1286.67 1310.44 1329.19 1345.60 1368.48 1392.24 1403.32 1413.86 1431.97 1433.99 1466.60 1478.31 1487.98 1491.41 1501.57 1505.43 1523.06 1732.83 1816.62 1824.96 2895.48 3049.06 3062.73 3067.95 3071.20 3080.20 3116.39 3124.61 3139.20 3141.72 3147.85 3150.69 3164.08 3173.40 3191.06</array>
</property>
<property dictRef="me:spinMultiplicity">
<scalar>2</scalar>
</property>
<!-- <property dictRef="me:epsilon">

<scalar>600.00</scalar>
</property>
<property dictRef="me:sigma">
<scalar>6.5</scalar>
</property> -->
<property dictRef="me:MW">
<scalar units="amu">199</scalar>
</property>
</propertyList>
<me:energyTransferModel xsi:type="me:ExponentialDown">
<me:deltaEDown units="cm-1">225.0</me:deltaEDown>
</me:energyTransferModel>
<me:DOSCMethod>ClassicalRotors</me:DOSCMethod>
</molecule>
<molecule id="RB-prod2-RO2" description="RO2 product following O2 addition to RBProd1">
<atomArray>
<atom id="a1" elementType="C" x3="1.571171" y3="-0.828590" z3="-0.918620"/>
<atom id="a2" elementType="C" x3="2.858845" y3="-1.573227" z3="-0.715120"/>
<atom id="a3" elementType="H" x3="2.775133" y3="-2.242619" z3="0.142431"/>
<atom id="a4" elementType="H" x3="3.645583" y3="-0.853028" z3="-0.479138"/>
<atom id="a5" elementType="H" x3="3.122188" y3="-2.132558" z3="-1.607963"/>
<atom id="a6" elementType="O" x3="0.988880" y3="-0.819378" z3="-1.975476"/>
<atom id="a7" elementType="C" x3="1.031215" y3="-0.047118" z3="0.283882"/>
<atom id="a8" elementType="H" x3="1.777460" y3="-0.070852" z3="1.079901"/>
<atom id="a9" elementType="C" x3="0.837761" y3="1.406085" z3="-0.120943"/>
<atom id="a10" elementType="C" x3="-1.421139" y3="-0.455339" z3="-0.074025"/>
<atom id="a11" elementType="H" x3="-1.276106" y3="-0.864375" z3="-1.070357"/>
<atom id="a12" elementType="C" x3="-0.272588" y3="-0.680414" z3="0.873910"/>
<atom id="a13" elementType="C" x3="-2.563072" y3="0.175658" z3="0.185030"/>
<atom id="a14" elementType="H" x3="-2.792082" y3="0.622778" z3="1.144037"/>
<atom id="a15" elementType="C" x3="-3.594564" y3="0.296328" z3="-0.854390"/>
<atom id="a16" elementType="H" x3="-3.335643" y3="-0.170017" z3="-1.826178"/>
<atom id="a17" elementType="C" x3="-0.531231" y3="-0.092743" z3="2.262591"/>
<atom id="a18" elementType="H" x3="-1.409131" y3="-0.556379" z3="2.712301"/>
<atom id="a19" elementType="H" x3="-0.699581" y3="0.982935" z3="2.241971"/>
<atom id="a20" elementType="H" x3="0.320235" y3="-0.286670" z3="2.914703"/>
<atom id="a21" elementType="C" x3="-0.080694" y3="-2.199951" z3="1.023147"/>
<atom id="a22" elementType="H" x3="-0.958975" y3="-2.639881" z3="1.494268"/>
<atom id="a23" elementType="H" x3="0.783901" y3="-2.414597" z3="1.653444"/>
<atom id="a24" elementType="H" x3="0.060730" y3="-2.690945" z3="0.060086"/>
<atom id="a25" elementType="O" x3="-4.650845" y3="0.853760" z3="-0.698364"/>
<atom id="a26" elementType="O" x3="2.113493" y3="2.014716" z3="-0.431909"/>
<atom id="a27" elementType="O" spinMultiplicity="2" x3="2.839494" y3="2.178870" z3="0.636351"/>
<atom id="a28" elementType="H" x3="0.266926" y3="1.489950" z3="-1.042885"/>
<atom id="a29" elementType="H" x3="0.376963" y3="1.998386" z3="0.665779"/>
</atomArray>
<bondArray>
<bond atomRefs2="a6 a1" order="2"/>

<bond atomRefs2="a16 a15" order="1"/>
<bond atomRefs2="a5 a2" order="1"/>
<bond atomRefs2="a11 a10" order="1"/>
<bond atomRefs2="a28 a9" order="1"/>
<bond atomRefs2="a1 a2" order="1"/>
<bond atomRefs2="a1 a7" order="1"/>
<bond atomRefs2="a15 a25" order="2"/>
<bond atomRefs2="a15 a13" order="1"/>
<bond atomRefs2="a2 a4" order="1"/>
<bond atomRefs2="a2 a3" order="1"/>
<bond atomRefs2="a26 a9" order="1"/>
<bond atomRefs2="a26 a27" order="1"/>
<bond atomRefs2="a9 a7" order="1"/>
<bond atomRefs2="a9 a29" order="1"/>
<bond atomRefs2="a10 a13" order="2"/>
<bond atomRefs2="a10 a12" order="1"/>
<bond atomRefs2="a24 a21" order="1"/>
<bond atomRefs2="a13 a14" order="1"/>
<bond atomRefs2="a7 a12" order="1"/>
<bond atomRefs2="a7 a8" order="1"/>
<bond atomRefs2="a12 a21" order="1"/>
<bond atomRefs2="a12 a17" order="1"/>
<bond atomRefs2="a21 a22" order="1"/>
<bond atomRefs2="a21 a23" order="1"/>
<bond atomRefs2="a19 a17" order="1"/>
<bond atomRefs2="a17 a18" order="1"/>
<bond atomRefs2="a17 a20" order="1"/>
</bondArray>
<propertyList>
<property title="File Format">
<scalar>g09</scalar>
</property>
<property dictRef="me:ZPE">
<scalar units="kcal/mol">-105.2149597</scalar>
</property>
<property dictRef="me:rotConsts">
<array units="cm-1">0.030 0.013 0.012</array>
</property>
<property dictRef="me:symmetryNumber">
<scalar>1</scalar>
</property>
<property dictRef="me:vibFreqs">
<array units="cm-1">33.83 48.54 57.35 68.66 105.68 131.05 148.05 157.11 175.02 175.73 205.16 249.06 271.09 281.30 306.90 322.90 333.99 357.38 415.13 419.28 506.73 516.47 560.67 588.54 634.42 642.98 784.44 824.04 884.80 926.68 939.26 963.96 985.04 995.46 1019.92 1033.11 1045.80 1055.46 1062.26 1117.89 1140.02 1192.48 1199.57 1214.65 1227.00 1246.72 1292.14 1299.20 1344.00 1360.17 1384.17 1395.88 1404.91 1414.69 1426.91 1439.65 1467.60 1478.43 1481.16 1494.61 1504.72 1512.49 1528.79 1727.20 1814.63 1823.06 2911.05 3046.23 3051.97 3056.63 3083.75 3103.87 3119.75 3122.41 3128.89 3132.05 3137.06 3157.75 3170.59 3174.11 3200.49</array>

</property>
<property dictRef="me:spinMultiplicity">
<scalar>2</scalar>
</property>
<!-- <property dictRef="me:epsilon">
<scalar>600.00</scalar>
</property>
<property dictRef="me:sigma">
<scalar>6.5</scalar>
</property> -->
<property dictRef="me:MW">
<scalar units="amu">199</scalar>
</property>
</propertyList>
<me:energyTransferModel xsi:type="me:ExponentialDown">
<me:deltaEDown units="cm-1">225.0</me:deltaEDown>
</me:energyTransferModel>
<me:DOSCMethod>ClassicalRotors</me:DOSCMethod>
</molecule>
<molecule id="N2">
<atom elementType="N" />
<propertyList>
<property dictRef="me:epsilon">
<scalar>91.85</scalar>
</property>
<property dictRef="me:sigma">
<scalar>3.919</scalar>
</property>
<property dictRef="me:MW">
<scalar units="amu">28.0</scalar>
</property>
</propertyList>
</molecule>
</moleculeList>
<reactionList>
<reaction id="R1">
<reactant>
<molecule ref="AP" role="deficientReactant" />
</reactant>
<reactant>
<molecule ref="Ozone" role="excessReactant" />
</reactant>
<product>
<molecule ref="INT2" role="modelled" />
</product>
<me:transitionState>
<molecule ref="TS1" role="transitionState" />
</me:transitionState>
<me:MCRCMethod name="SimpleRRKM"/>
<me:excessReactantConc>1E18</me:excessReactantConc>

```
</reaction>
<reaction id="R2">
<reactant>
<molecule ref="INT2" role="modelled" />
</reactant>
<product>
<molecule ref="INT-CI" role="modelled" />
</product>
<me:transitionState>
<molecule ref="TS2" role="transitionState" />
</me:transitionState>
<me:MCRCMethod name="SimpleRRKM"/>
<me:tunneling name="Eckart"/>
</reaction>
<reaction id="R3">
<reactant>
<molecule ref="INT-CI" role="modelled" />
</reactant>
<product>
<molecule ref="INT-VHP" role="modelled" />
</product>
<me:transitionState>
<molecule ref="TS3" role="transitionState" />
</me:transitionState>
<me:MCRCMethod name="SimpleRRKM"/>
<me:tunneling>Eckart</me:tunneling>
</reaction>
<reaction id="R4">
<product>
<molecule ref="INT-VHP" role="modelled" />
</product>
<reactant>
<molecule ref="Vinoxy" role="modelled" />
</reactant>
<reactant>
<molecule ref="OH" me:type="excessReactant"/>
</reactant>
<me:FragmentDist xsi:type="me:modPrior">
<me:modPriorOrder>0.5</me:modPriorOrder>
<me:modPriorNexp>0.0</me:modPriorNexp>
<me:modPriorTref>298</me:modPriorTref>
</me:FragmentDist>
<me:excessReactantConc>1E18</me:excessReactantConc>
<me:MCRCMethod xsi:type="me:MesmerILT">
<me:preExponential>2.0e-12</me:preExponential>
<me:activationEnergy units="kcal/mol">0</me:activationEnergy>
<me:nInfinity>0.0</me:nInfinity>
</me:MCRCMethod>
</reaction>
<reaction id="R5">
```

<reactant>
<molecule ref="Vinoxy" me:type="modelled"/>
</reactant>
<reactant>
<molecule ref="O2" me:type="excessReactant" />
</reactant>
<product>
<molecule ref="Vinoxy-RO2-prod" me:type="sink" />
</product>
<me:MCRCMethod name="SimpleBimolecularSink" />
<me:BimolecularLossRateCoefficient>2.0E-12</me:BimolecularLossRateCoefficient>
<me:excessReactantConc>5E18</me:excessReactantConc>
</reaction>
<reaction id="R6">
<reactant>
<molecule ref="Vinoxy" role="modelled" />
</reactant>
<product>
<molecule ref="Vinoxy-RB-prod1" role="modelled" />
</product>
<me:transitionState>
<molecule ref="TS4_1" role="transitionState" />
</me:transitionState>
<me:MCRCMethod name="SimpleRRKM"/>
<me:tunneling name="Eckart"/>
</reaction>
<reaction id="R7">
<reactant>
<molecule ref="Vinoxy-RB-prod1" me:type="modelled"/>
</reactant>
<reactant>
<molecule ref="O2" me:type="excessReactant" />
</reactant>
<product>
<molecule ref="RB-prod1-RO2" me:type="sink" />
</product>
<me:MCRCMethod name="SimpleBimolecularSink" />
<me:BimolecularLossRateCoefficient>2.0E-12</me:BimolecularLossRateCoefficient>
<me:excessReactantConc>5E18</me:excessReactantConc>
</reaction>
<reaction id="R8">
<reactant>
<molecule ref="Vinoxy" role="modelled" />
</reactant>
<product>
<molecule ref="Vinoxy-RB-prod2" role="modelled" />
</product>
<me:transitionState>
<molecule ref="TS4_2" role="transitionState" />
</me:transitionState>

```xml
<me:MCRCMethod name="SimpleRRKM"/>
<me:tunneling name="Eckart"/>
</reaction>
<reaction id="R9">
<reactant>
<molecule ref="Vinoxy-RB-prod2" me:type="modelled"/>
</reactant>
<reactant>
<molecule ref="O2" me:type="excessReactant" />
</reactant>
<product>
<molecule ref="RB-prod2-RO2" me:type="sink" />
</product>
<me:MCRCMethod name="SimpleBimolecularSink" />
<me:BimolecularLossRateCoefficient>2.0E-12</me:BimolecularLossRateCoefficient>
<me:excessReactantConc>5E18</me:excessReactantConc>
</reaction>
<reaction id="R10">
<reactant>
<molecule ref="INT-CI" role="modelled" />
</reactant>
<product>
<molecule ref="CriegeeRB_prod" role="sink" />
</product>
<me:transitionState>
<molecule ref="TS-CI_RB" role="transitionState" />
</me:transitionState>
<me:MCRCMethod name="SimpleRRKM"/>
<me:tunneling>Eckart</me:tunneling>
</reaction>
</reactionList>
<me:conditions>
<me:bathGas>N2</me:bathGas>
<me:PTs>
<me:PTpair units="Torr" P="760.0" T="298.0" precision="qd" />
</me:PTs>
</me:conditions>
<me:modelParameters>
<me:grainSize units="cm-1">100</me:grainSize>
<me:energyAboveTheTopHill>60.0</me:energyAboveTheTopHill>
</me:modelParameters>
<me:control>
<me:MaximumEvolutionTime>1E4</me:MaximumEvolutionTime>
<me:testDOS />
<me:printSpeciesProfile />
<!--<me:testMicroRates />-->
<me:testRateConstant />
<me:printGrainDOS />
<!--<me:printCellDOS />-->
<!--<me:printReactionOperatorColumnSums />-->
```

<!--<me:printTunnellingCoefficients />-->
<me:printGrainkfE />
<!--<me:printGrainBoltzmann />-->
<me:printGrainkbE />
<me:eigenvalues>0</me:eigenvalues>
<!--<me:hideInactive/>-->
</me:control>

</me:mesmer>

Supplementary References

1 A. A. Granovsky, *J. Chem. Phys.*, **2011**, *134*, 214113.

2 A. A. Granovsky, Firefly version 8.2.0, http://classic.chem.msu.su/gran/firefly/index.html.

3 Pfeifle, M.; Ma, Y.T.; Jasper, A.W.; Harding, L.B.; Hase, W.L; Klippenstein, S.J. Nascent energy distribution of the Criegee intermediate CH2OO from direct dynamics calculations of primary ozonide dissociation. *J. Chem. Phys.* **2018**, *148*(17), p.174306.

4 Green, N. J. B.; Robertson, S. H. General master equation formulation of reversible dissociation/association reaction. *Chem. Phys. Lett*. **2014**, *605-606*, 44-46.

5 Shannon, R. J.; Robertson, S. H.; Blitz, M. A.; Seakins, P. W. Bimolecular reactions of activated species: An analysis of problematic HC(O)C(O) chemistry. *Chem. Phys. Lett.* **2016**, *661*, 58-64.

6 Kurtén, T.; Møller, K. H.; Nguyen, T. B.; Schwantes, R. H.; Misztal, P. K.; Su, L.; Wennberg, P. O.; Fry, J. L.; Kjaergaard, H. G. Alkoxy radical bond scissions explain the anomalously low secondary organic aerosols and organonitrate yields from α-pinene + NO_3. *J. Phys. Chem. Lett*. **2017**, *8*, 2826-2834.

7 Kalinowski, J.; Heinonen, P.; Kilpeläinen, I.; Räsänen, M.; Gerber, B. R. Stability of Criegee intermediates formed by the ozonolysis of different double bonds. *J. Phys. Chem. A* **2015**, *119*, 2318-2325.

8 Nguyen, T. L.; Lee, H.; Matthews, D. A.; McCarthy, M. C. Stabilization of the simplest Criegee intermediate from the reaction between ozone and ethylene: A high-level quantum chemical and kinetic analysis of ozonolysis. *J. Phys. Chem. A* **2015**, *119*, 5524-5533.

9 Wheeler, S. E.; Ess, D. H.; Houk, K. N. Thinking out of the black box: Accurate barrier heights of 1,3-dipolar cycloadditions of ozone with acetylene and ethylene. *J. Phys. Chem. A* **2008**, *112*, 1798-1807.

10 Anglada, J. M.; Crehuet, R.; Bofill, J. M. The ozonolysis of ethylene: A theoretical study of the gas-phase reaction mechanism. *Chem. Eur. J.* **1999**, *5*, 1809-1822.

11 Jokinen, T.; Sipilä, M.; Richters, S.; Kerminen, V.- M.; Paasonen, P.; Stratmann, F.; Worsnop, D.; Kulmala, M.; Ehn, M.; Herrmann, H.; Berndt, T. Rapid autoxidation forms

highly oxidized RO_2 radicals in the atmosphere. *Angew. Chem. Int. Ed.* **2014**, *53*, 14596-14600.

12 Vereecken, L.; Nozière, B. H-migration in peroxy radicals under atmospheric conditions. *Atmos. Chem. Phys.* **2020**, *20*, 7429-7458.

13 Frisch, M. J.; Trucks, G. W.; Schlegel, H. B.; Scuseria, G. E.; Robb, M. A.; Cheeseman, J. R.; Scalmani, G.; Barone, V.; Mennucci, B.; Petersson, G. A.; et al. Gaussian 09, revision D.01; Gaussian, Inc.: Wallingford, CT, 2009.

14 Møller, K. H.; Otkjær, R. V.; Hyttinen, N.; Kurtén, T.; Kjaergaard, H. Cost-effective implementation of multiconformer transition state theory for peroxy radical hydrogen shift reactions. *J. Phys. Chem. A* **2016**, *120*, 10072-10087.

15 Møller, K. H.; Otkjaer, R. V.; Chen, J.; Kjaergaard, H. G. Double bonds are key to fast unimolecular reactivity in first-generation monoterpene hydroxy peroxy radicals. *J. Phys. Chem. A* **2020**, *124*, 2885-2896.

16 Bunker, D. L.; Garrett, B.; Kliendienst, T.; Long III, G. S. Discrete simulation methods in combustion kinetics. *Combustion and Flame* **1974**, *23*, 373-379.

17 Gillespie, D. T. A general method for numerically simulating the stochastic time evolution of coupled chemical reactions. *J. Comput. Phys.* **1976**, *22*, 403-434.

18 Atkinson, R.; Hasegawa, D.; Aschmann, S. M. Rate constants for the gas-phase reactions of O3 with a series of monoterpenes and related compounds at 296 ± 2 K. *Int. J. Chem. Kinet.* **1990**, *22*, 871-887.

19 Berndt, T.; Scholz, W.; Mentler, B.; Fischer, L.; Herrmann, H.; Kulmala, M.; Hansel, A. Accretion product formation from self- and cross-reactions of RO_2 radicals in the atmosphere. *Angew. Chem. Int. Ed.* **2018**, *57*, 3820-3824.

20 Vereecken, L.; Peeters, J. A structure-activity relationship for the rate coefficient of H-migration in substituted alkoxy radicals. *Phys. Chem. Chem. Phys.* **2010**, *12*, 12608-12620.
